# The evaluation of the effect of estrogen administration on cutaneous wound healing in *Staphylococcus aureus*-infected diabetic and nondiabetic mice

**DOI:** 10.1371/journal.pone.0339341

**Published:** 2025-12-30

**Authors:** Kanae Mukai, Kohei Ogura, Yukari Nakajima, Toshio Nakatani

**Affiliations:** 1 Faculty of Health Sciences, Institute of Medical, Pharmaceutical and Health Sciences, Kanazawa University, Kanazawa, Ishikawa, Japan; 2 Advanced Health Care Science Research Unit, Institute for Frontier Science Initiative, Kanazawa University, Ishikawa, Japan; Southern Medical University Nanfang Hospital, CHINA

## Abstract

Diabetes-related infection has become a difficult and significant global public health issue. Estrogen has specific hormones that promote cutaneous wounds in diabetic mice*.* However, the impact of estrogen on skin-colonized pathogenic bacteria is unknown. The purpose of this study was to look into how estrogen affects wounds infected with *Staphylococcus aureus*. Nondiabetic *db/ +* mice and diabetic *db/db* mice were both injured, and bacterial suspension was applied to each wound site. Estrogen or vehicles were injected intraperitoneally every 3–4 days after wounding. S. *aureus* infection impaired diabetic wounds and reduced collagen deposition. Immunostaining revealed that *S. aureus* infection reduced the number of blood and lymphatic vessels while increasing the number of neutrophils in nondiabetic wounds, regardless of estrogen treatment. Conversely, estrogen administration reduced the number of macrophages in *S. aureus-*infected nondiabetic wounds compared to vehicle-treated wounds. The number of lymphatic vessels was roughly double that of estrogen administration in *S. aureus*-infected nondiabetic wounds. Our findings showed that *S. aureus* infection in diabetic wounds delayed cutaneous wound healing due to excessive inflammation, inhibited collagen deposition, and impaired angiogenesis or lymphangiogenesis, although estrogen administration did not reverse these effects. Our findings also revealed that estrogen administration effectively treated *S. aureus*-infected nondiabetic wounds by regulating the immune response and increasing the synthesis of lymphatic vessels*.*

## Introduction

Diabetes mellitus individuals with foot ulcers had a higher mortality rate [1,2] and high incidence of lower extremity amputation [3,4] than diabetes mellitus patients without ulceration. Diabetic ulcers are regarded as major issues in wound care. Diabetes disrupts cutaneous wound healing by causing prolonged and excessive inflammation [5] or insufficient angiogenesis [6]. Vascular changes and insufficient oxygen delivery also impair leukocyte migration into wounds, raising the risk of infections [7]. Bacterial infection is a major cause of impaired diabetic wound healing [8]. Furthermore, individuals with diabetes mellitus who have diabetic wounds have a 50% higher risk of amputation compared to those who do not have infections [9]. As a result, diabetes-related infections have emerged as a complex and significant global public health issue.

According to the microbiological profile of infected diabetic foot ulcers, the majority of the isolates are gram-positive bacteria (67.5%), with the frequently isolated species being *Staphylococcus aureus* (*S. aureus*), accounting for 16.8% [10]. Several other studies have also found that *S. aureus* was the most commonly isolated pathogen, with frequencies of 17.0% [10], 38.4% [11], 76% [12], and 58% [13]. Furthermore, a recent cohort study found that *S. aureus* was responsible for the persistent infection of diabetic ulcer osteomyelitis [14].

Estrogen is a hormone that promotes cutaneous wound healing in menopausal women [15] and older people [16]. Microarray analyses have revealed that estrogenic sex hormones play an important role in cutaneous wound healing [17]. A recent review also reported that estrogen plays a pivotal role in the clinical treatment of skin wounds due to its multifaceted effects on the wound healing process [18]. In animal studies, estrogen administration promotes cutaneous wound healing even in the presence of delayed systemic factors such as aging [19], malnutrition [20], and diabetes mellitus [21,22]. Previous research has shown that estrogen promotes cutaneous wound healing in *Klebsiella. pneumoniae* (*K. pneumoniae*) infected wounds [23]. *K. pneumoniae* is a gram-negative bacterium found in surgical wounds [24] and burns [25], but is uncommon in infected diabetic wounds. To date, the effect of estrogen on *S. aureus*, which is often isolated in diabetic ulceration [10–13] infected diabetic conditions, is unknown.

A global epidemiology analysis using a systematic review and meta-analysis revealed that the global prevalence of diabetic ulceration was higher in type 2 diabetics than in type-1 diabetics [26]. To conduct basic research on diabetic, genetically induced, or high-fat feeding rodents with hyperglycemia, which are commonly used as type-2 diabetes models. Among these type-2 diabetes models, genetically induced *db/db* mice show the following characteristics of human type-2 diabetes: obesity caused by hyperglycemia, insulin resistance, polydipsia, and polyuria [27]. Furthermore, *db/db* mice have demonstrated the pathogenesis of human type-2 diabetes complications, including susceptibility to infection and impaired wound healing [28,29]. As a result, in this study, we used db*/db* mice for the type-2 diabetes model as well as its heterozygous (*db/+*) mice, and we investigated the effect of estrogen in *S. aureus*-infected with wounds from db/db diabetic and *db/ +* nondiabetic mice.

## Materials and methods

### Animals

A total of twenty 10-week-old female *db/ +* mice (C57BLKS/J Iar- + m+ / + Lepr^db^) and twenty 10-week-old female *db/db* mice (C57BLKS/J Iar- + Lepr^db^/ + Lepr^db^) with diabetes from Sankyo Lab Service, Tokyo, Japan, were included in the experiments. Blood sugar levels were above 400 mg/dL in *db/db* mice and around 200 mg/dL in *db/ +* mice (S1 Fig). Individuals were placed in an air-conditioned room at 25.0°C ± 2.0°C with lights turned on from 08:45–20:45. Water and food were freely provided.

### *Staphylococcus aureus* (*S. aureus*) culture collection

*S. aureus* strain N315 (ST5-SCC*mec* II lineage) was supplied by Dr. Keiichi Hiramatsu [30]. *S. aureus* was cultured overnight at 37°C in Todd-Hewitt broth (Becton and Dickinson, NJ, USA) with 0.5% yeast extract (Becton and Dickinson) (THY broth medium) overnight at 37°C as per a previous study [31]. Bacterial cells were centrifuged and washed with sterile phosphate-buffered saline (PBS) when the culture reached the exponential phase (OD_600_ = 0.5).

### Wounding and *S. aureus* application

Mice were anesthetized with 1.5%–2.0% isoflurane (FUJIFILM Wako Pure Chemical, Tokyo, Japan) and 1.5 L of O_2_/min through a plastic tube mask. The dorsal fur was removed with clippers and depilatory cream on the day of the wounds, and the dorsum was cleaned with alcohol. Estradiol benzoate (estra-1,3,5(10)-triene-3,17β-diol 3-benzoate) (OVAHORMON^®^INJECTION; ASKA Pharmaceutical, Tokyo, Japan) at a dose of 1 mg/kg body weight or vehicle (sesame oil; FUJIFILM Wako Pure Chemical) was administered intraperitoneally (i.p.) every 3–4 days at the time of the wound dressing change. Two circular full-thickness skin wounds (with a 4-mm diameter) in the panniculus carnosus muscle were created on either side of the dorsum using a sterile disposable biopsy punch (Kai Industries, Gifu, Japan). Then, 10 μL of bacterial suspension (0.5 × 10^8^ colony-forming units (CFU)/mL *S. aureus*) was spotted directly onto the surface of each wound following previous studies [28,29] and our preliminary data (S2 Fig). The wounds were covered with film dressing (Tegaderm; 3M Health Care, Tokyo, Japan) and changed every 3–4 days. Mice were kept in cages and their health was observed after induction of the infection every 1–2 days. The body weights were recorded at the time before changing the wound dressing or health observation (S3 Fig). The mice were divided into four groups and each group comprised of 10 animals (n = 10): *S. aureus*-infected nondiabetic vehicle administration (SA *db/+*), *S. aureus*-infected nondiabetic estrogen administration (SA *db/ +* estrogen), *S. aureus*-infected diabetic vehicle administration (SA *db/db*), and *S. aureus*-infected diabetic estrogen administration (SA *db/db* estrogen). On days 7 and 14 after wounding (post-infection), five mice from each group were sacrificed with isoflurane overdose and effectiveness of the treatment was evaluated using a variety of parameters. No mice reached the humane endpoint (weights loss over 20% of the initial body weight).

### Bacterial colony counting

The wound dressings were removed from the mice and suspended in tubes containing 30-mL PBS on days 3, 7, and 14. Then, each bacterial solution was vigorously mixed, serially diluted tenfold, and plated on THY agar plates. After incubation at 37°C for 24h, CFU were counted.

### Macroscopic observations

Day 0 was defined as the day wounds were created, and the process of wound healing was monitored until day 14. The wound area was measured using photographs taken on days 0, 3, 7, 10, 12, and 14. We calculated the wound areas with ImageJ (National Institutes of Health, MD, USA) and presented them as the ratio of daily wound area-to-initial wound area on day 0, as in previous studies [22,32].

### Tissue collection

The mice were sacrificed with isoflurane overdose on days 7 and 14. The wound and surrounding intact skin were harvested, and each wound and surrounding intact skin sample was cut in the center. Half of each wound was treated with 4% paraformaldehyde (PFA) (FUJIFILM Wako Pure Chemical) and embedded in tissue-Tek OCT (Sakura Finetek, Tokyo, Japan).

### Staining

At least six serial ice sections were taken from a wound and stained near its center. Subsequently, 5–10-μm-thick ice sections were subjected to hematoxylin and eosin (HE) staining and collagen staining (K-61, Collagen Research Center, Tokyo, Japan), and immunohistologically stained with anti-neutrophil antibody at a dilution of 1:1000 (ab2557; Abcam, Cambridge, UK) to detect neutrophils, anti-Mac-3 antibody at a dilution of 1:1000 (550292; BD Pharmingen, Tokyo, Japan) to identify macrophages, and anti-cluster of differentiation 31 (CD31) antibody at a dilution of 1:50 (550274; BD Pharmingen) to detect blood vessels, and anti-lymphatic vessel endothelial hyaluronan receptor 1 (LYVE-1) antibody at a dilution of 1:1000 (103-PA50; RELIA Tech GmbH, Wolfenbüttel, Germany) to identify lymphatic vessels per the manufacturer’s instructions with a slight modification. Negative control slides were created by removing each primary antibody.

### Microscopic observations

Staining images were transferred to a computer via a digital microscopic camera (DP27-CU; Olympus, Tokyo, Japan). The re-epithelialization ratio (re-epithelialization length/wound length) was calculated using DP27-CU according to previous studies [19,22]. The ratio of collagen deposition (collagen pixels/total wound pixels) was determined using Adobe Photoshop Elements 25.7 (Adobe System, Tokyo, Japan) following previous research [20]. The number of positive inflammatory cells was counted with ImageJ at three wound sites, × 400 magnification, and divided by the area. The number of positive vessels was counted using ImageJ software at three wound sites, × 200 magnification, and divided by the area.

### Ethical statement

Animal experiments in this study were reviewed and approved by the Kanazawa University Animal experiment committee, and they were carried out following the guidelines for the care and use of laboratory animals of Kanazawa University (AP-224323). Bacterial experiments were carried out following the WHO Laboratory biosafety manual and the microorganism safety management regulations of Kanazawa University.

### Statistical analysis

Data were presented as means ± standard error of the mean (SEM) and analyzed using JMP^®^ 12.1.0 (SAS, NC, USA). The means of multiple groups were compared using one-way analysis of variance (ANOVA), followed by *post hoc* pairwise comparisons with the Tukey–Kramer multiple comparison test. A *p-value* of *<*0.05 indicated statistical significance.

## Results

### *S. aureus* bacterial burden

**Fig 1:**
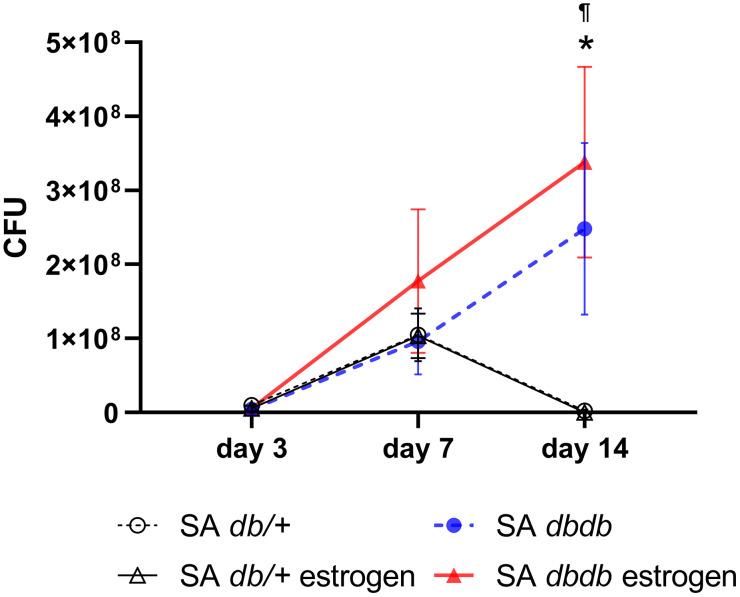
S. aureus colony-forming units. The colony-forming units (CFU) of *S. aureus*at the wound dressing on days 3, 7, and 14 are depicted as a dotted graph. Values are presented as means ± SEM, n = 10 wound dressing; ANOVA, Tukey’s HSD test, *p < 0.05: versus the SA *db/ +* group and ^¶^p < 0.05: versus the SA *db/ +* estrogen group. SA: *S. aureus,* CFU: colony-forming unit.

The *S. aureus* counts were not elevate on day 3 postinfection compared to the initial application (10^6^ CFU) in all four groups. Subsequently, *the S. aureus* bacterial burden differed between nondiabetic and diabetic mice. In the non-diabetes group, the *S. aureus* counts elevated on day 7 (1.0 × 10^8^ ± 4.0 × 10^7^ CFU in the SA *db/ +* group and 1.0 × 10^8^ ± 3.0 × 10^7^ CFU in the SA *db/ +* estrogen group) and then decreased on day 14 (2.0 × 10^6^ ± 2.0 × 10^6^ CFU in the SA *db/ +* group and 3.0 × 10^4^ ± 2.0 × 10^4^ CFU in the SA *db/ +* estrogen group). However, in the diabetes group, *S. aureus* counts were elevated on days 7 (1.0 × 10^8^ ± 4.0 × 10^7^ CFU in the SA *db/db* group and 2.0 × 10^8^ ± 1.0 × 10^8^ CFU in the SA *db/db* estrogen group) and 14 (2.0 × 10^8^ ± 1.0 × 10^8^ CFU in the SA *db/db* group and 3.0 × 10^8^ ± 1.0 × 10^8^ CFU in the SA *db/db* estrogen group). The SA *db/db* estrogen group had significantly larger *S. aureus* counts compared to the SA *db/+* and SA *db/ +* estrogen groups on day 14 (p < 0.05) (Fig 1).

### Body weight

The initial body weights (before wounding) were 22.1 ± 0.31 g in the SA *db/ +* group, 22.0 ± 0.44 g in the SA *db/ +* estrogen group, 38.6 ± 0.81 g in the SA *db/db* group, and 37.3 ± 0.41 g in the SA *db/db* estrogen group, respectively (S3 Fig). After days of wounding, the mean body weights in both vehicle treatment groups decreased by −3.4% in the SA *db/ +* group and −8.4% in the SA *db/db* group until day 14. In contrast, the mean body weights of both estrogen treatment groups increased by 4.8% in the SA *db/ +* estrogen group and 2.2% in the SA *db/db* estrogen group until 14.

### Uterine changes with estrogen administration

The uterus became edematous and enlarged after estrogen administration. On day 14, nutrient vessels were thicker and had more blood in both the SA *db/ +* estrogen and SA *db/db* estrogen groups. Conversely, the uterus did not enlarge in the SA *db/ +* group and atrophied in the SA *db/db* group on day 14 (S4 Fig).

### Macroscopic observations and the wound area

**Fig 2:**
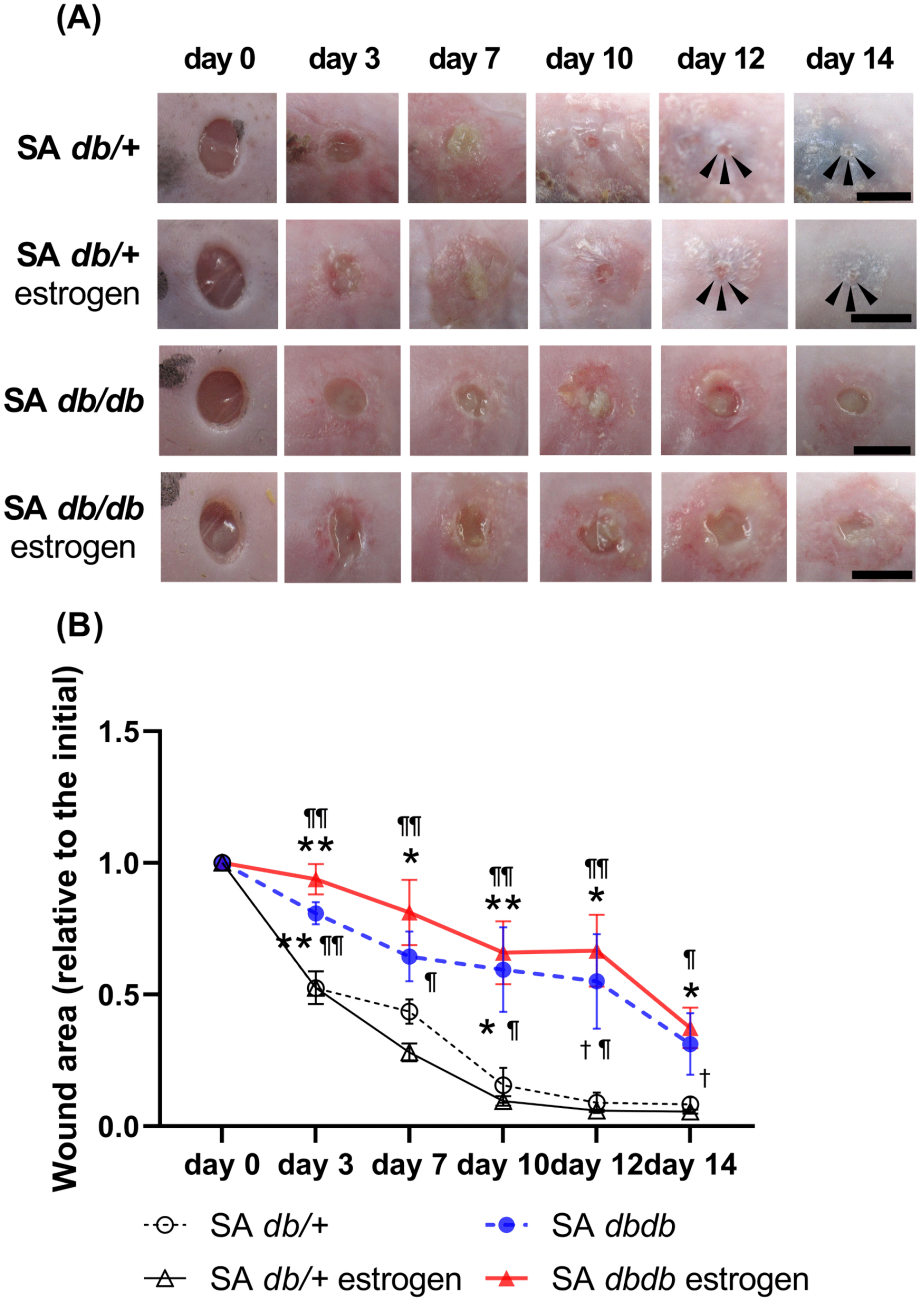
Macroscopic healing of wounds. **(A)** Wounds of 4 mm diameter are created, and images are taken to assess wound healing. Scale bar, 5 mm. **(B)** The wound area-to-initial area ratios on day 0 are depicted as line graphs for each day. Values are presented as means ± SEM, n = 10 wounds; ANOVA, Tukey’s HSD test, **p < 0.01, *p < 0.05 and ^†^p < 0.1: versus the SA *db/ +* group and ^¶¶^p < 0.01,^¶^p < 0.05 and ^†^p < 0.1: versus the SA *db/ +* estrogen group. SA: *S. aureus.*

In nondiabetic group, the necrotic tissues appeared on day 3 and thickly covered the wound surface on day 7. Then, they were gradually removed from the wound surface until day 14 (Fig 2A). The wound areas progressively decreased until day 14 (day 7: 0.44 ± 0.05 in the SA *db/ +* group and 0.28 ± 0.03 in the SA *db/ +* estrogen group; day 14: 0.08 ± 0.03 in the SA *db/ +* group and 0.06 ± 0.01 in the SA *db/ +* estrogen group). In contrast, in diabetic group, necrotic tissues appeared on day 3 and thickened and covered the wound surface until day 14. Furthermore, the peri-wounds displayed redness and maceration (Fig 2A). The wound regions gradually decreased until day 14 (day 7: 0.64 ± 0.09 in the SA *db/db* group and 0.81 ± 0.12 in the SA *db/db* estrogen group, day 14: 0.31 ± 0.12 in the SA *db/db* group and 0.37 ± 0.08 in the SA *db/db* estrogen group). The ratio of wound area in the SA *db/db* group was significantly larger on days 3 and 10 (p < 0.05) and tended to be larger on day 12 (p = 0.080) compared to the SA *db/ +* group, and significantly larger on days 3–12 (p < 0.05) and tended to be larger on day 14 (p = 0.071) comapred to the SA *db/ +* estrogen group. Moreover, the ratio of wound area in the SA *db/db* estrogen group was significantly larger than that of the SA *db/ +* group on days 3–14 (p < 0.05), as well as the SA *db/ +* estrogen group on days 3–14 (p < 0.05) (Fig 2B).

### Re-epithelialization and collagen deposition

**Fig 3:**
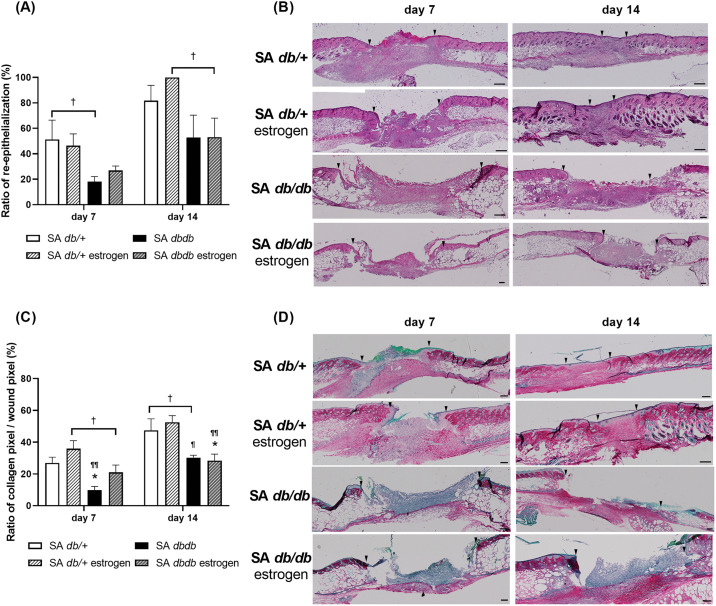
Re-epithelialization and collagen deposition. **(A)** The ratio of re-epithelialization (%) on days 7 and 14 is illustrated as a box graph. Values are presented as means ± SEM, n = 7–9 wounds; ANOVA, Tukey’s HSD test, ^†^p < 0.1: compared to SA *db/+* or SA *db/ +* estrogen groups. (B) hematoxylin and eosin staining (bars, 200 µm) on days 7 and 14. Arrows represent wound edges. **(C)** The ratio of collagen pixel/wound pixel (%) on days 7 and 14 is illustrated in a box graph. Values are presented as means ± SEM, n = 7–9 wounds; ANOVA, Tukey’s HSD test, *p < 0.05 and ^†^p < 0.1: versus the SA *db/ +* group and ^¶¶^p < 0.01, ^¶^p < 0.05 and ^†^p < 0.1: versus the SA *db/ +* estrogen group. **(D)** Collagen staining (bars, 200 µm) on days 7 and 14. Arrows signify the wound edges. SA: *S. aureus.*

In the nondiabetes group, the new epithelium extended from the wound edges on day 7 and covered the wound surface on day 14, with collagen synthesis progressively increasing from day 7 to day 14. Conversely, in diabetic group, the new epithelium did not spread from the wound edges on day 7 and did not cover the wound surface on day 14, and collagen synthesis progressed slowly until day 14. The ratio of re-epithelialization in the SA *db/db* group tended to be lower than that of the SA *db/ +* group on day 7 (p = 0.058), and in the SA *db/db* estrogen group tended to be lower than that of the SA *db/ +* estrogen group on day 14 (p = 0.082) (Fig 3A and 3B). The ratio of collagen deposition in the SA *db/db* group was significantly lower on day 7 (p < 0.05) and tended to be lower on day 14 (p = 0.098) compared to the SA *db/ +* group and significantly lower than that in the SA *db/ +* estrogen group on days 7 and 14 (p < 0.05). The ratio of collagen deposition in the SA *db/db* estrogen group was significantly lower than that in the SA *db/+* and SA *db/ +* estrogen groups on day 14 (p < 0.05) and tended to be lower than that in the SA *db/ +* estrogen group on day 7 (p = 0.070) (Fig 3C and 3D).

### Inflammatory cells

**Fig 4:**
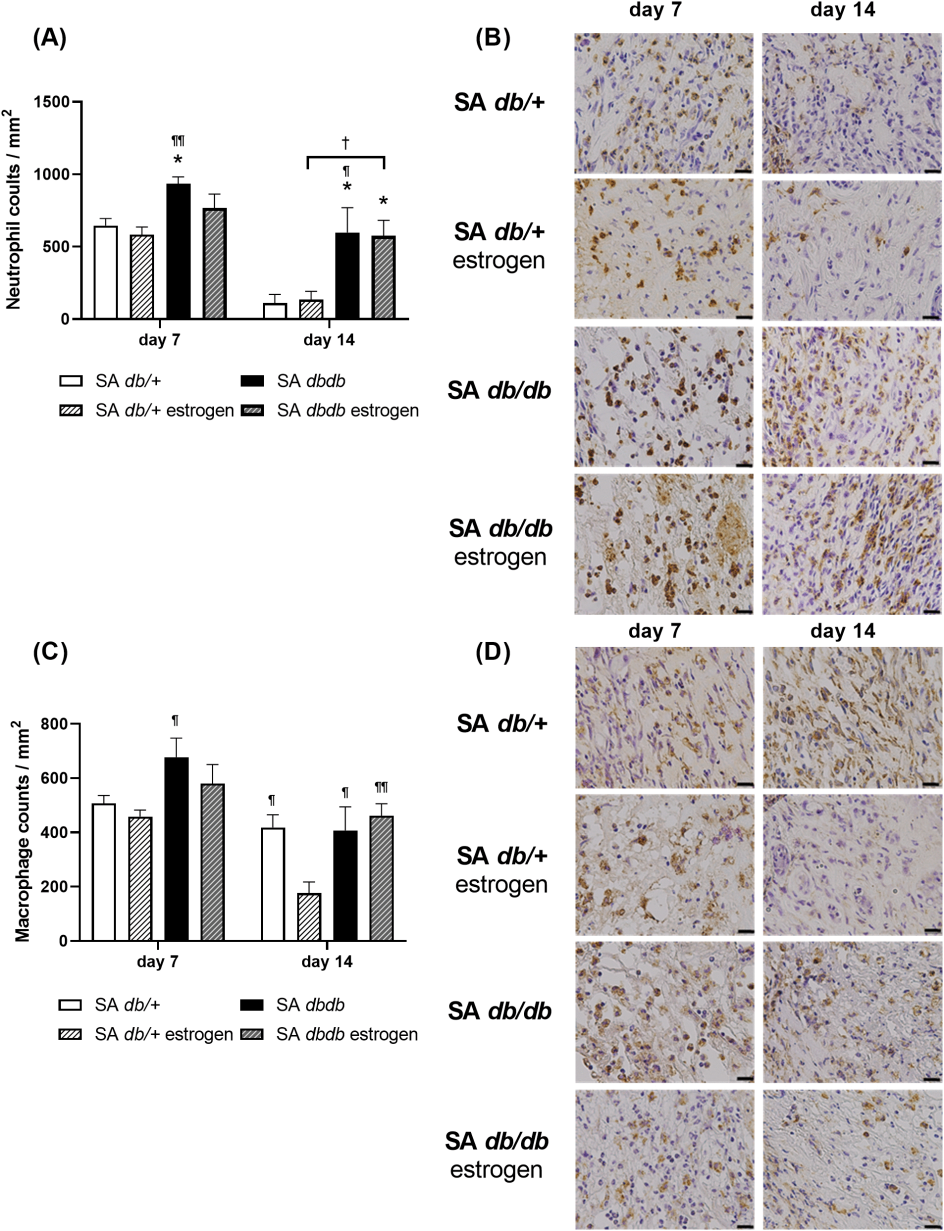
Inflammatory cells. **(A)** The number of neutrophils/mm^2^ on days 7 and 14 is illustrated in a box graph. Values are given as means ± SEM, n = 7–9 wounds; ANOVA, Tukey’s HSD test, *p < 0.05: versus the SA *db/ +* group and ^¶¶^p < 0.01, ^¶^p < 0.05 and ^†^p < 0.1: versus the SA *db/ +* estrogen group. **(B)** Anti-neutrophil antibody immunohistochemistry staining (bars, 20 µm) on days 7 and 14. **(C)** The number of macrophages/mm^2^ on days 7 and 14 is shown in a box graph. Values are presented as means ± SEM, n = 7–9 wounds; ANOVA, Tukey’s HSD test, ^¶¶^p < 0.01 and ^¶^p < 0.05: compared to the SA *db/ +* estrogen group. **(D)** Anti-macrophage antibody immunohistochemistry staining (bars, 20 µm) on days 7 and 14. *S. aureus.*

The nondiabetes group had many inflammatory cells, such as neutrophils and macrophages, at the wounds on day 7. Subsequently, neutrophils and macrophages considerably decreased in the SA *db/ +* estrogen group on day 14. In contrast to the SA *db/ +* group, neutrophils considerably decreased on day 14, whereas macrophages remained at the wounds on day 14. In contrast, in diabetic group, many neutrophils and macrophages were present at the wounds on day 7 and remained on day 14. The neutrophil counts in the SA *db/db* group was significantly higher on days 7 and 14 compared to the SA *db/+* and SA *db/ +* estrogen groups (p < 0.05). The neutrophil counts in the SA *db/db* estrogen group was significantly higher than that in the SA *db/ +* group on day 14 (p < 0.05) and tended to be higher than that in the SA *db/ +* estrogen group on day 14 (p = 0.060) (Fig 4A and 4B). The macrophages counts in the SA *db/db* group was significantly higher than that of the SA *db/ +* estrogen group on days 7 and 14 (p < 0.05). The macrophages counts in the SA *db/db* estrogen group was significantly higher than that of the SA *db/ +* estrogen group on day 14 (p < 0.01). Moreover, the macrophages counts in the SA *db/ +* group was significantly larger than that of the SA *db/ +* estrogen group on day 14 (p < 0.05) (Fig 4C and 4D). Additionally, the relative expression of the *Tnf-α* in the SA *db/db* group was greatly higher than that in the SA *db/+* and SA *db/ +* estrogen groups on day 14 (p < 0.01) (S5 Fig).

### New blood and lymphatic vessels

**Fig 5:**
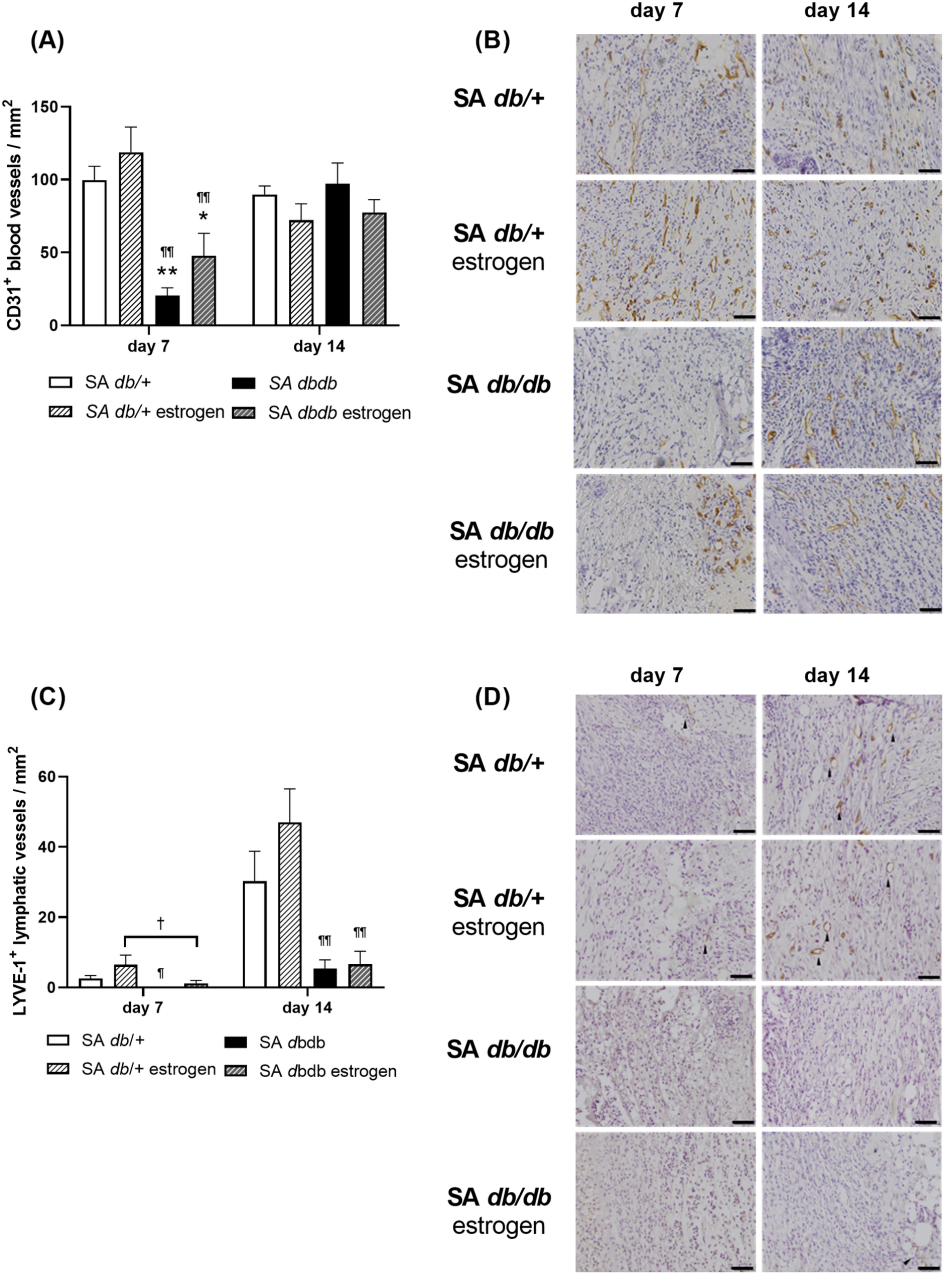
Blood and lymphatic vessels. **(A)** A box graph illustrates the number of CD31 positive blood vessels/mm^2^ on days 7 and 14. Values are given as means ± SEM, n = 7–9 wounds; ANOVA, Tukey’s HSD test, **p < 0.01, *p < 0.05: versus the SA *db/ +* group and ^¶¶^p < 0.01: versus the SA *db/ +* estrogen group. **(B)** Anti-CD31 antibody immunohistochemistry staining (bars, 40 µm) on days 7 and 14. **(C)** The number of LYVE-1 positive lymphatic vessels/mm^2^ on days 7 and 14 is represented in a box graph. Values are presented as means ± SEM, n = 5–9 wounds; ANOVA, Tukey’s HSD test, ^¶¶^p < 0.01, ^¶^p < 0.05, and ^†^p < 0.1: compared to the SA *db/ +* estrogen group. **(D)** Anti-LYVE-1 antibody immunohistochemistry staining (bars, 40 µm) on days 7 and 14. Arrows indicate lymphatic vessels. *S. aureus.*

In the nondiabetes group, many CD31 positive blood vessels appeared at the wounds on day 7, and they were retained on day 14. LYVE-1 positive lymphatic vessels were uncommon on day 7, but there were numerous observed on day 14. In particular, the number of LYVE-1-positive lymphatic vessels in the SA *db/ +* estrogen group was roughly double that of the SA *db/ +* group. Conversely, in diabetic group, CD31 positive blood vessels were rarely seen on day 7, and they gradually increased on day 14. There were no LYVE-1 positive lymphatic vessels observed at the wounds on day 7 and only a few on day 14. The CD31 positive blood vessels in the SA *db/db* and SA *db/db* estrogen groups were significantly lower on day 7 compared to the SA *db/+* and SA *db/ +* estrogen groups (p < 0.05) (Fig 5A and 5B). The LYVE-1 positive lymphatic vessels in the SA *db/db* group was significantly lower than that of the SA *db/ +* estrogen group on days 7 and 14 (p < 0.05). The LYVE-1 positive lymphatic vessels in the SA *db/db* estrogen group tended to be lower on day 7 (p = 0.099) and significantly lower on day 14 (p < 0.01) compared to the SA *db/ +* estrogen group (Fig 5C and 5D).

## Discussion

Diabetic wounds pose a persistent clinical challenge due to delayed cutaneous wound healing and a higher likelihood of infection and complications. Estrogen contains specific hormones that promote cutaneous wounds in diabetic mice [21,22], and those infected with K. *pneumonia* [23]*.* However, the effect of estrogen on *S. aureus*, which is often isolated from species in diabetic infected ulceration [10–13], is unclear. Therefore, this study sought to investigate the effect of estrogen on the wounds of *S. aureus*. As a result, our study found that *S. aureus* infection in diabetic wounds slowed wound healing and was not reversed by estrogen administration. The current findings also revealed that estrogen administration reduced the inflammatory response by lowering the number of macrophages and may promote the formation of new lymphatic vessels in *S. aureus*-infected nondiabetic wound healing*.*

*S aureus*-infected wound models were tested using 10 μL of bacterial suspension (1.0 × 10^7^ CFU/mL) in C57BL/6J and *db/db* mice [28], 20 μL of bacterial suspension (1.0 × 10^8^ CFU/mL) in Balb/c [33], and 10 μL of bacterial suspension (10^8^ CFU/mL) in *db/+* and *db/db* mice [29]. Following these previous studies’ settings, we have set to apply 10 μL of bacteria suspension (0.5 × 10^8^ CFU/mL *S. aureus*) to each wound in *db/+* and *db/db* mice, successfully increasing the number of *S. aureus* on the wound dressing. These findings indicate that the number of bacteria administered in the current setting was sufficient to cause infected wounds. Furthermore, the current study confirmed uterine morphology changes after estrogen administration. The uterus of estrogen-treated mice was edematous and enlarged, with thick, blood-rich nutrient vessels (S4 Fig). Previous studies found that estrogen administration significantly increased uterine size [34] and weight [22]. As a result, the current study concluded that estrogen replacement was sufficient.

Diabetic wounds have an impact on all stages of cutaneous wound healing by increasing inflammatory cell infiltration [35], impairing angiogenesis [36], and degrading extracellular matrix [37]. Wound healing in diabetic mice is slowed by *S. aureus* bacterial species infection [29,38,39]. Similar to previous studies, *S. aureus* infection in diabetic mice did not result in a smaller wound area, and re-epithelialization was incomplete. The current study also found that *S. aureus* infected in diabetic mice had longer inflammation responses and impaired collagen depositions, angiogenesis, and lymphangiogenesis compared to nondiabetic mice. Furthermore, the body weights loss remained within 20% in diabetic and nondiabetic mice. Previous studies had created larger or more wounds than the current study, such as two 8 mm full-thickness [38], 6 mm full-thickness [39], or 3 mm full-thickness at six sites [29]. Weight loss within 14 days was 40% [38], with 14-day overall survival of *S. aureus*-infected diabetic mice at 73% and non-infected diabetic mice at 98% [39]. As a result, it is believed that the adverse events were minor in comparison to previous studies and that *S. aureus* infection in diabetic wounds was delayed.

Unfortunately, estrogen administration did not improve diabetic cutaneous wound healing with *S. aureus* infection. Several previous studies have found that estrogen plays an important immunomodulatory role in microbial infections, directly or indirectly influencing bacterial growth, survival, and virulence [40–42]. However, the number of *S. aureus* was not lowered by estrogen i.p. administration during wound healing in diabetes. It is possible that the promoting effect of estrogen i.p. administration was strongly influenced by *S. aureus* infection in diabetic mice, resulting in delayed wound healing. Meanwhile, the current study found that estrogen administration reduced the macrophages in nondiabetic *S. aureus*-infected wounds, but the healing time was not reduced. Previous research demonstrated that estrogen regulates the innate immune response to *S. aureus* [43,44]. Furthermore, an in vitro study found that estrogen acts as an immune protectant in the monocyte/macrophage response induced by *S. aureus* [45]. As a result, these findings demonstrated that estrogen is effective in regulating immune response to *S. aureus* in nondiabetic wounds.

Lymphangiogenesis and lymphatic vessel remodeling are intricate biological processes [46], and *S. aureus* causes lymphatic dysfunction [47]. In the current study, lymphatic vessels were approximately twice as numerous in nondiabetic *S. aureus*-infected wounds with estrogen administration compared to vehicle administration. Furthermore, they were significantly more numerous than diabetic *S. aureus*-infected wounds. A previous study found that estrogen increases the transcriptional activation of lymphangiogenesis-related gene expression [47]. These findings suggest that estrogen administration may act to promote lymphangiogenesis in nondiabetic wounds against *S. aureus* infection. Impaired lymphangiogenesis in diabetic ulcers causes clinical delays [48], and lymphangiogenesis is being studied as a potential therapeutic target for nonhealing wounds [46]. Therefore, more research is needed to elucidate the lymphangiogenesis mechanism and its relationship with estrogen in nonhealing wound models.

The current study experiments used only one bacterium, *S. aureus* straion N315. The virulence of *S. aureus* varies depending on its virulence factors. For example, Panton–Valentine leukocidin (PVL), a virulence factor produced by some *S. aureus* isolates, is associated with severe skin infection outcomes [49]. Strain N315 belongs to ST5–SCCmec II and possess no *pvl (lukS-PV or lukF-PV)* gene. Whether similar results could be obtained using other bacterial strains or more virulent *S. aureus* species remains unclear. Further studies are needed to establish alternative bacterium-infected wound models. Moreover, the current study employed only i.p. administration. Whether similar results could be obtained with topical estrogen application or a combination with i.p. and topical remains unclear. Further research is required to evaluate the effectiveness of estrogen through other administration routes in bacterium-infected diabetic wounds.

## Conclusion

Our findings showed that *S. aureus*-infected wounds in diabetic mice delayed wound healing by causing excessive inflammation, inhibiting collagen deposition, angiogenesis, and lymphangiogenesis, although these changes did not improve with estrogen administration. Furthermore, the current study found that estrogen administration was effective in treating *S. aureus*-infected wounds in nondiabetic mice by regulating the immune response and increasing the synthesis of lymphatic vessels*.* As a result, such treatment may partially improve healing in nondiabetic *S. aureus*-infected wounds.

## Supporting information

S1 Fig Blood sugar (mg/dL) before and after fasting (2h, 4h and 6h). Values are expressed as means ± SEM, n = 6 mice per group.
(PDF)

S2 Fig Colony-forming units of *S. aureus* in WT mice. The colony forming units (CFU) of S. *aureus* at the wound dressing are shown as a box graph on days 1 (24h), 2 (42h) and 3 (72h). SA: *S. aureus.*
(PDF)

S3 Fig Body weights. The body weights on each day are depicted as a line graph. Values are expressed as means ± SEM. SA: *S. aureus.*
(PDF)

S4 Fig Uterus. The photographs of uterus are shown on day 14. Arrows indicate the atrophied uterus. Bar, 5 mm. SA: *S. aureus.*
(PDF)

S5 Fig Relative expression of inflammatory cytokines. Values are expressed as means ± SEM, n = 7–9 wounds; ANOVA, Tukey’s HSD test, **p < 0.01: versus the SA *db/ +* group and ^¶¶^p < 0.01: versus the SA *db/ +* estrogen group. The primer sequences (*Tnf-α, Il-6*, and *Gapdh*) were used according to previous research (https://doi.org/10.1007/s00403-025-03883-y). SA: *S. aureus.*
(PDF)

S1 File Data set. (ZIP)

S2 File Fig 2A_wound pictures-1.
(ZIP)

S3 File Fig 2A_wound pictures-2.
(ZIP)

S4 File Fig 3B_wound images-1.
(ZIP)

S5 File Fig 3B_wound images-2.
(ZIP)

S6 File Fig 3D_wound images-1.
(ZIP)

S7 File Fig 3D_wound images-2.
(ZIP)

S8 File Fig 4B_wound images.
(ZIP)

S9 File Fig 4D_wound images.
(ZIP)

S10 File Fig 5B_wound images.
(ZIP)

S11 File Fig 5D_wound images.
(ZIP)

S12 File S4 Fig_uterus.
(ZIP)
