# Supplementary material for: The evaluation of the effect of estrogen administration on cutaneous wound healing in Staphylococcus aureus-infected diabetic and nondiabetic mice
Source: PLoS One. 2025 Dec 30;20(12):e0339341. doi: 10.1371/journal.pone.0339341 (PMC12962825; doi:10.1371/journal.pone.0339341)
Supplement: S1 Fig — Values are expressed as means ± SEM, n = 6 mice per group. (PDF) [file pone.0339341.s001.pdf]

**Supporting information**

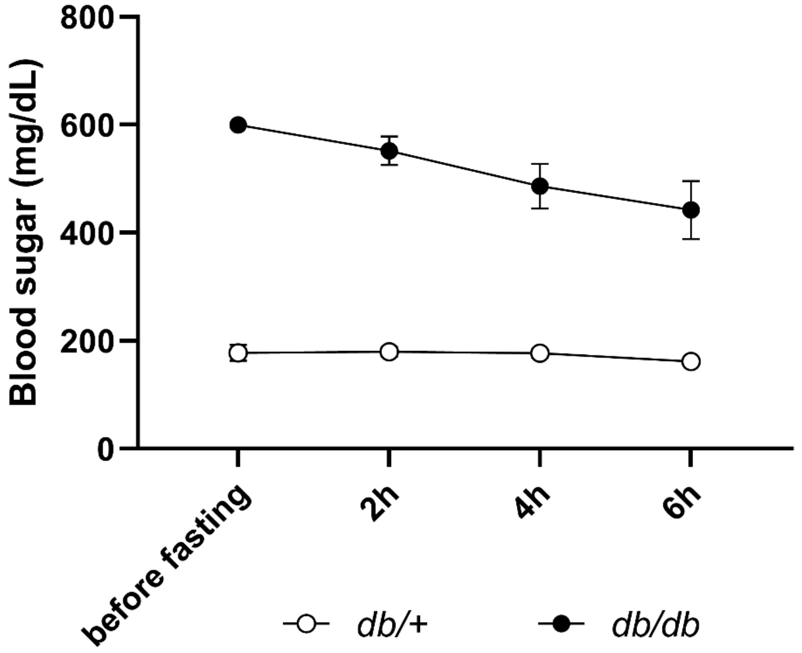


**S1 Fig. Blood sugar (mg/dL) before and after fasting (2h, 4h and 6h).**

Values are expressed as means ± SEM, n = 6 mice per group.
