# Supplementary material for: The evaluation of the effect of estrogen administration on cutaneous wound healing in Staphylococcus aureus-infected diabetic and nondiabetic mice
Source: PLoS One. 2025 Dec 30;20(12):e0339341. doi: 10.1371/journal.pone.0339341 (PMC12962825; doi:10.1371/journal.pone.0339341)
Supplement: S2 Fig — The colony forming units (CFU) of S. aureus at the wound dressing are shown as a box graph on days 1 (24h), 2 (42h) and 3 (72h). SA: S. aureus. (PDF) [file pone.0339341.s002.pdf]

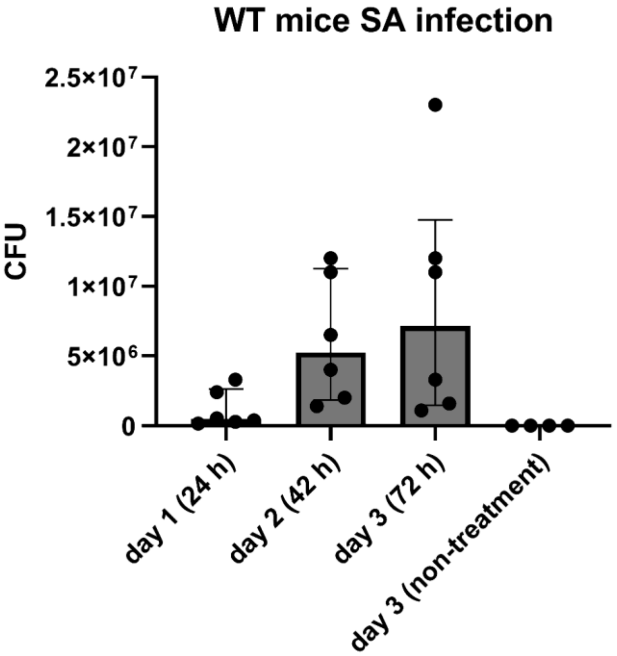

S2 Fig. Colony-forming units of S. *aureus* in the WT mice

The colony forming units (CFU) of S. *aureus* at the wound dressing are shown as a box graph on days 1 (24h), 2 (42h) and 3 (72h). SA: *S. aureus*
