# Supplementary material for: The evaluation of the effect of estrogen administration on cutaneous wound healing in Staphylococcus aureus-infected diabetic and nondiabetic mice
Source: PLoS One. 2025 Dec 30;20(12):e0339341. doi: 10.1371/journal.pone.0339341 (PMC12962825; doi:10.1371/journal.pone.0339341)
Supplement: S3 Fig — The body weights on each day are depicted as a line graph. Values are expressed as means ± SEM. SA: S. aureus. (PDF) [file pone.0339341.s003.pdf]

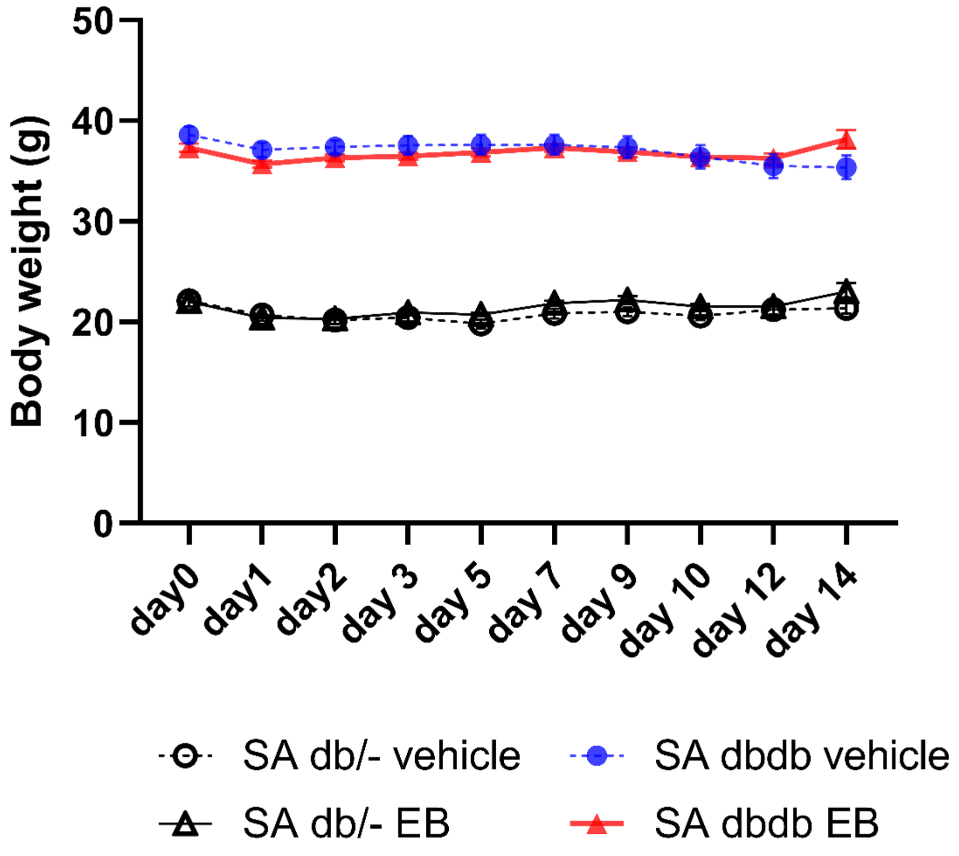

S3 Fig. Body weights.

The body weights on each day are depicted as a line graph. Values are expressed as means ± SEM. SA: *S. aureus*
