# Supplementary material for: The evaluation of the effect of estrogen administration on cutaneous wound healing in Staphylococcus aureus-infected diabetic and nondiabetic mice
Source: PLoS One. 2025 Dec 30;20(12):e0339341. doi: 10.1371/journal.pone.0339341 (PMC12962825; doi:10.1371/journal.pone.0339341)
Supplement: S4 Fig — The photographs of uterus are shown on day 14. Arrows indicate the atrophied uterus. Bar, 5 mm. SA: S. aureus. (PDF) [file pone.0339341.s004.pdf]

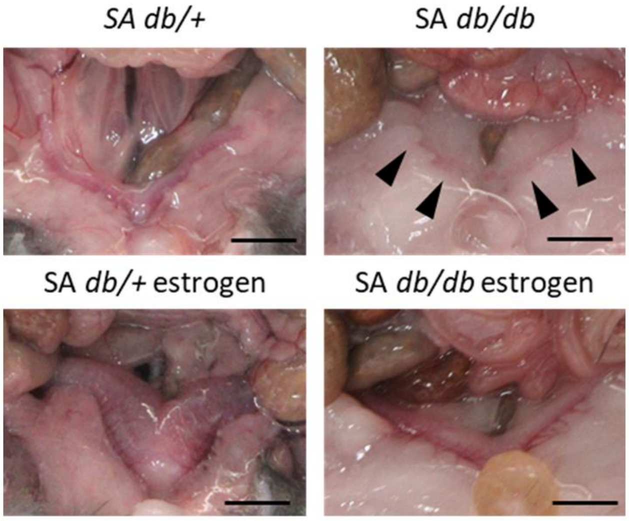

S4 Fig. Uterus.

The photographs of uterus are shown on day 14. Arrows indicate the atrophied uterus.

Bar, 5 mm. SA: *S. aureus*
