# Supplementary material for: The evaluation of the effect of estrogen administration on cutaneous wound healing in Staphylococcus aureus-infected diabetic and nondiabetic mice
Source: PLoS One. 2025 Dec 30;20(12):e0339341. doi: 10.1371/journal.pone.0339341 (PMC12962825; doi:10.1371/journal.pone.0339341)
Supplement: S5 Fig — Values are expressed as means ± SEM, n = 7–9 wounds; ANOVA, Tukey’s HSD test, **p < 0.01: versus the SA db/ + group and ¶¶p < 0.01: versus the SA db/ + estrogen group. The primer sequences (Tnf-α, Il-6, and Gapdh) were used according to previous research (https://doi.org/10.1007/s00403-025-03883-y). SA: S. aureus. (PDF) [file pone.0339341.s005.pdf]

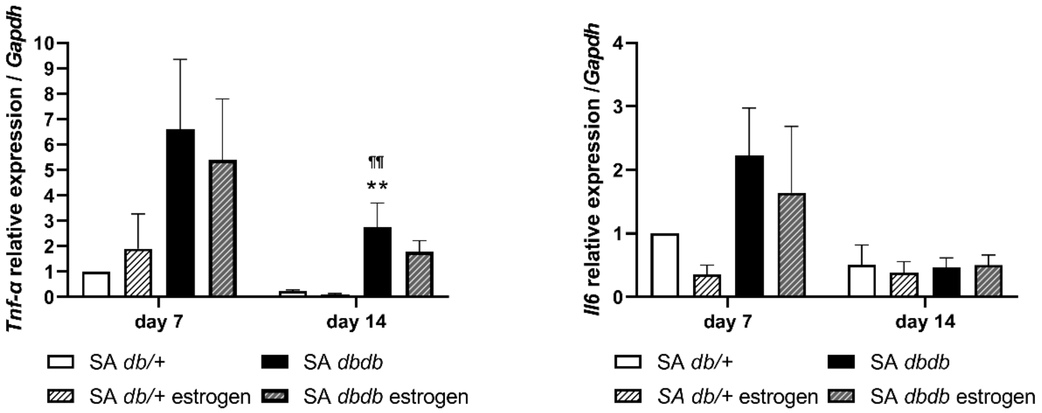

S5 Fig. Relative expression of inflammatory cytokines.

Values are expressed as means ± SEM, n = 7–9 wounds; ANOVA, Tukey's HSD test, **p < 0.01: versus the SA *db/*+ group and ¶¶p < 0.01: versus the SA *db/*+ estrogen group. The primer sequences (*Tnf-α, Il-6*, and *Gapdh*) were used according to previous research (doi:10.1007/s00403-025-03883-y). SA: *S. aureus*
