# Supplementary figures and images for: The evaluation of the effect of estrogen administration on cutaneous wound healing in Staphylococcus aureus-infected diabetic and nondiabetic mice
Source: PLoS One. 2025 Dec 30;20(12):e0339341. doi: 10.1371/journal.pone.0339341 (PMC12962825; doi:10.1371/journal.pone.0339341)

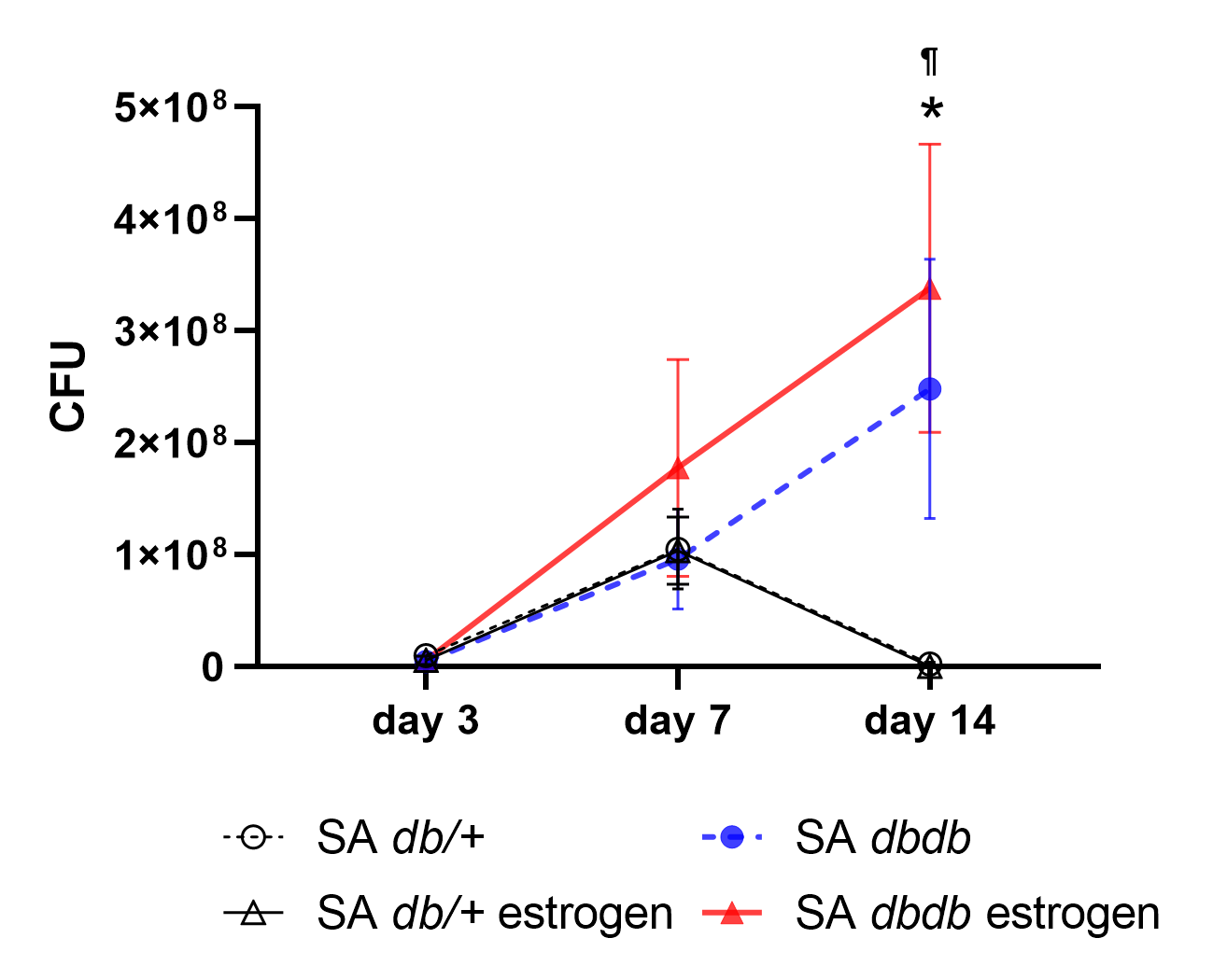

Supplement: S1 File — (ZIP) [file pone.0339341.s006.zip › data set/fig 1_dbdb and db+- SA infection.png]

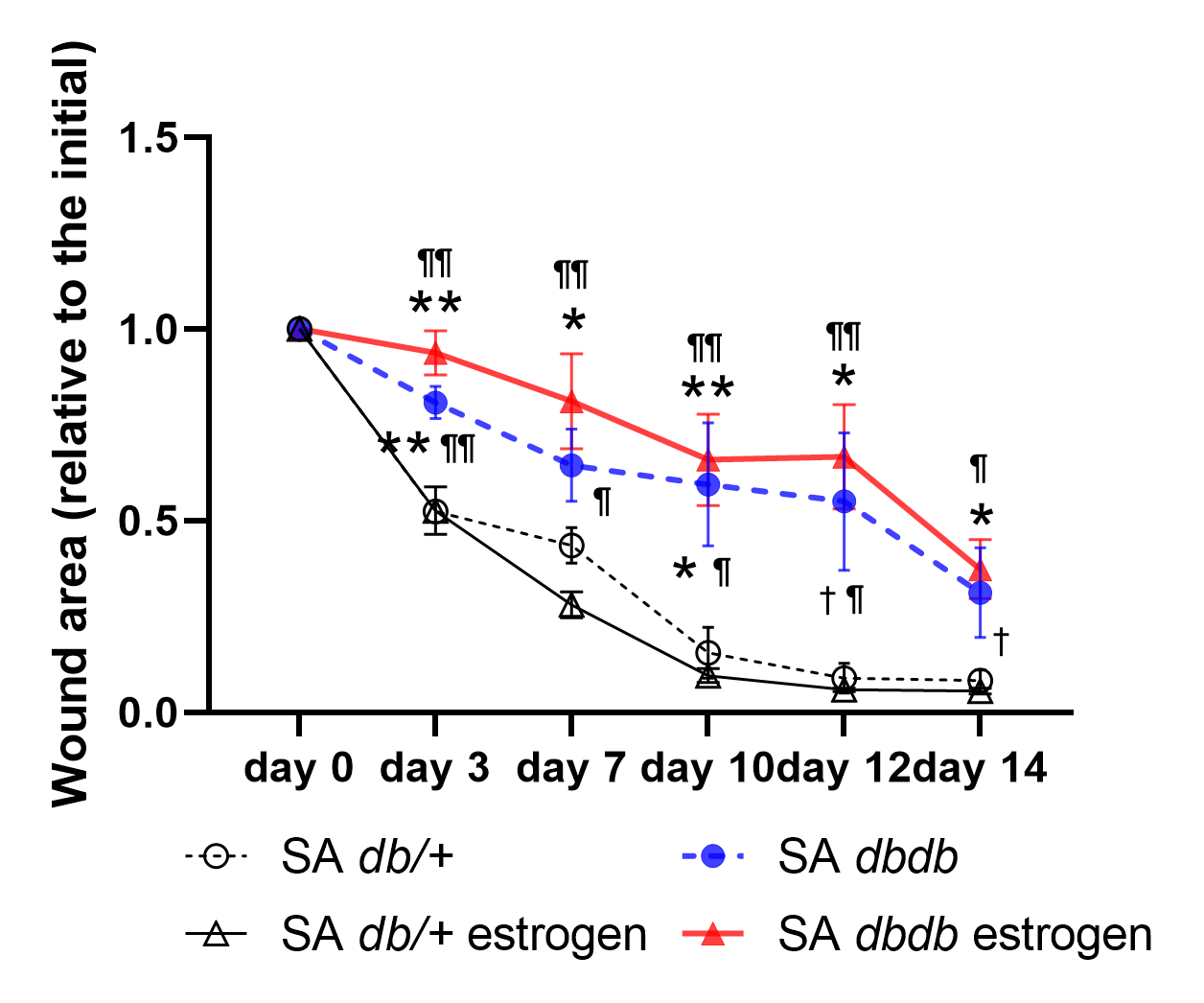

Supplement: S1 File — (ZIP) [file pone.0339341.s006.zip › data set/fig 2B_wound area SA infection.png]

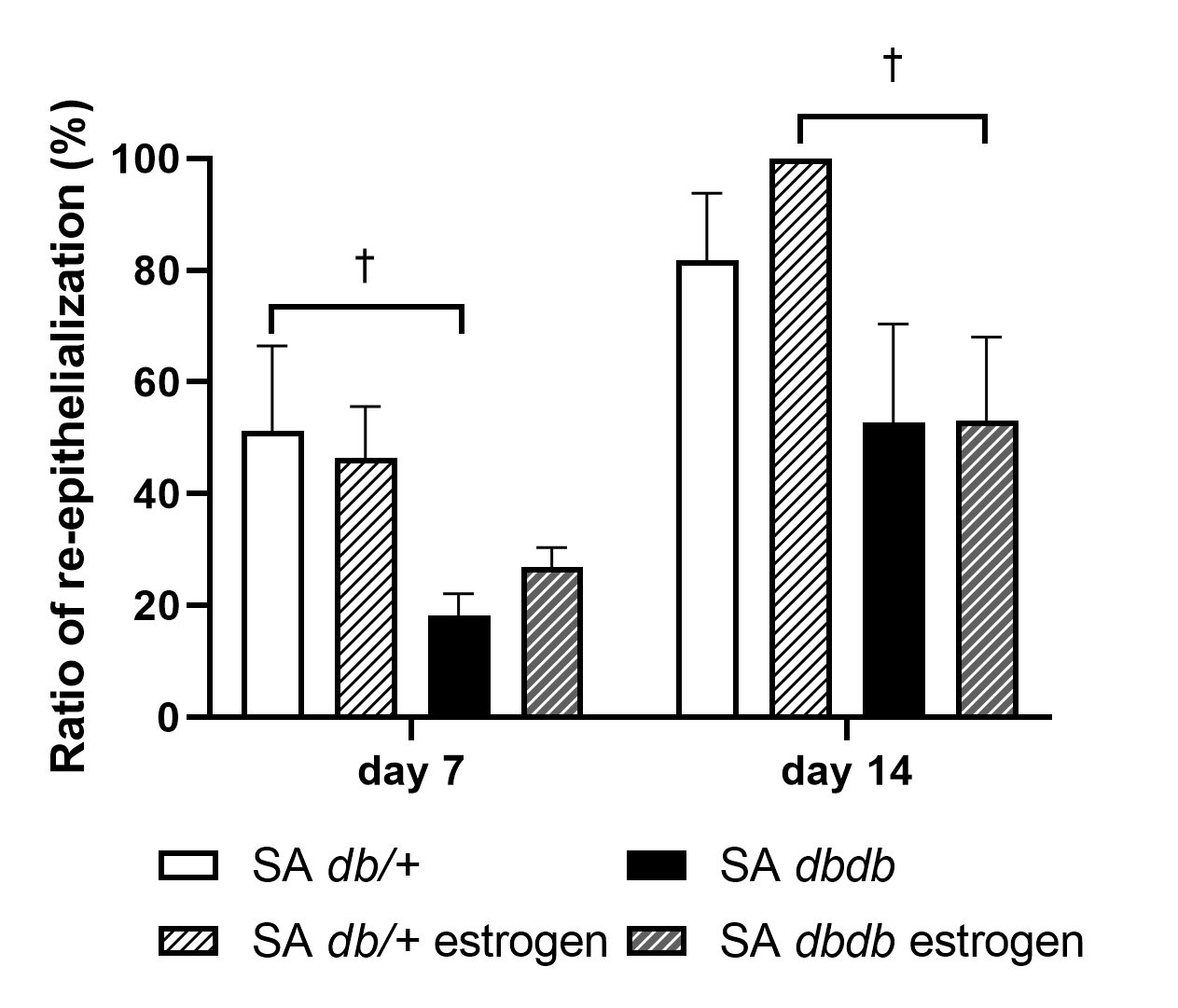

Supplement: S1 File — (ZIP) [file pone.0339341.s006.zip › data set/fig 3A_re-epithelialization SA infection.png]

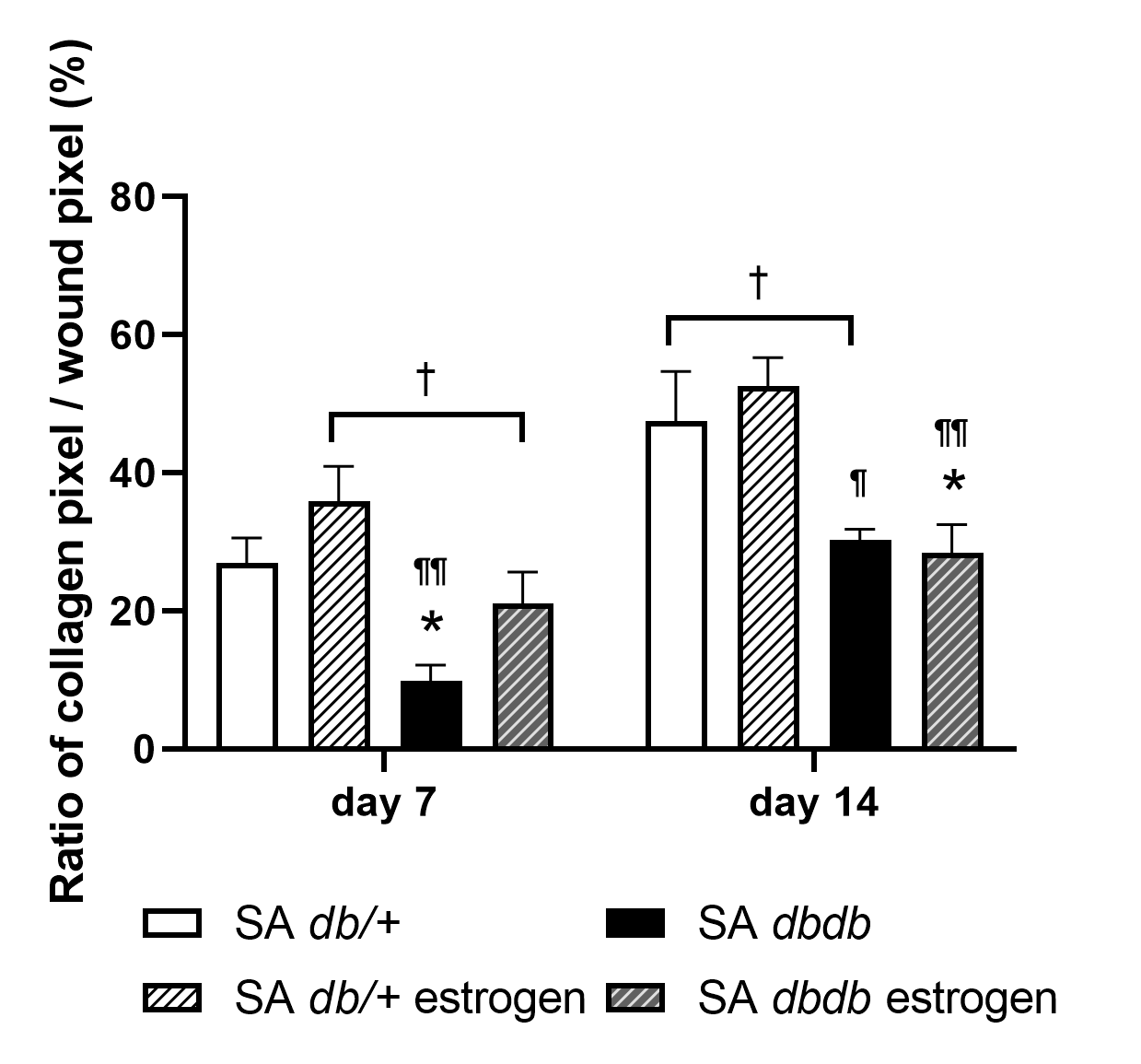

Supplement: S1 File — (ZIP) [file pone.0339341.s006.zip › data set/fig 3C_collagen SA infection.png]

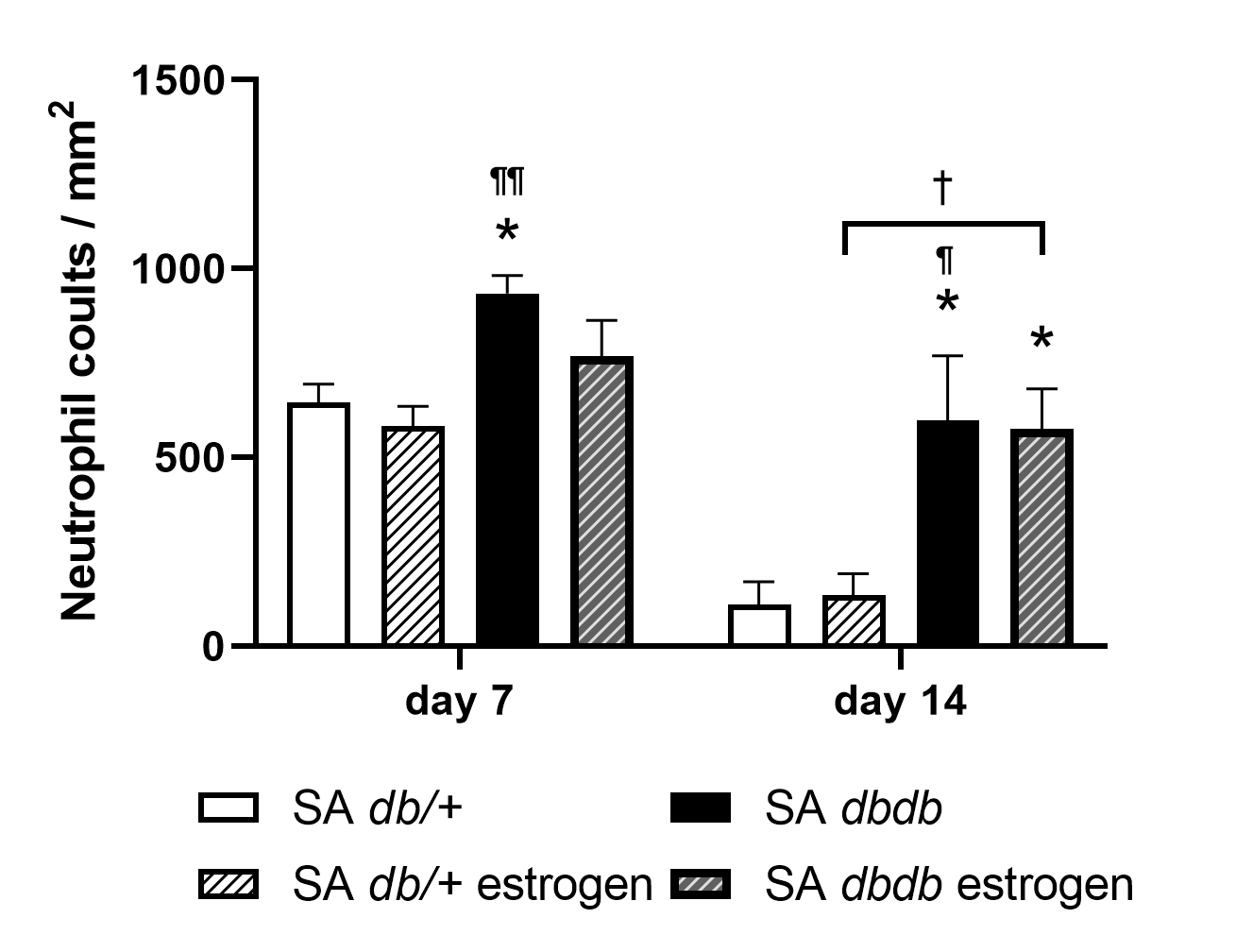

Supplement: S1 File — (ZIP) [file pone.0339341.s006.zip › data set/fig 4A_neutrophils SA infection.png]

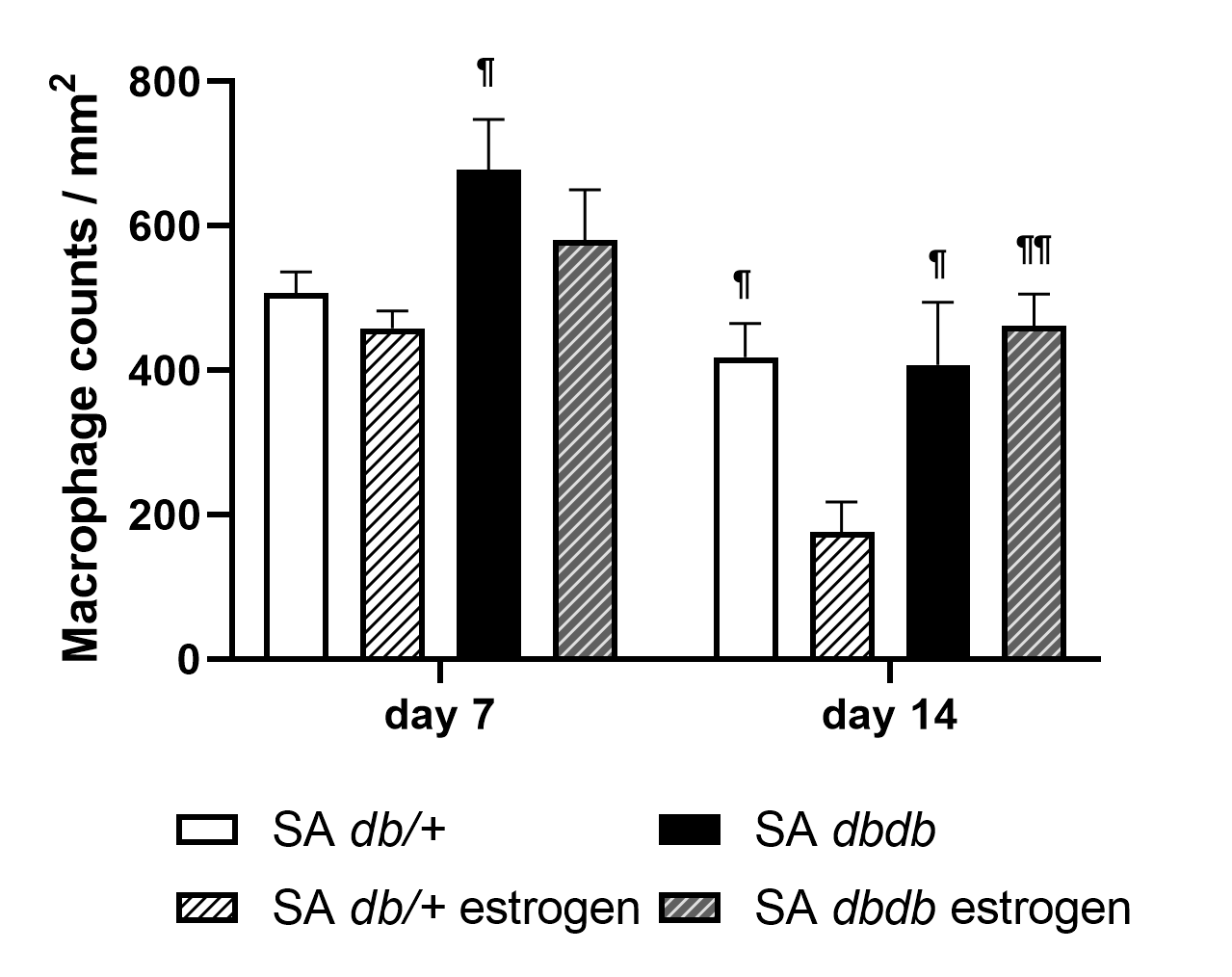

Supplement: S1 File — (ZIP) [file pone.0339341.s006.zip › data set/fig 4C_mac-3 SA infection.png]

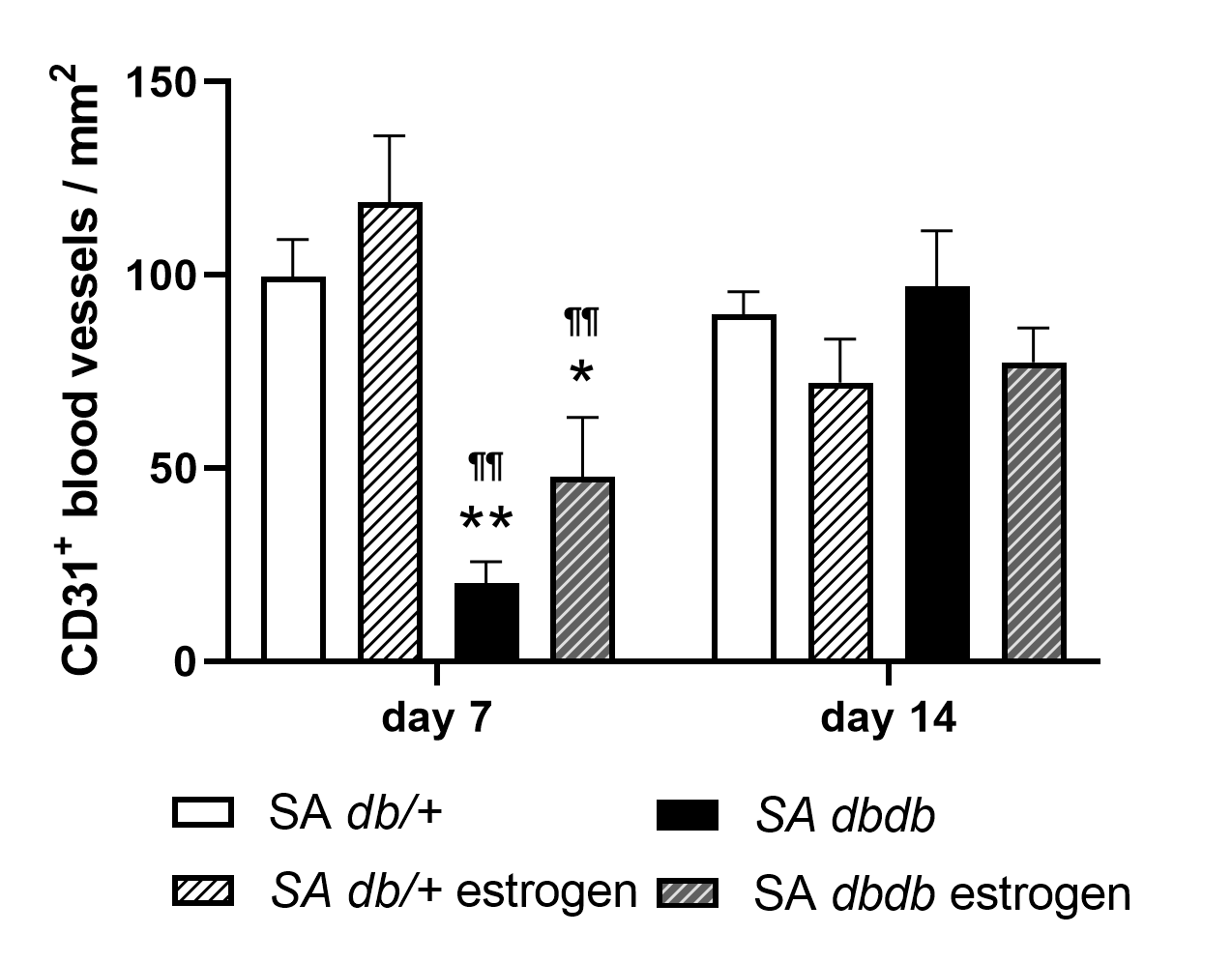

Supplement: S1 File — (ZIP) [file pone.0339341.s006.zip › data set/fig 5A_CD31 SA infection.png]

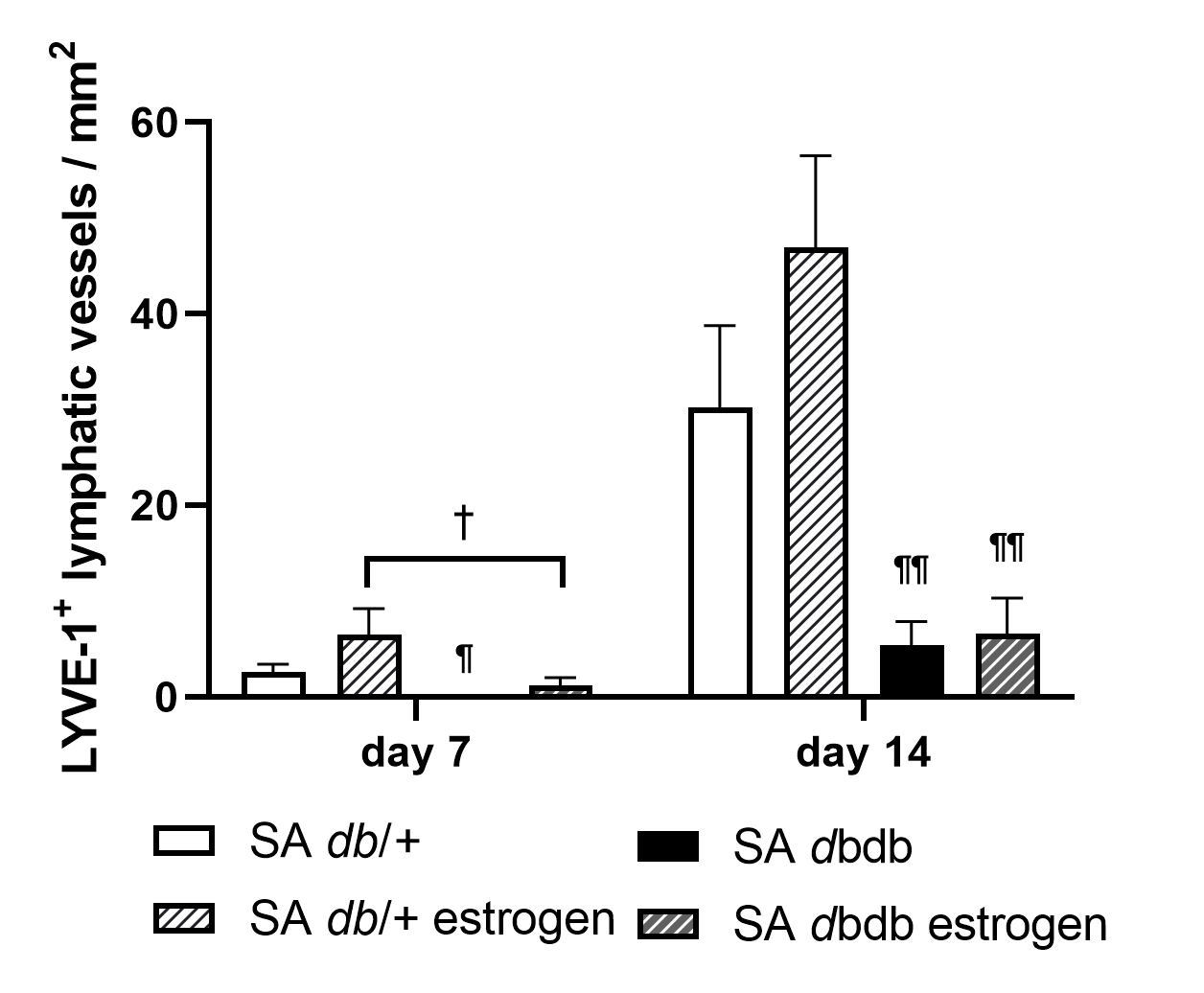

Supplement: S1 File — (ZIP) [file pone.0339341.s006.zip › data set/fig 5C_LYVE-1 SA infection.png]

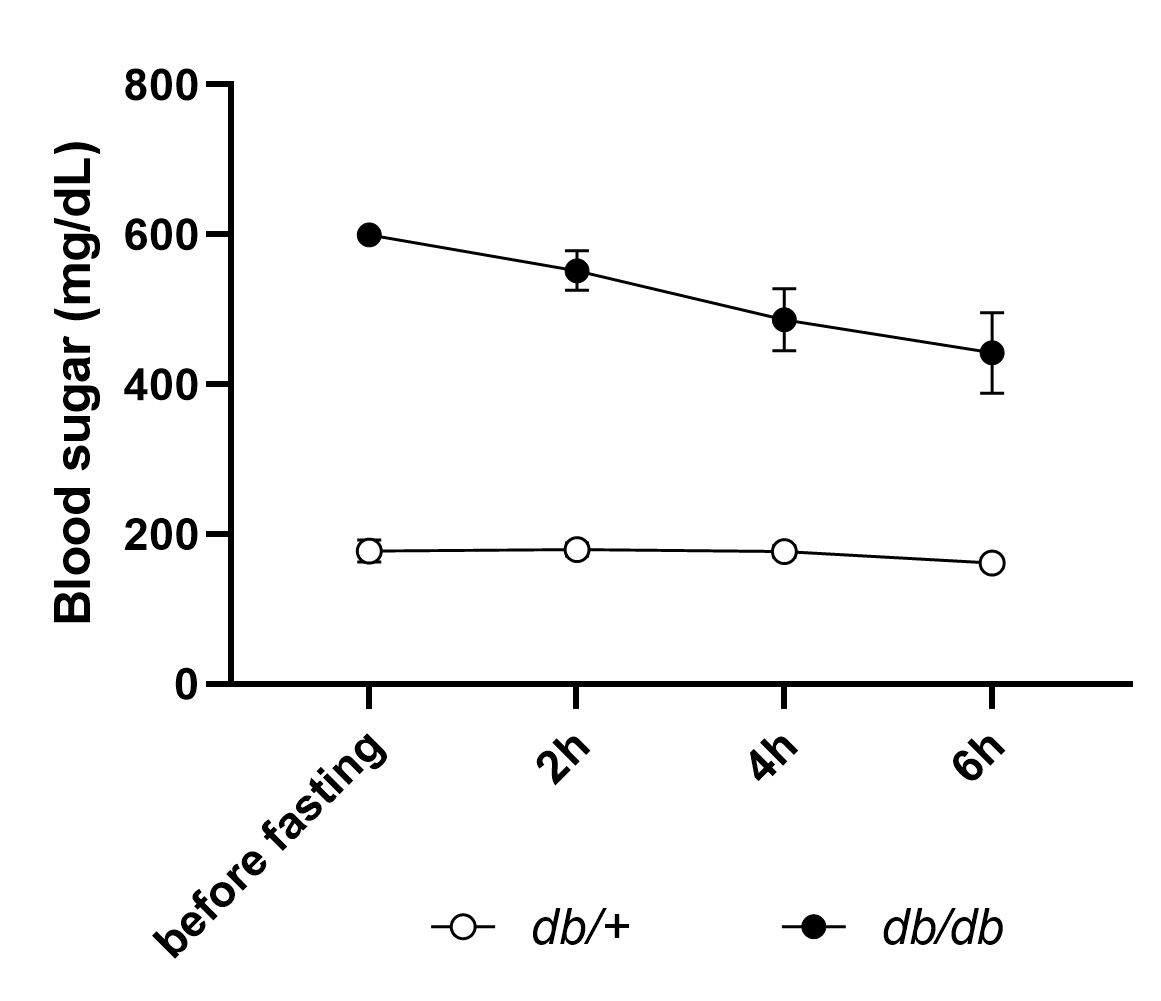

Supplement: S1 File — (ZIP) [file pone.0339341.s006.zip › data set/S1 fig_BS non infection.png]

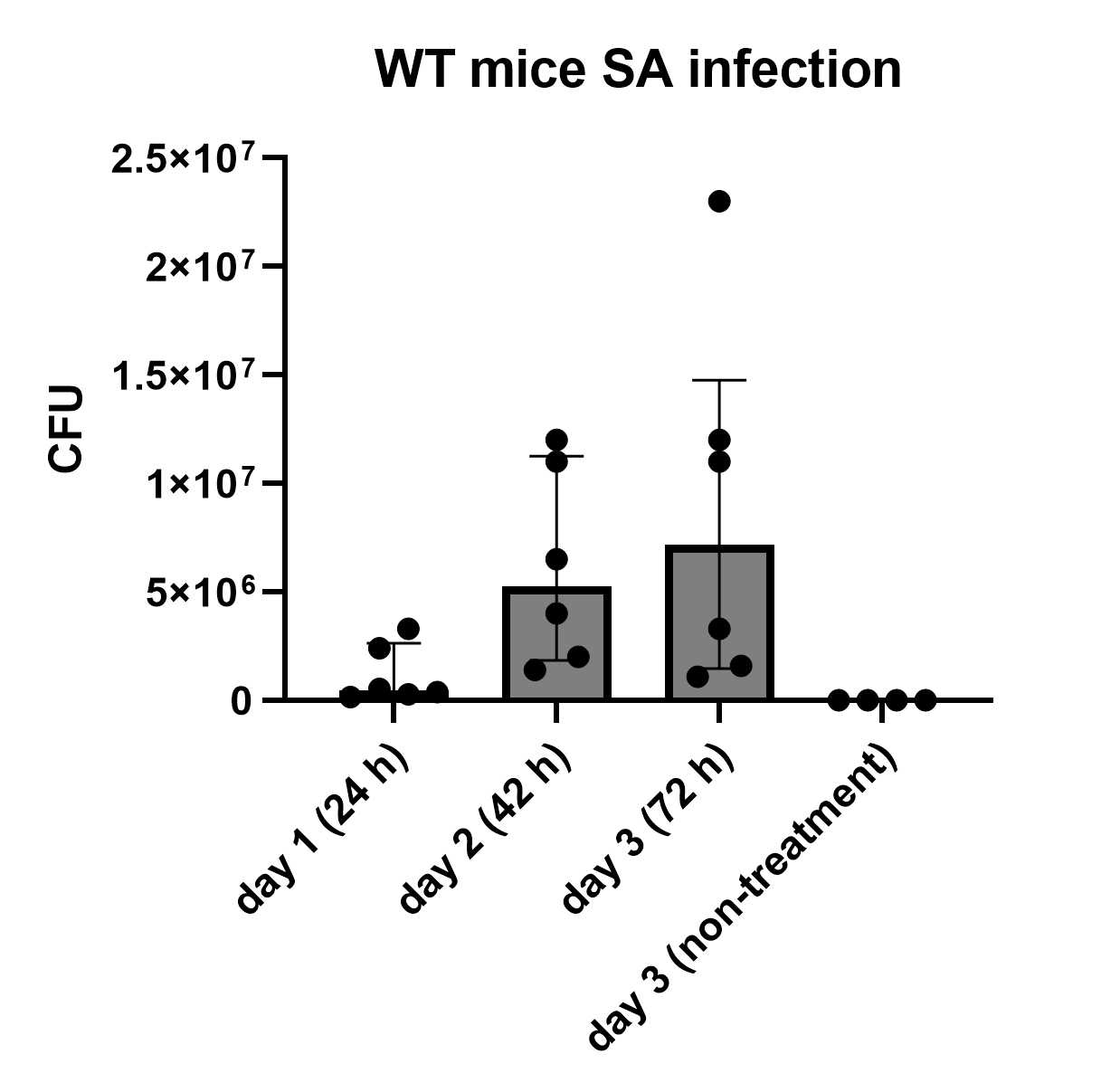

Supplement: S1 File — (ZIP) [file pone.0339341.s006.zip › data set/S2 fig_WT mice SA infection.png]

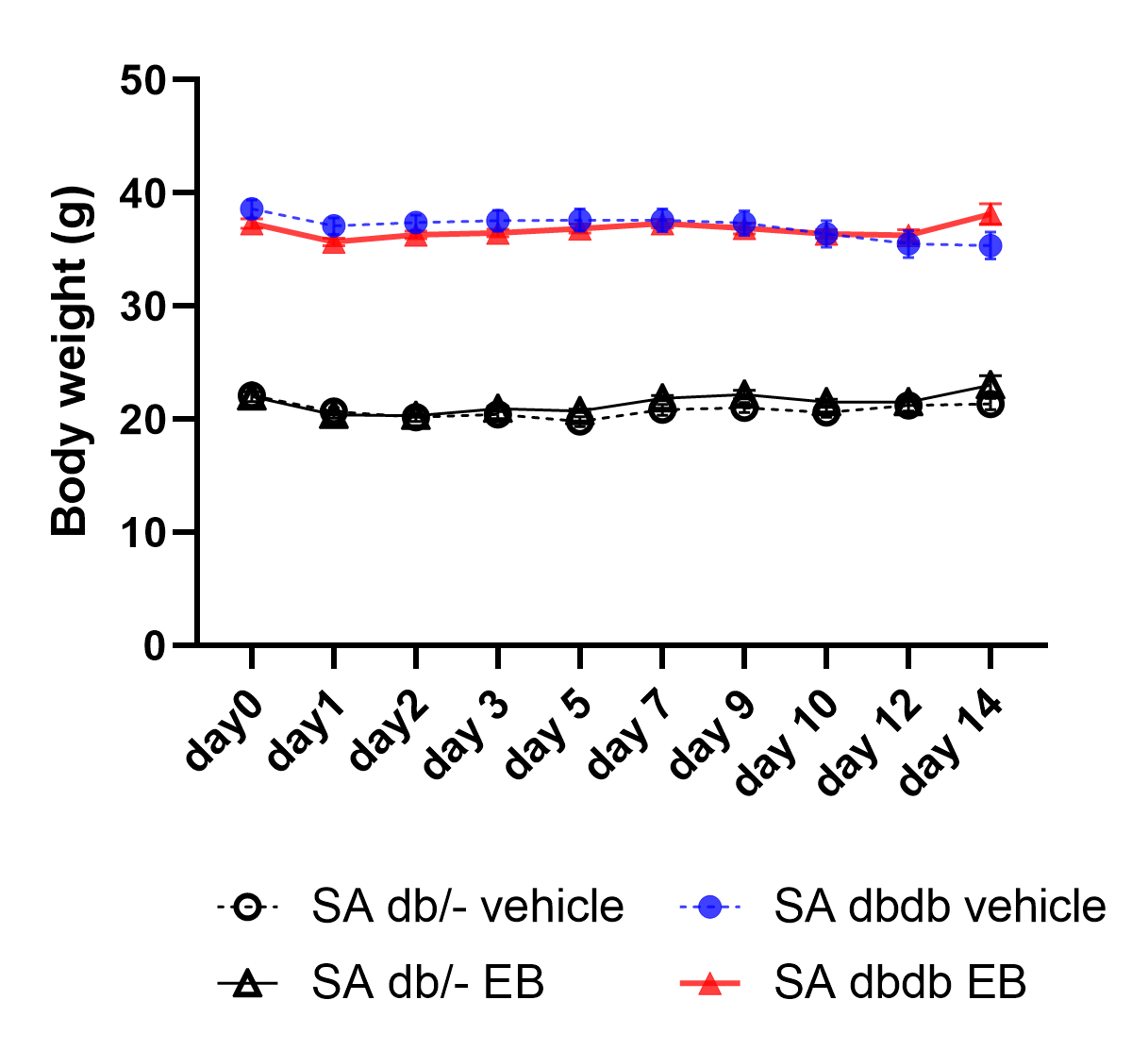

Supplement: S1 File — (ZIP) [file pone.0339341.s006.zip › data set/S3 fig_body weights.png]

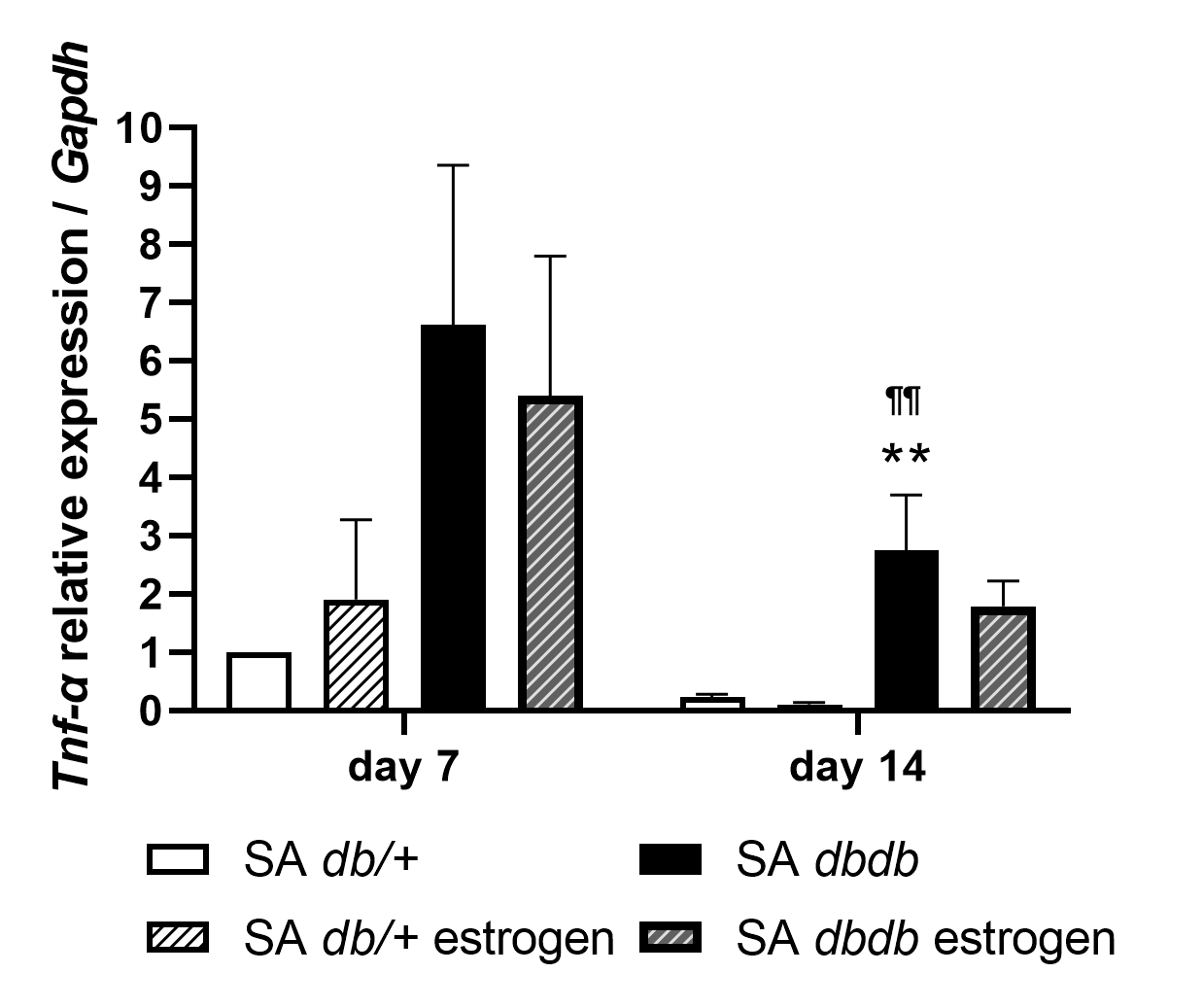

Supplement: S1 File — (ZIP) [file pone.0339341.s006.zip › data set/S5-1 fig_relative tnf a expression SA infection.png]

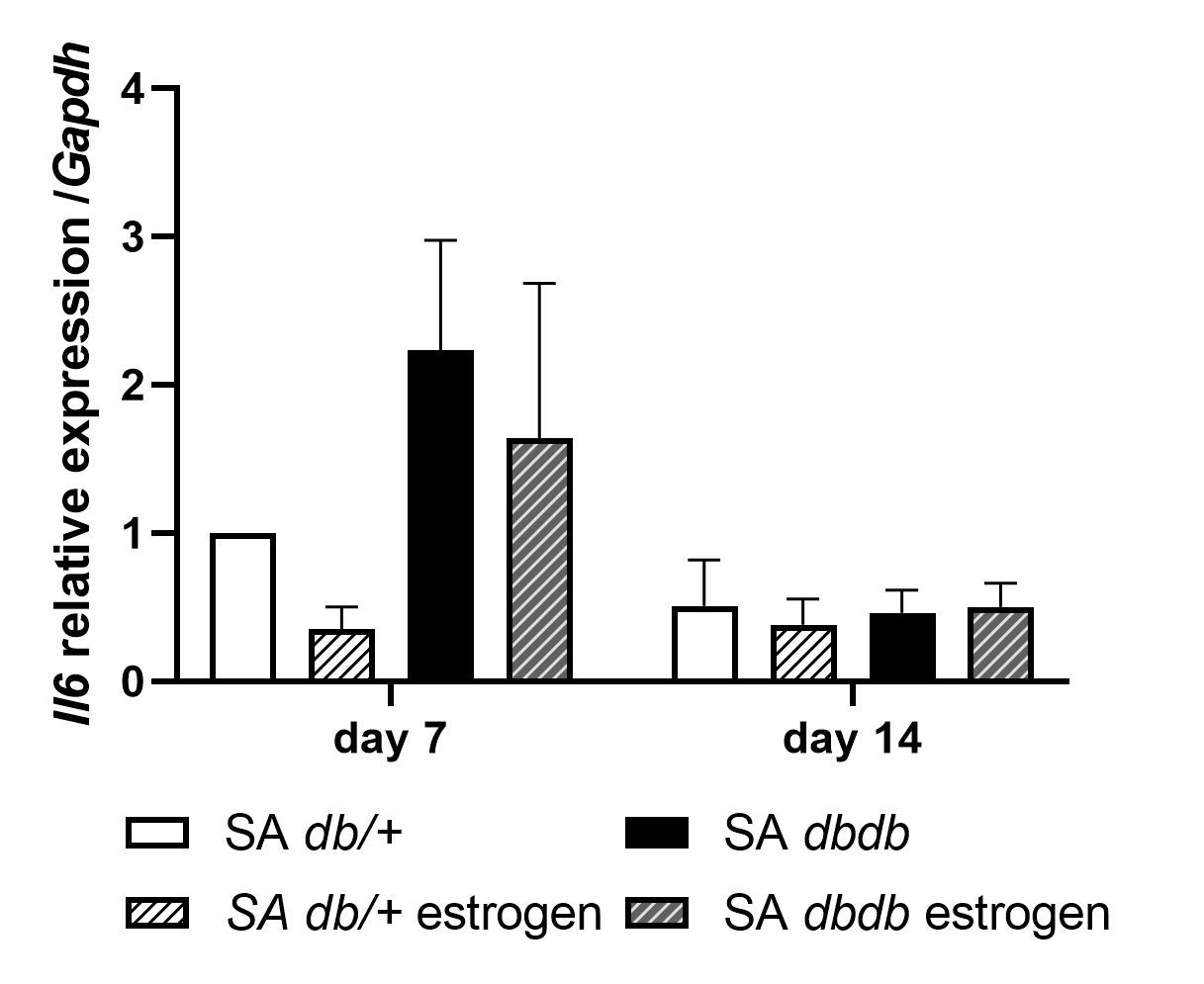

Supplement: S1 File — (ZIP) [file pone.0339341.s006.zip › data set/S5-2 fig_relative il6 expression SA infection.png]

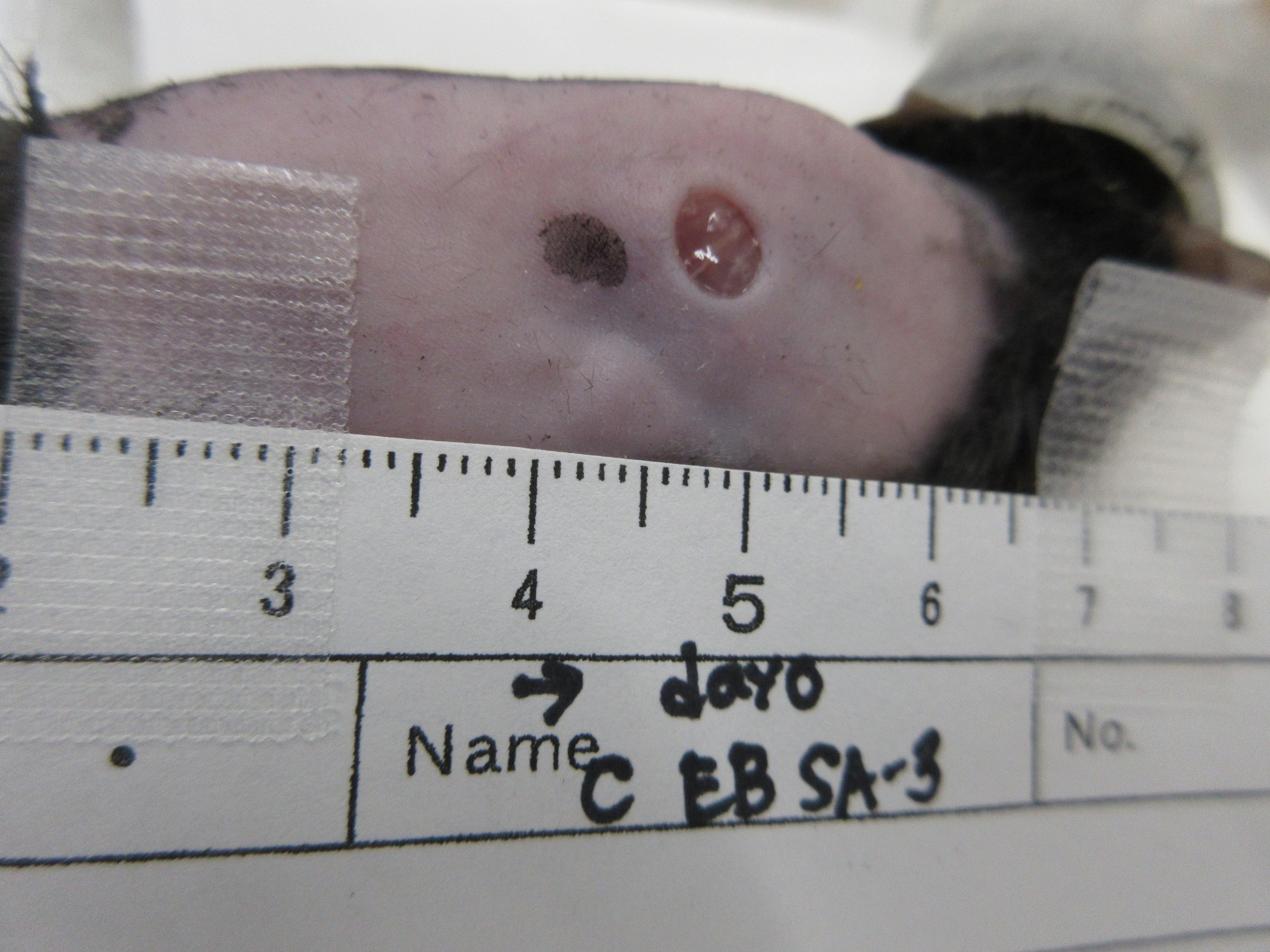

Supplement: S2 File — Fig 2A_wound pictures-1. (ZIP) [file pone.0339341.s007.zip › fig 2A_wound pictures-1/db+ estrogen_day 0.JPG]

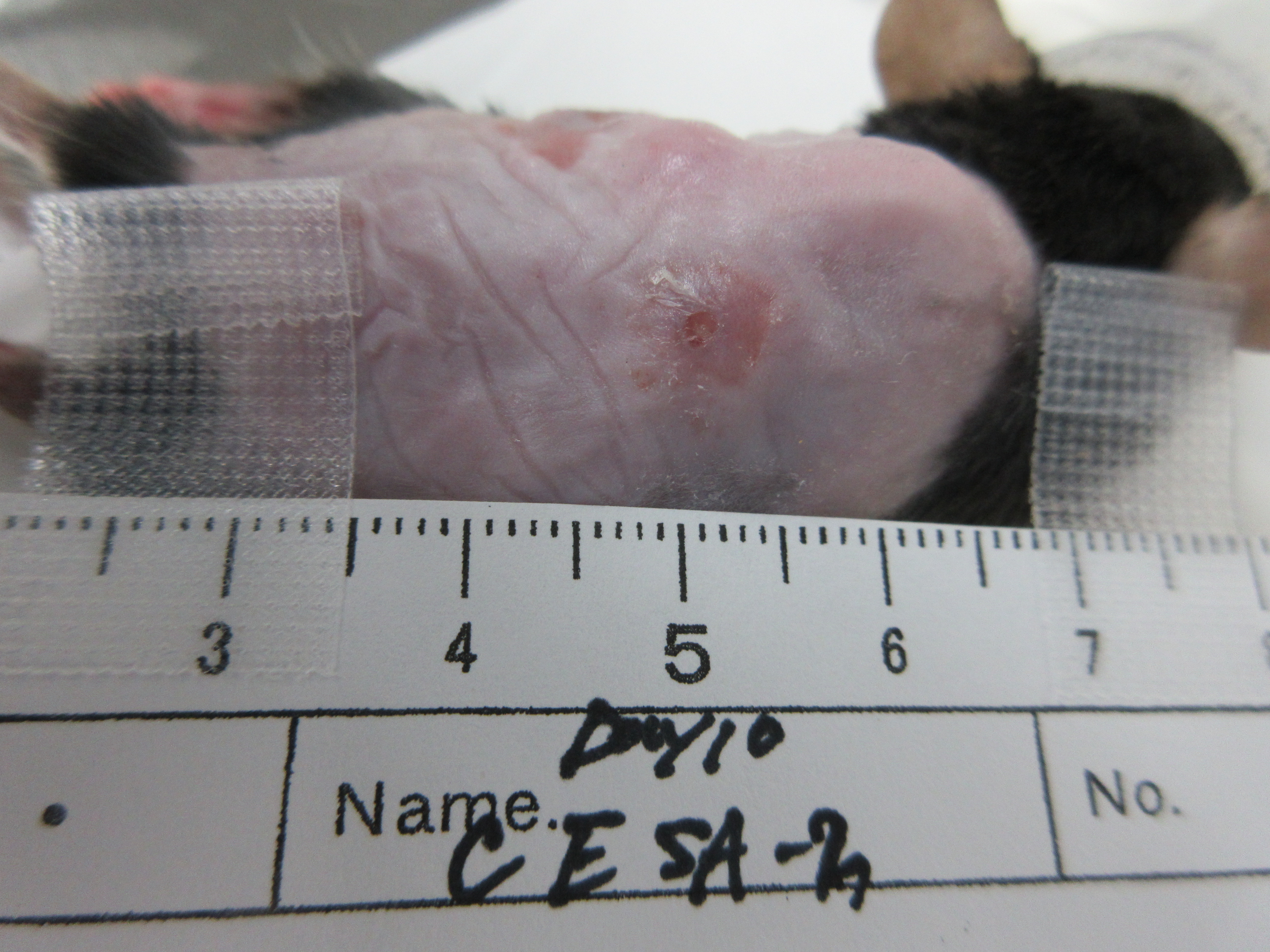

Supplement: S2 File — Fig 2A_wound pictures-1. (ZIP) [file pone.0339341.s007.zip › fig 2A_wound pictures-1/db+ estrogen_day 10.JPG]

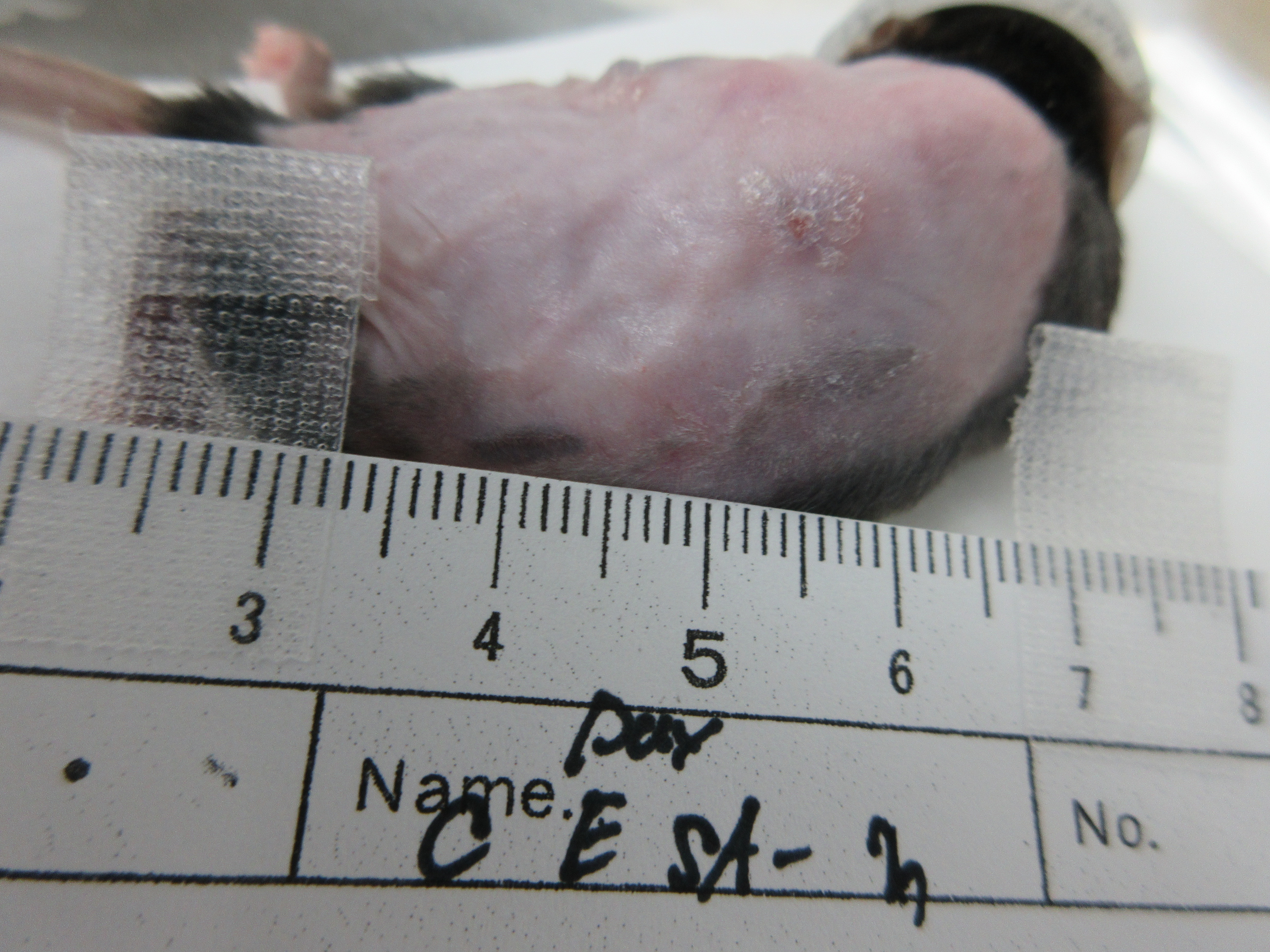

Supplement: S2 File — Fig 2A_wound pictures-1. (ZIP) [file pone.0339341.s007.zip › fig 2A_wound pictures-1/db+ estrogen_day 12.JPG]

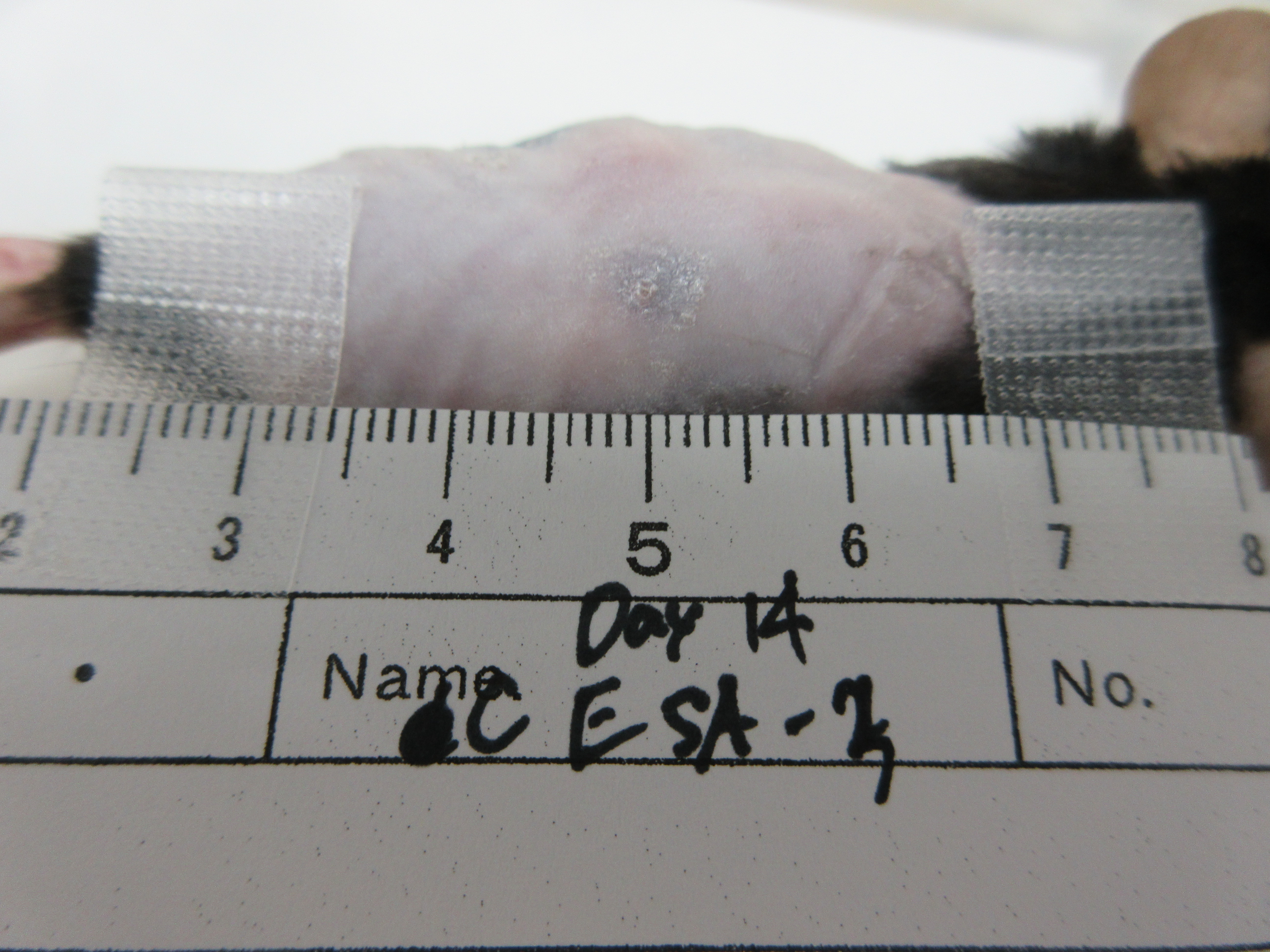

Supplement: S2 File — Fig 2A_wound pictures-1. (ZIP) [file pone.0339341.s007.zip › fig 2A_wound pictures-1/db+ estrogen_day 14.JPG]

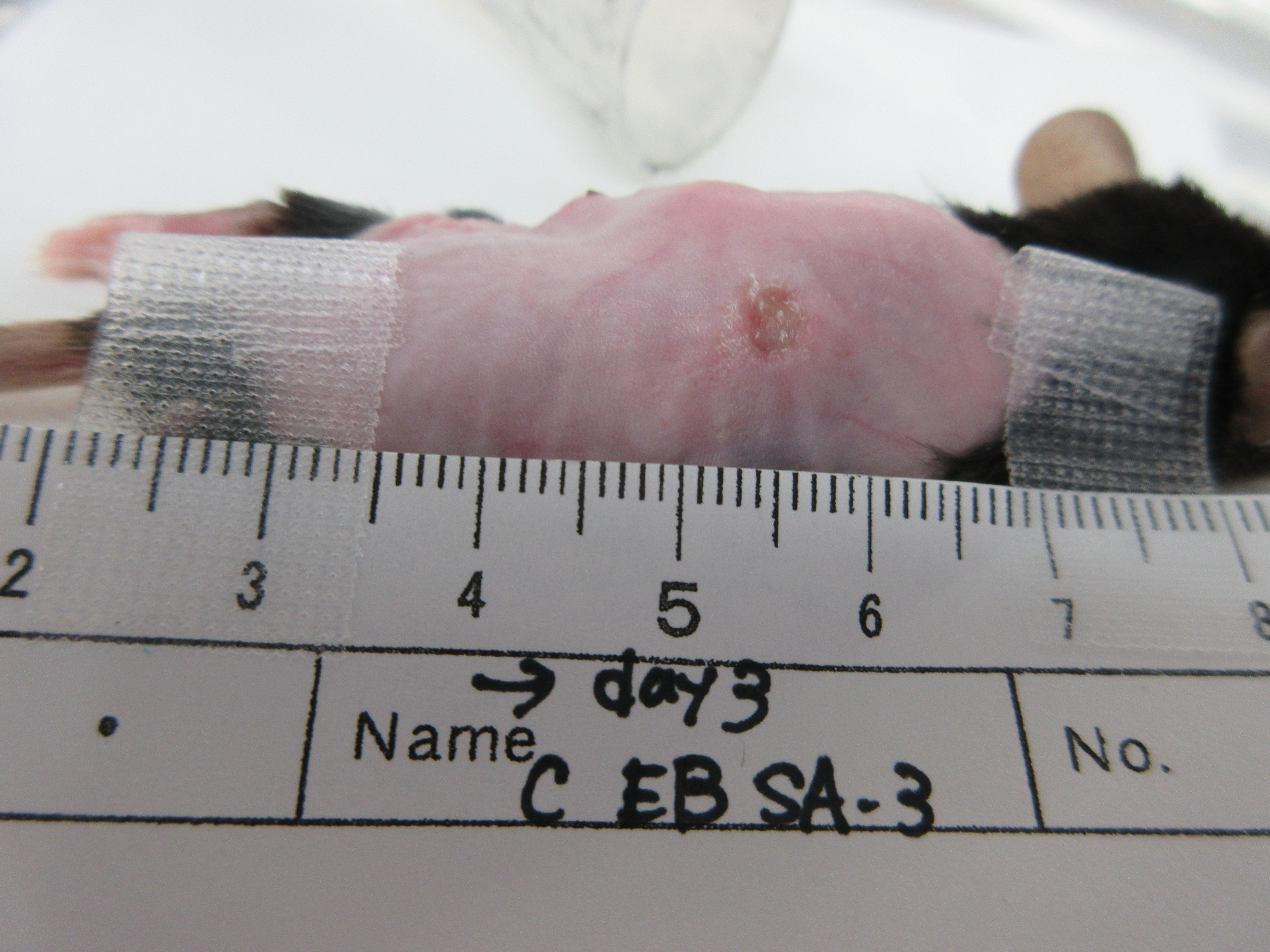

Supplement: S2 File — Fig 2A_wound pictures-1. (ZIP) [file pone.0339341.s007.zip › fig 2A_wound pictures-1/db+ estrogen_day 3.JPG]

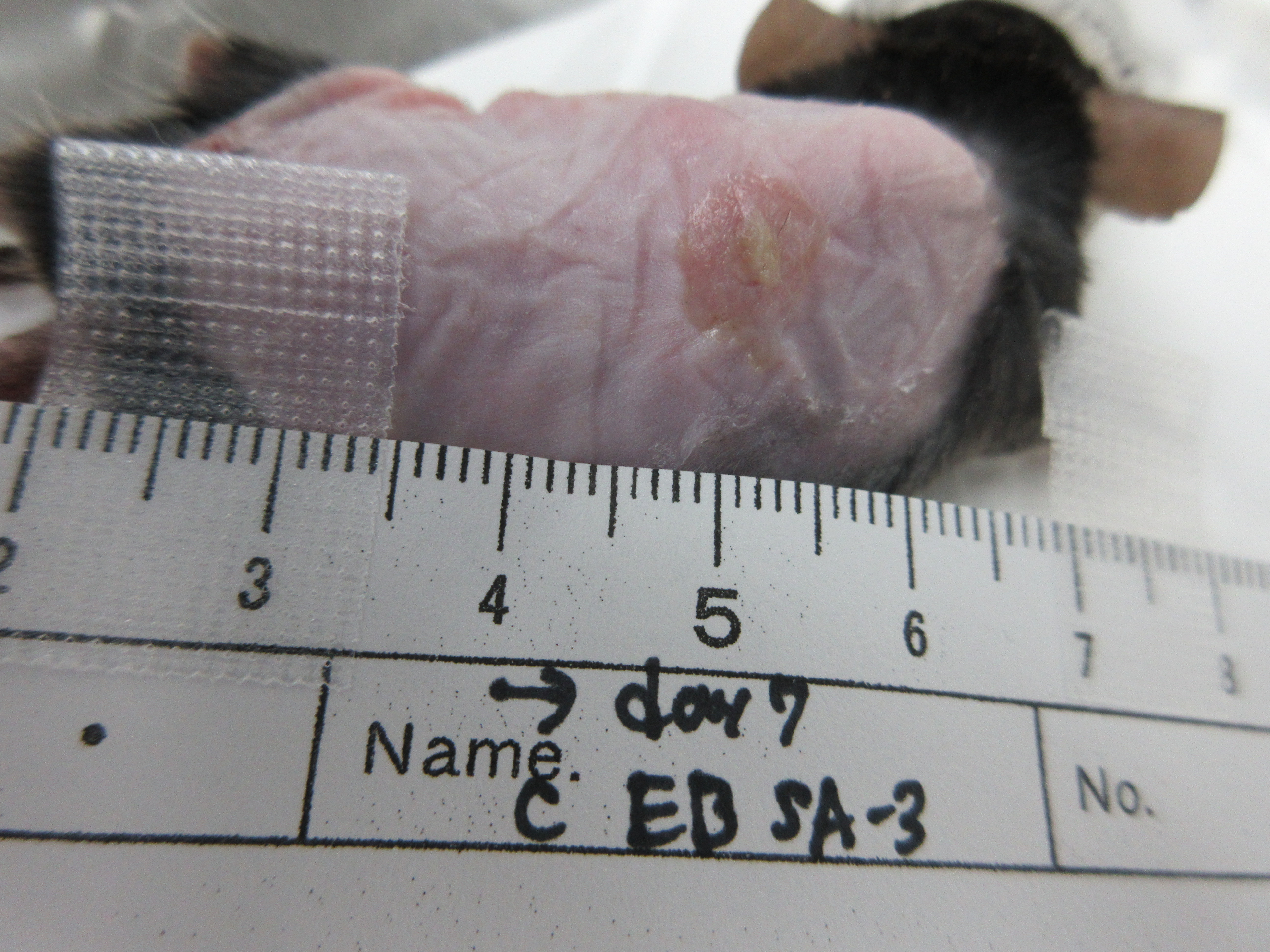

Supplement: S2 File — Fig 2A_wound pictures-1. (ZIP) [file pone.0339341.s007.zip › fig 2A_wound pictures-1/db+ estrogen_day 7.JPG]

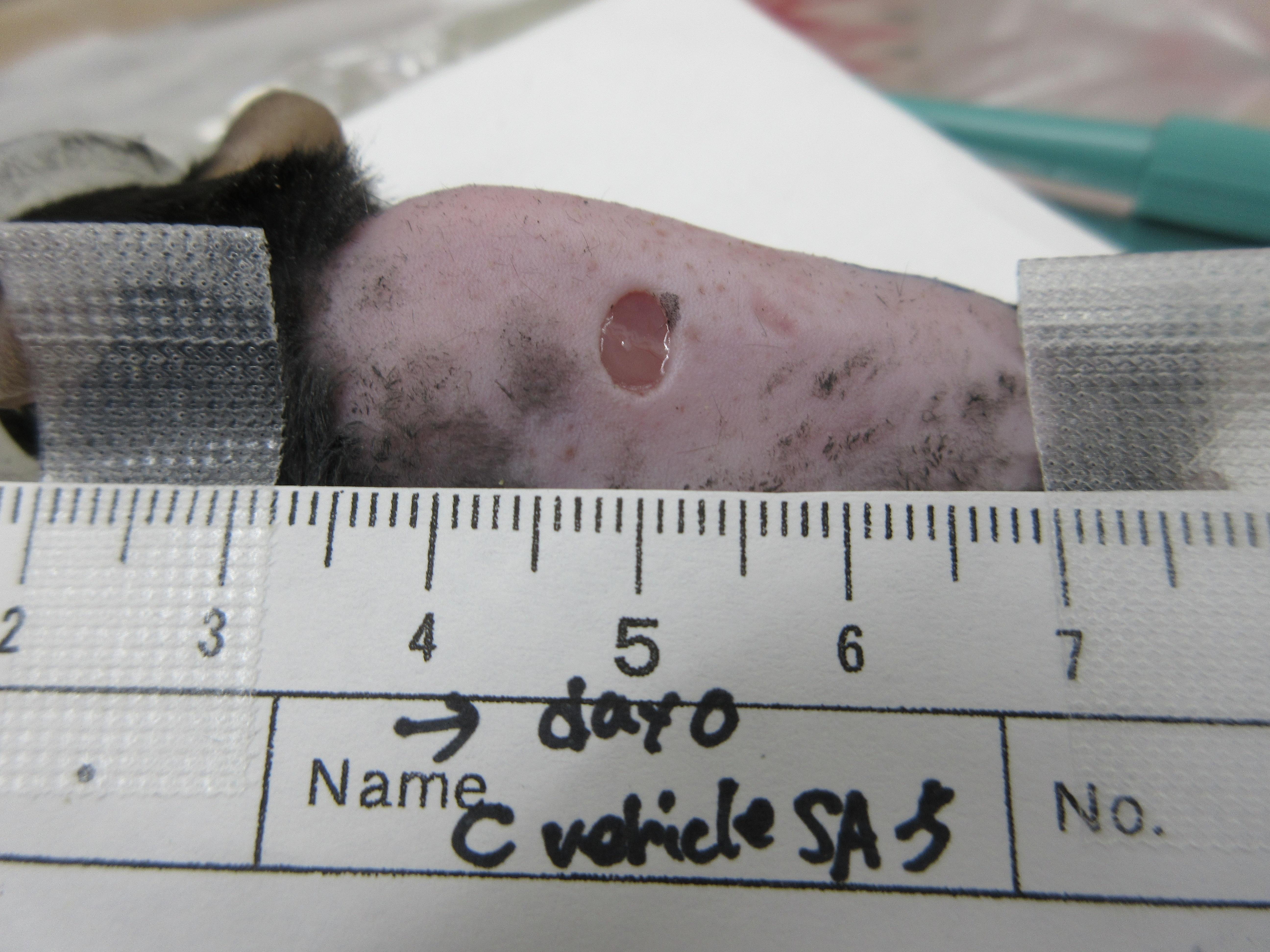

Supplement: S2 File — Fig 2A_wound pictures-1. (ZIP) [file pone.0339341.s007.zip › fig 2A_wound pictures-1/db+_day 0.JPG]

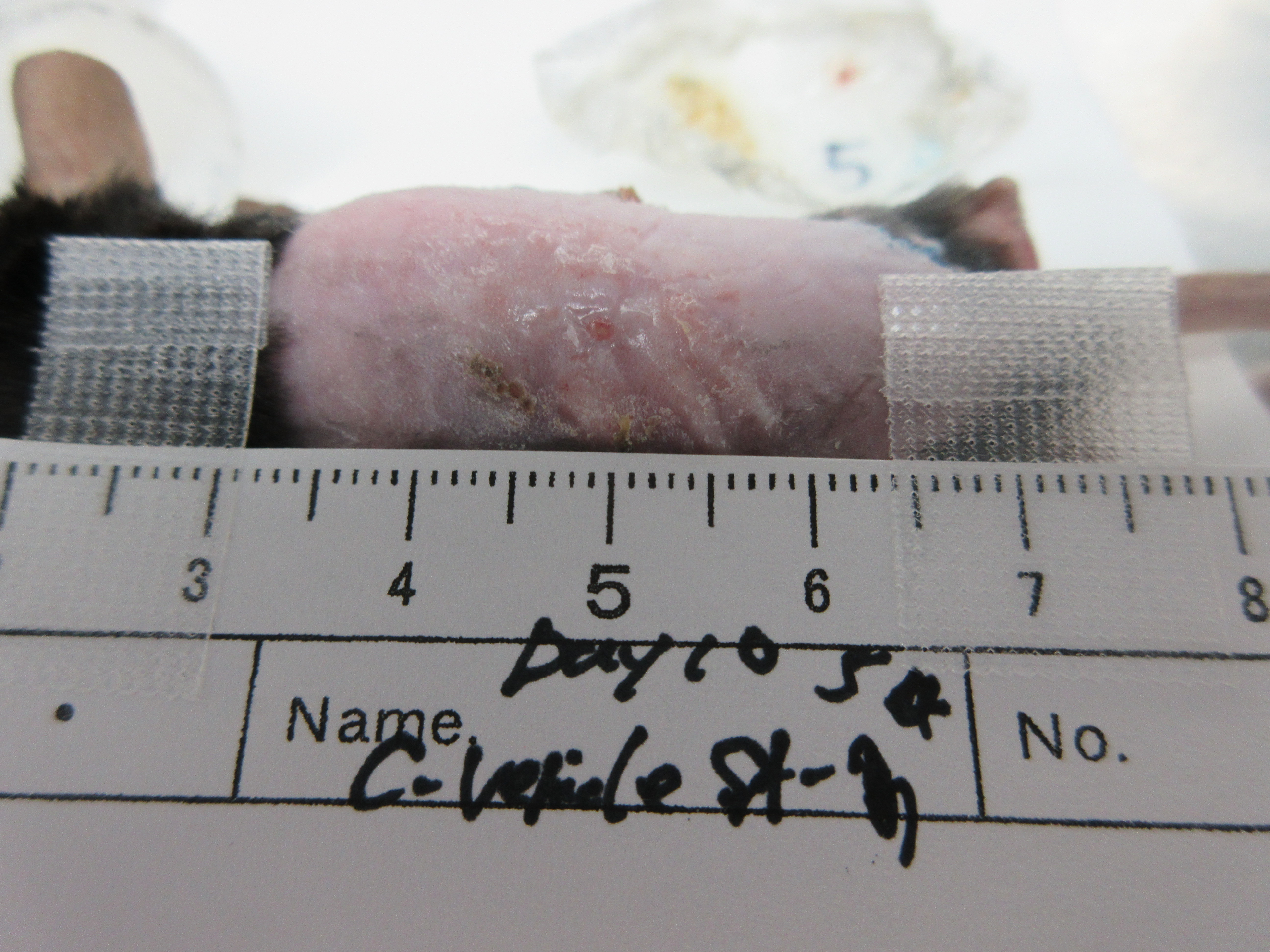

Supplement: S2 File — Fig 2A_wound pictures-1. (ZIP) [file pone.0339341.s007.zip › fig 2A_wound pictures-1/db+_day 10.JPG]

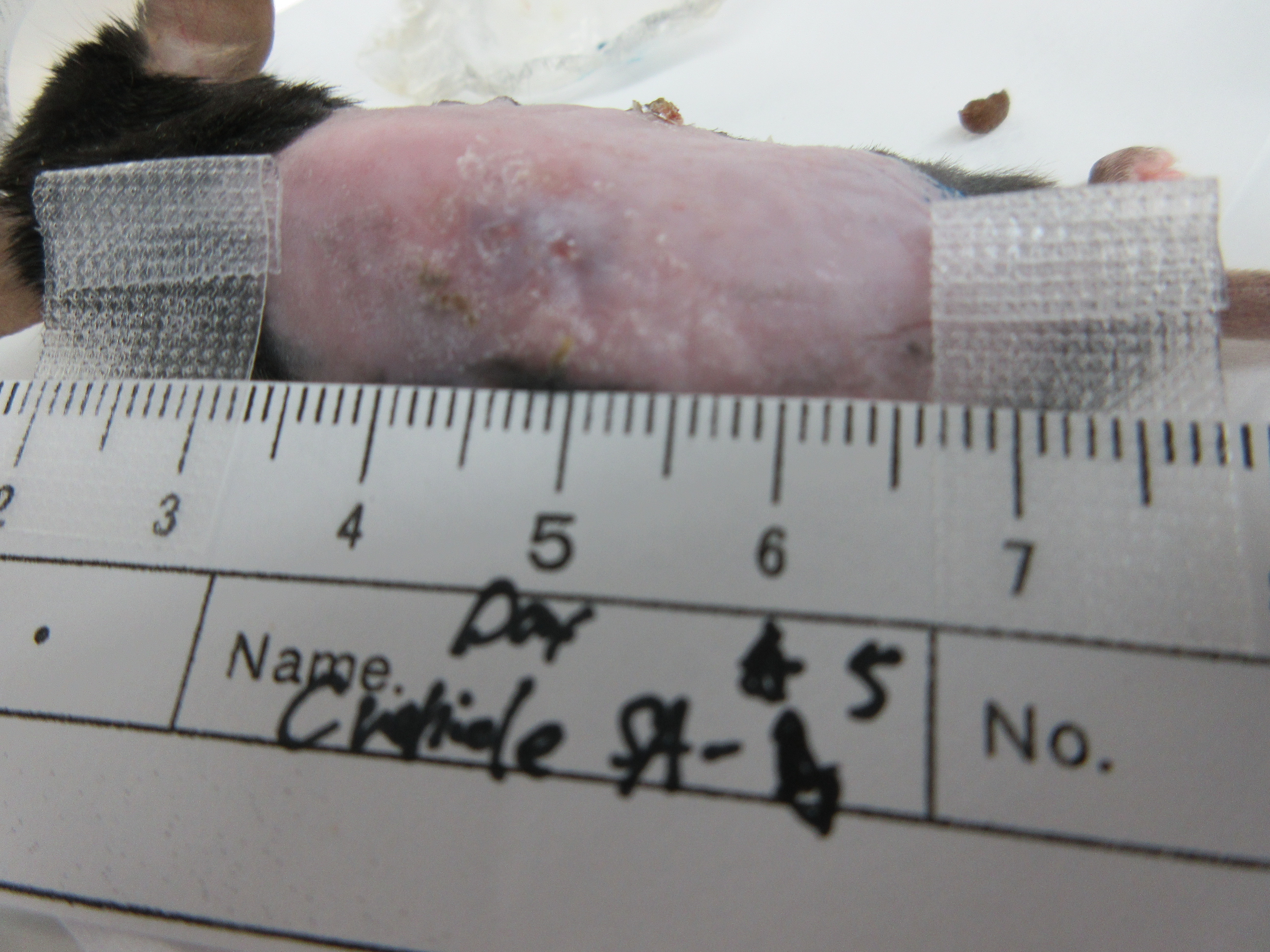

Supplement: S2 File — Fig 2A_wound pictures-1. (ZIP) [file pone.0339341.s007.zip › fig 2A_wound pictures-1/db+_day 12.JPG]

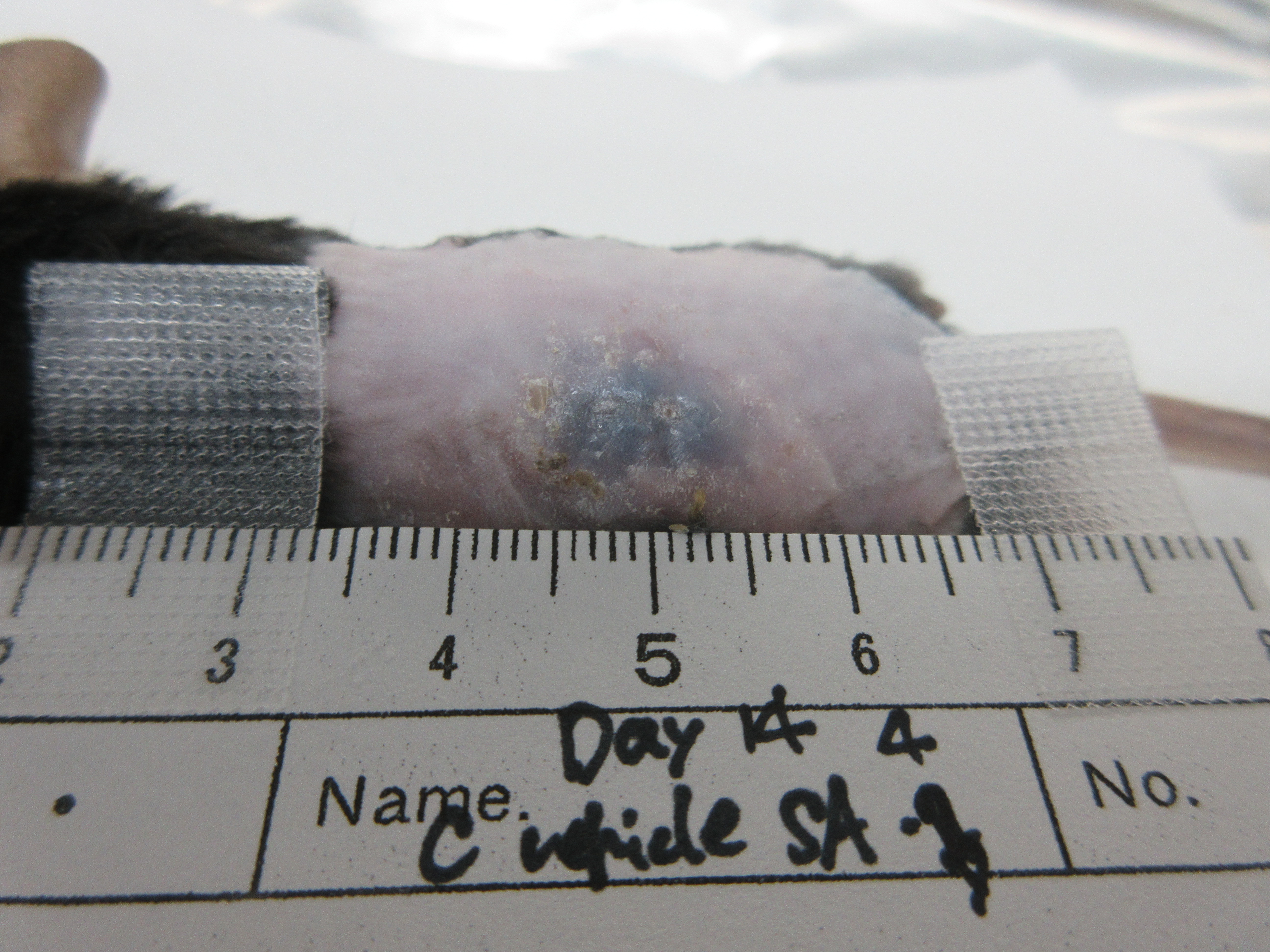

Supplement: S2 File — Fig 2A_wound pictures-1. (ZIP) [file pone.0339341.s007.zip › fig 2A_wound pictures-1/db+_day 14.JPG]

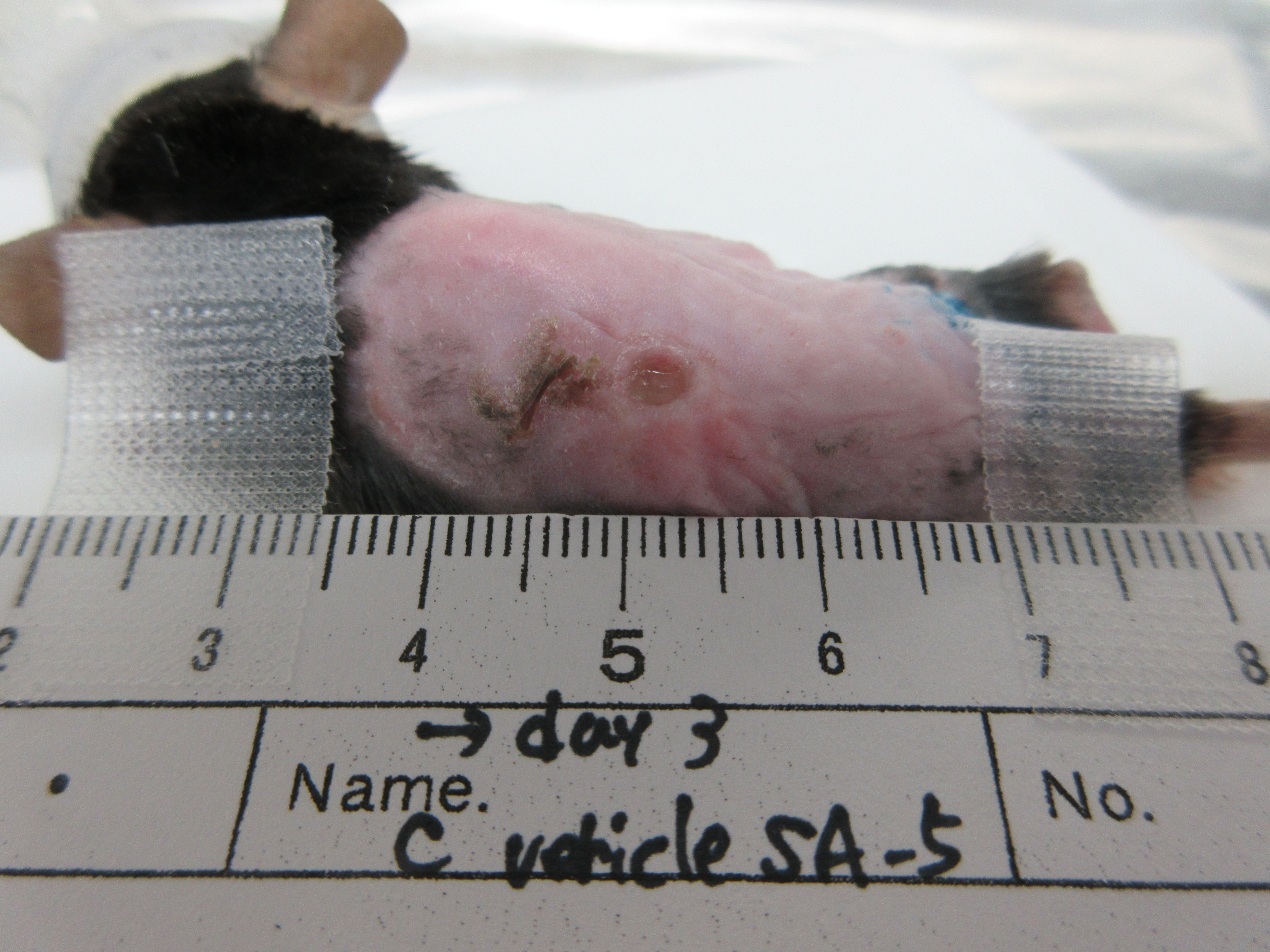

Supplement: S2 File — Fig 2A_wound pictures-1. (ZIP) [file pone.0339341.s007.zip › fig 2A_wound pictures-1/db+_day 3.JPG]

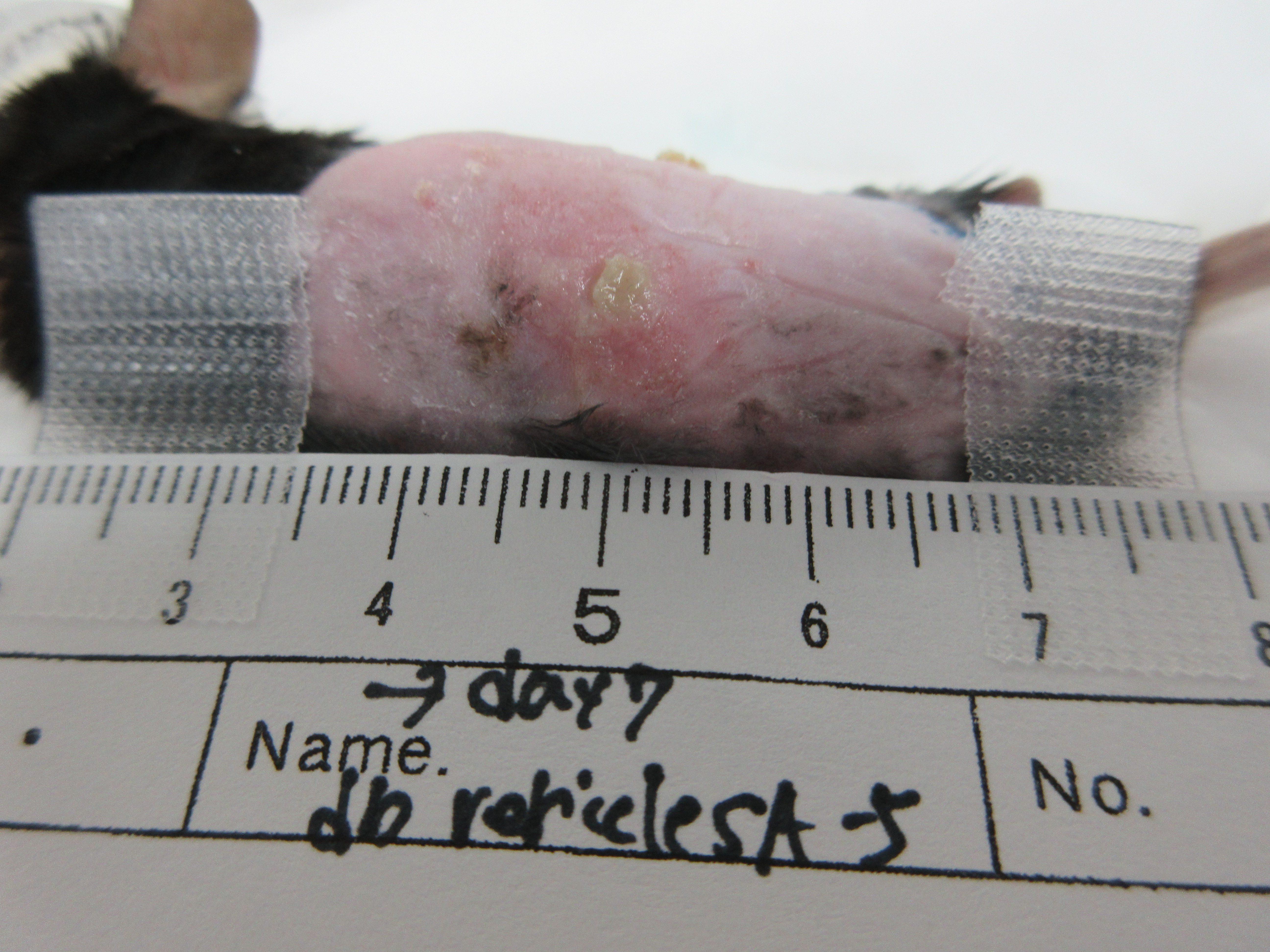

Supplement: S2 File — Fig 2A_wound pictures-1. (ZIP) [file pone.0339341.s007.zip › fig 2A_wound pictures-1/db+_day 7.JPG]

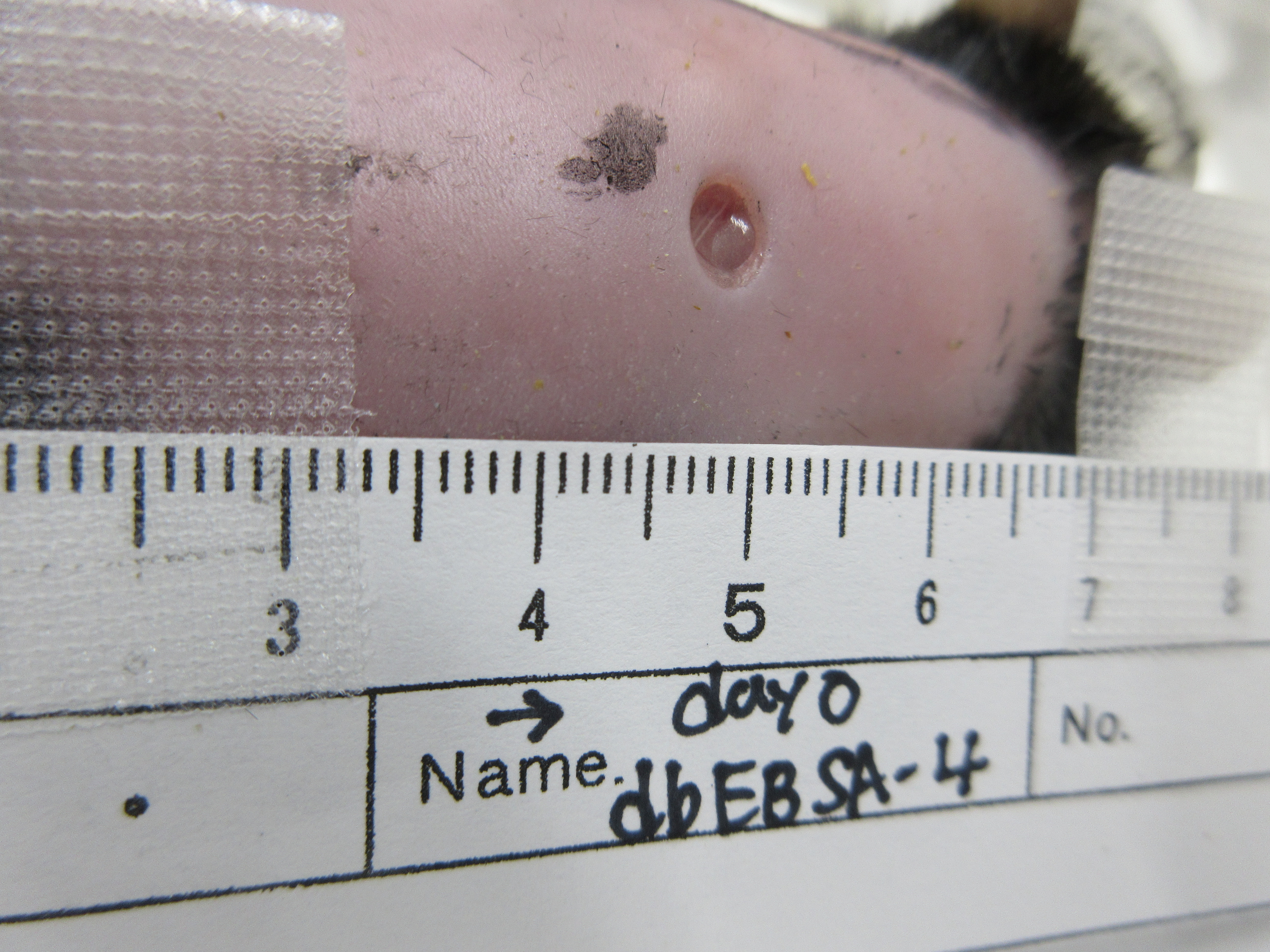

Supplement: S3 File — Fig 2A_wound pictures-2. (ZIP) [file pone.0339341.s008.zip › fig 2A_wound pictures-2/dbdb estrogen_day 0.JPG]

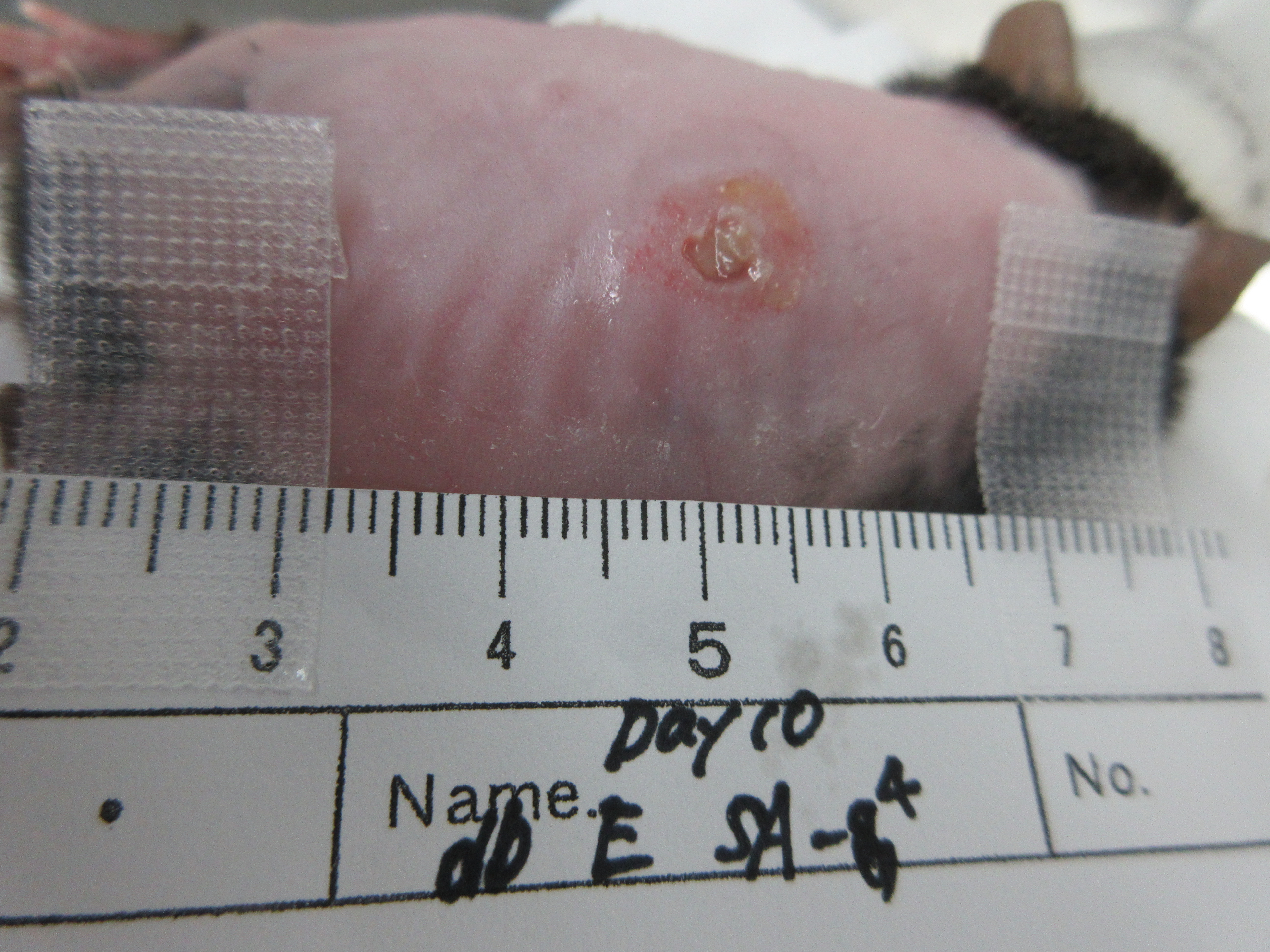

Supplement: S3 File — Fig 2A_wound pictures-2. (ZIP) [file pone.0339341.s008.zip › fig 2A_wound pictures-2/dbdb estrogen_day 10.JPG]

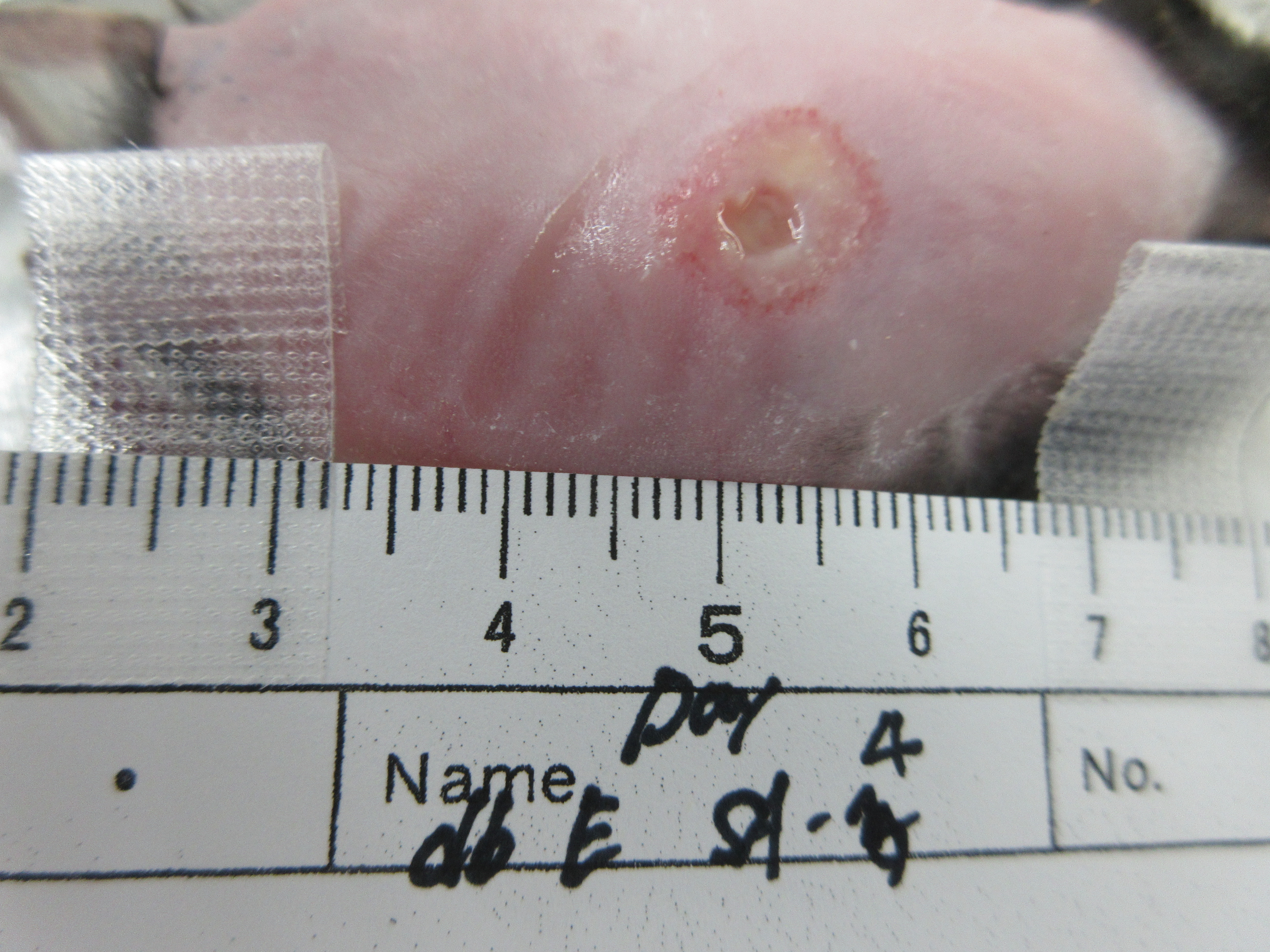

Supplement: S3 File — Fig 2A_wound pictures-2. (ZIP) [file pone.0339341.s008.zip › fig 2A_wound pictures-2/dbdb estrogen_day 12.JPG]

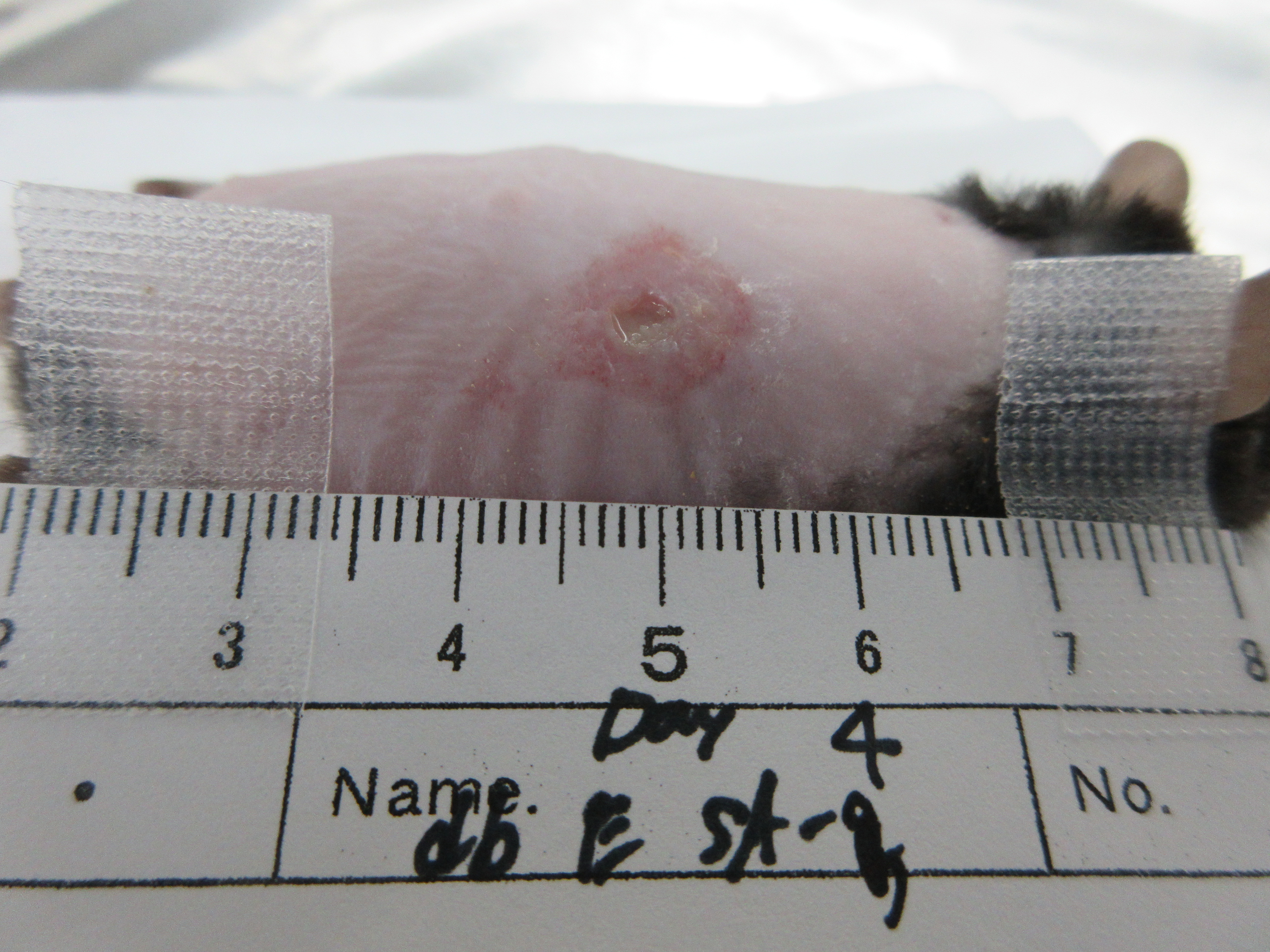

Supplement: S3 File — Fig 2A_wound pictures-2. (ZIP) [file pone.0339341.s008.zip › fig 2A_wound pictures-2/dbdb estrogen_day 14.JPG]

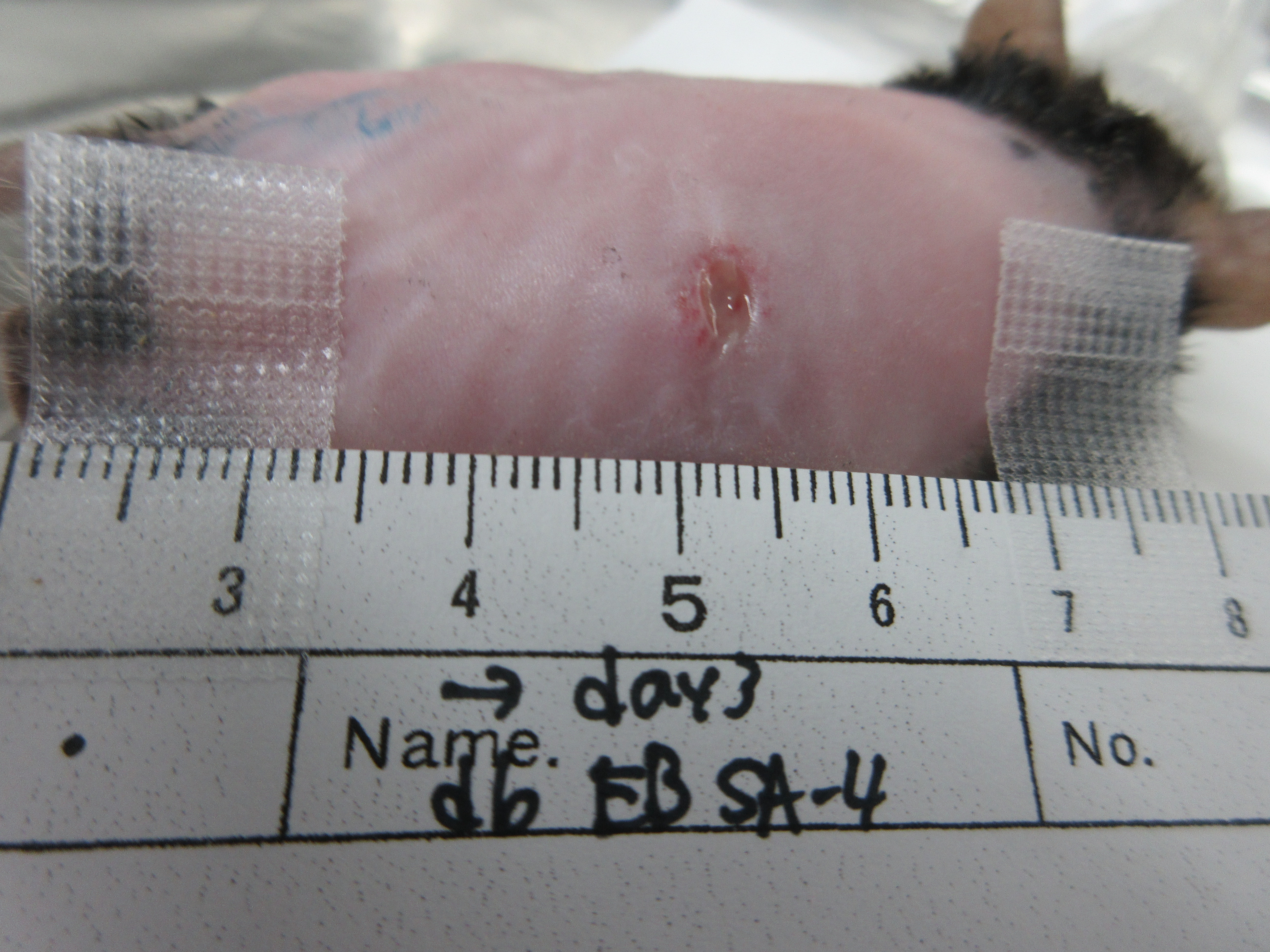

Supplement: S3 File — Fig 2A_wound pictures-2. (ZIP) [file pone.0339341.s008.zip › fig 2A_wound pictures-2/dbdb estrogen_day 3.JPG]

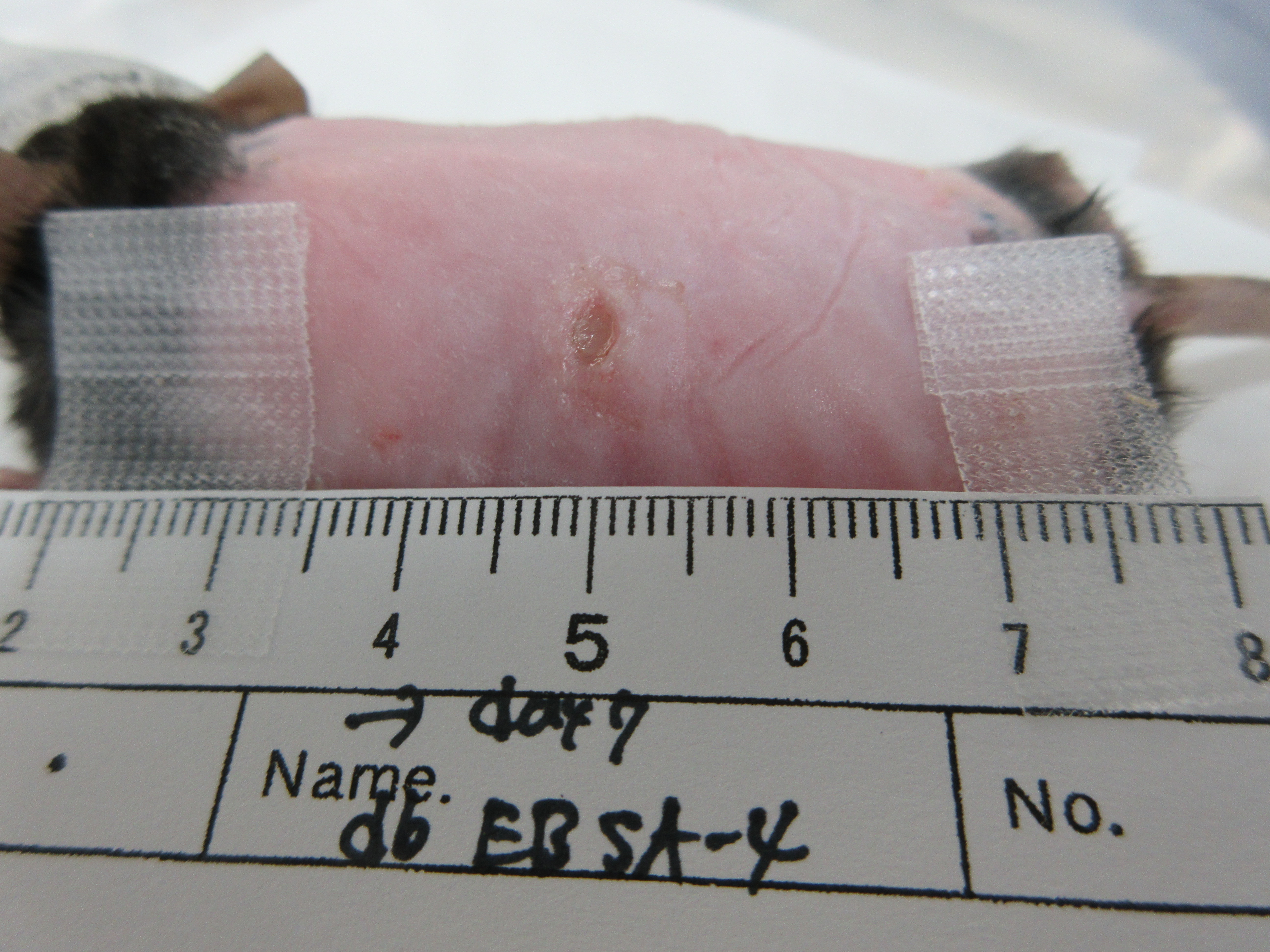

Supplement: S3 File — Fig 2A_wound pictures-2. (ZIP) [file pone.0339341.s008.zip › fig 2A_wound pictures-2/dbdb estrogen_day 7.JPG]

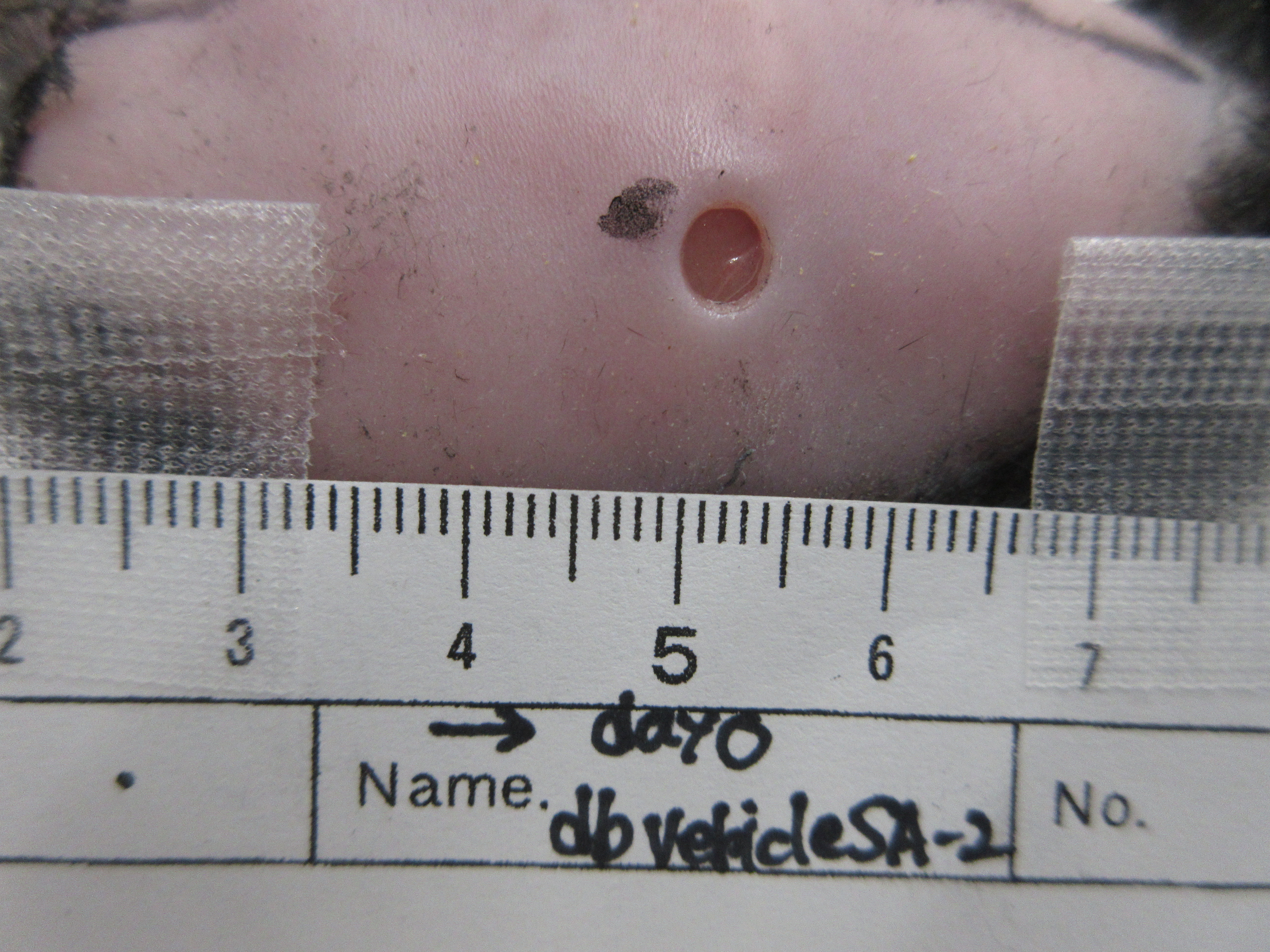

Supplement: S3 File — Fig 2A_wound pictures-2. (ZIP) [file pone.0339341.s008.zip › fig 2A_wound pictures-2/dbdb_day 0.JPG]

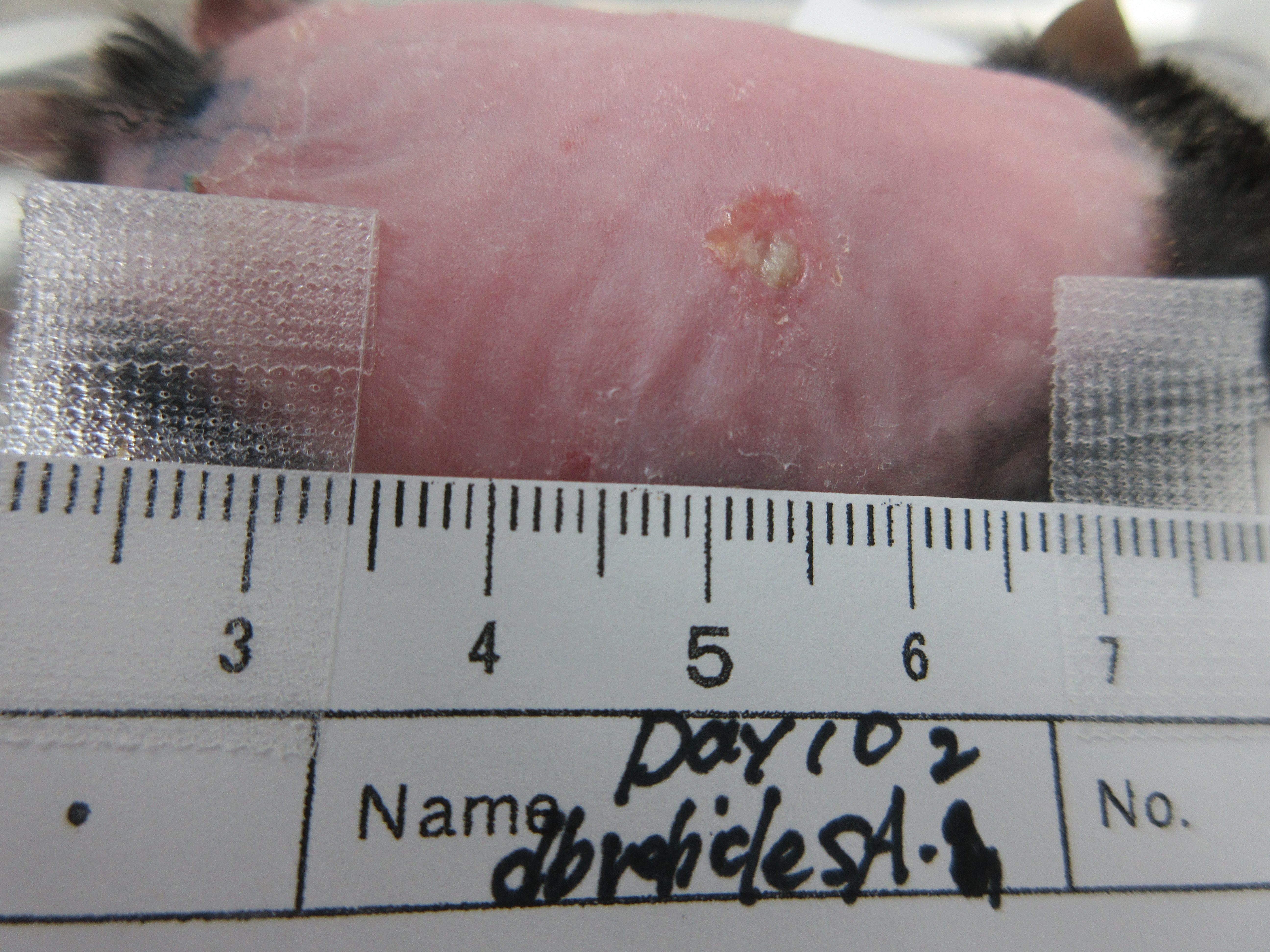

Supplement: S3 File — Fig 2A_wound pictures-2. (ZIP) [file pone.0339341.s008.zip › fig 2A_wound pictures-2/dbdb_day 10.JPG]

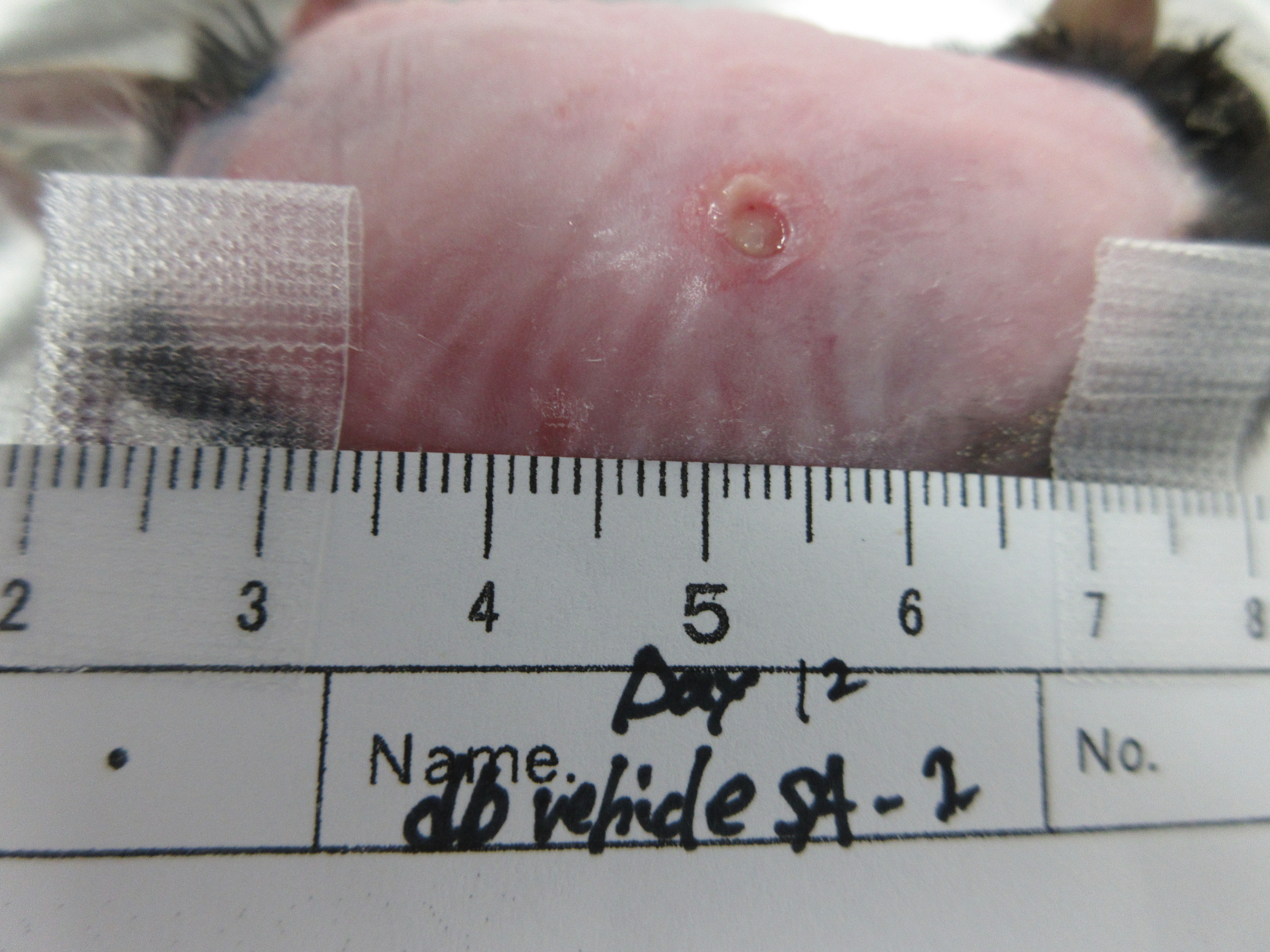

Supplement: S3 File — Fig 2A_wound pictures-2. (ZIP) [file pone.0339341.s008.zip › fig 2A_wound pictures-2/dbdb_day 12.JPG]

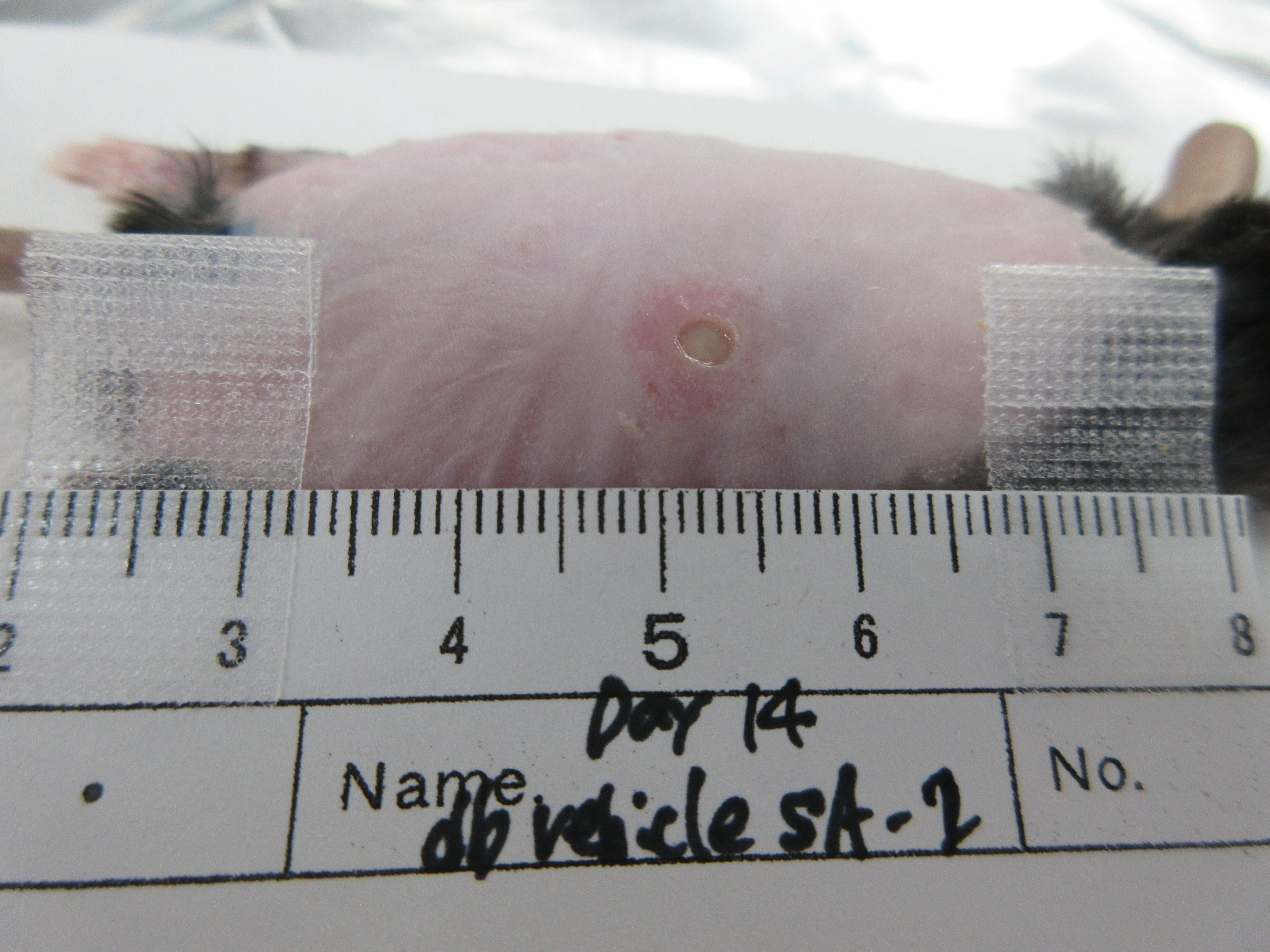

Supplement: S3 File — Fig 2A_wound pictures-2. (ZIP) [file pone.0339341.s008.zip › fig 2A_wound pictures-2/dbdb_day 14.JPG]

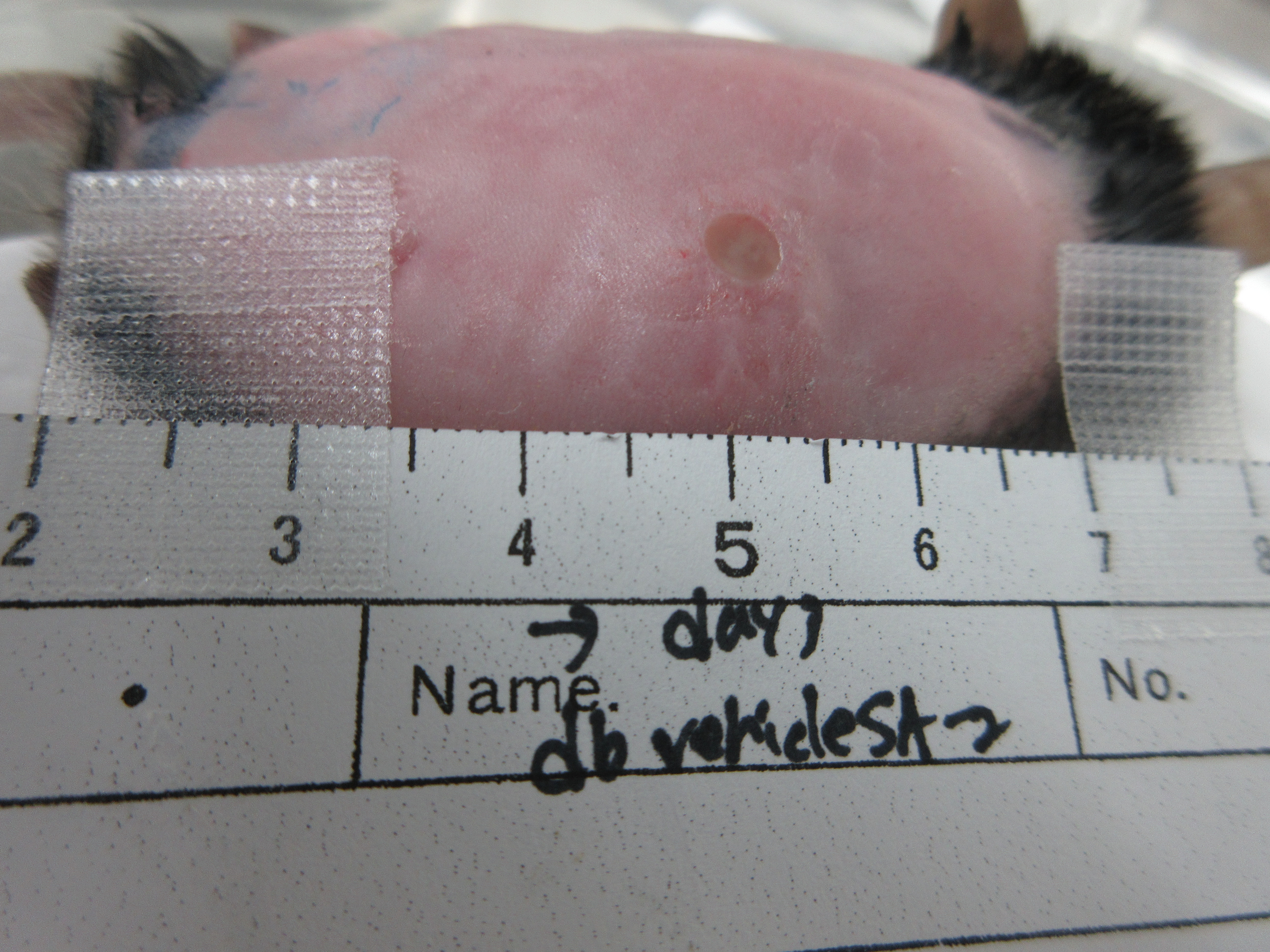

Supplement: S3 File — Fig 2A_wound pictures-2. (ZIP) [file pone.0339341.s008.zip › fig 2A_wound pictures-2/dbdb_day 3.JPG]

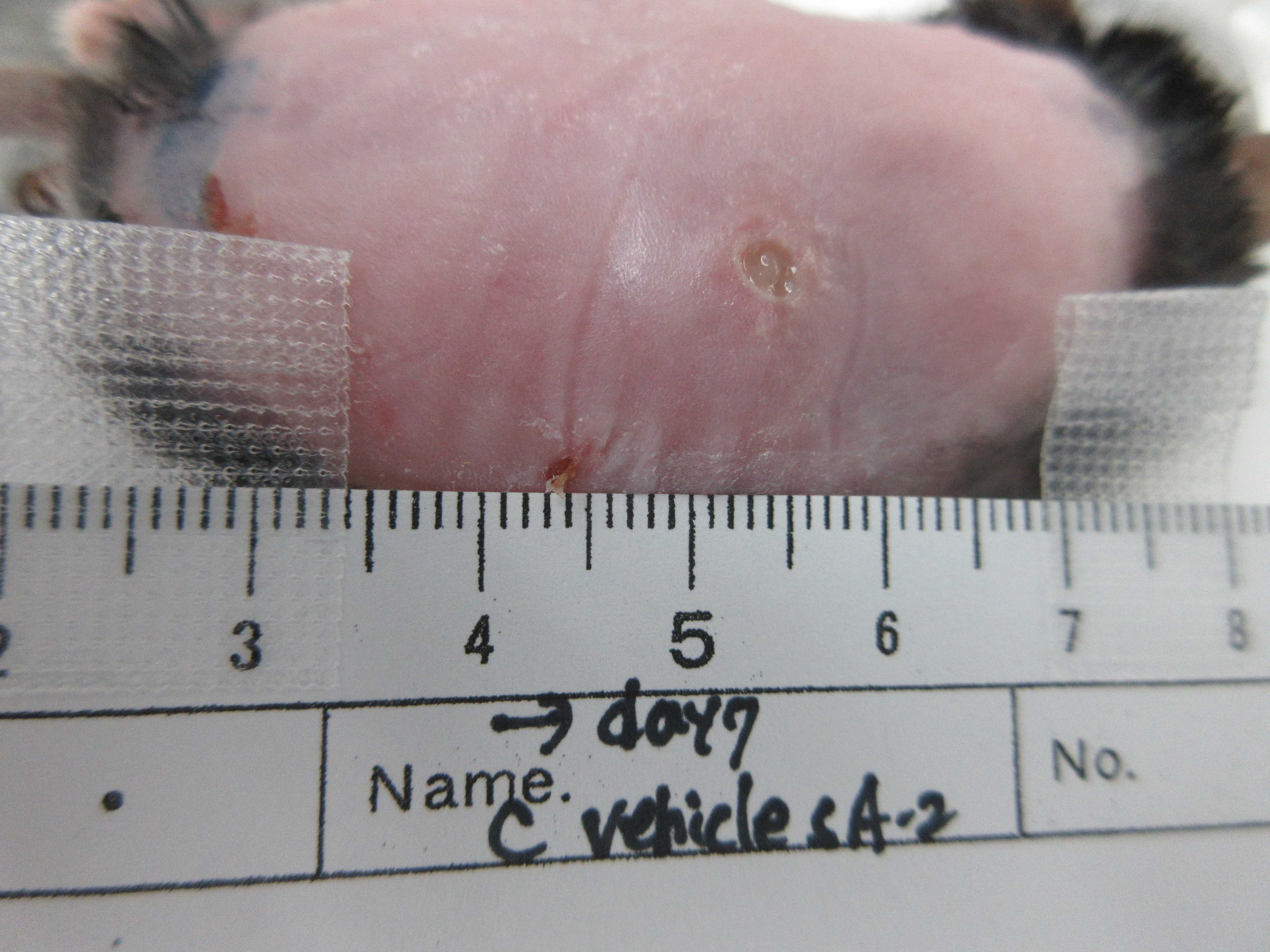

Supplement: S3 File — Fig 2A_wound pictures-2. (ZIP) [file pone.0339341.s008.zip › fig 2A_wound pictures-2/dbdb_day 7.JPG]

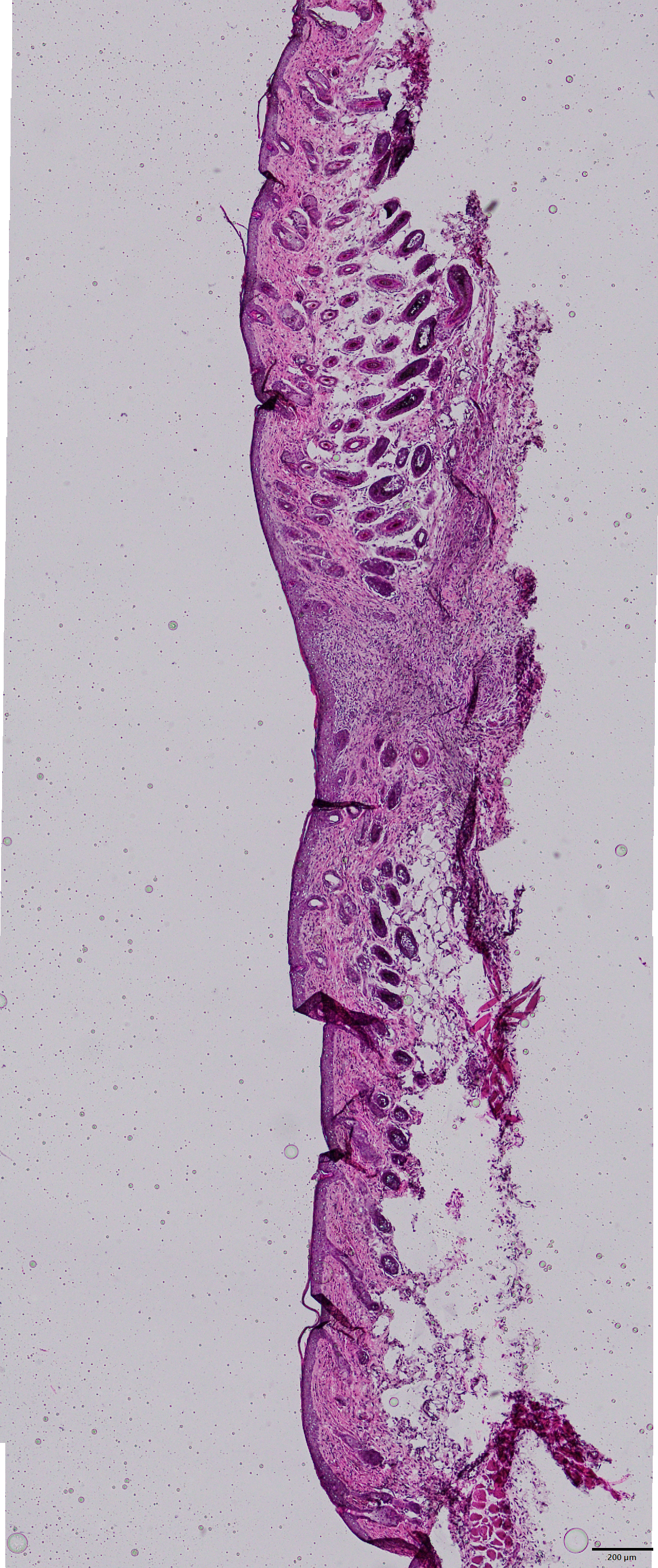

Supplement: S4 File — Fig 3B_wound images-1. (ZIP) [file pone.0339341.s009.zip › fig 3B_wound images-1/db+ estrogen_day 14.tif]

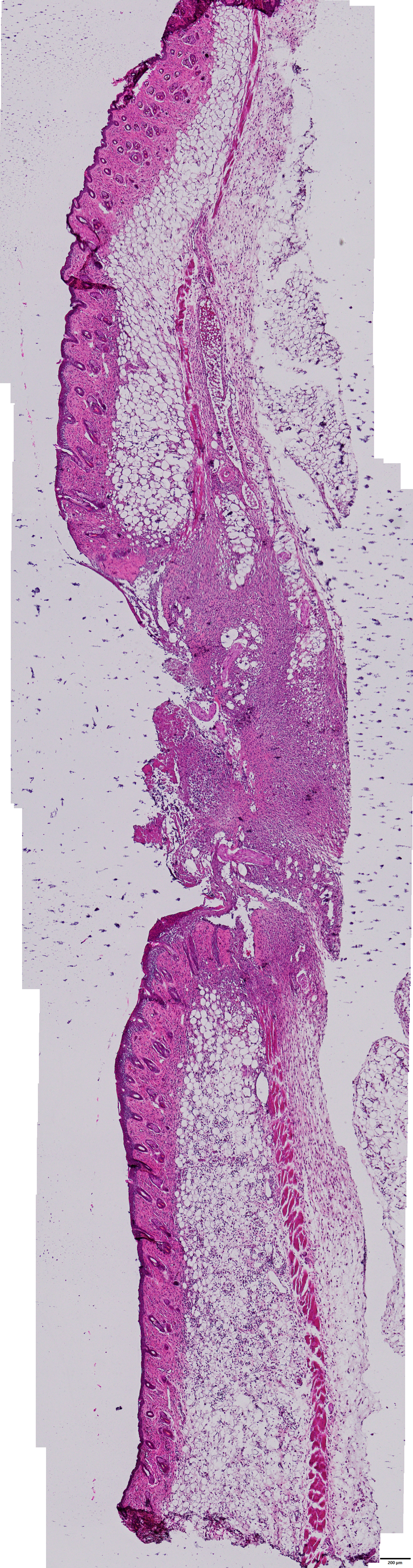

Supplement: S4 File — Fig 3B_wound images-1. (ZIP) [file pone.0339341.s009.zip › fig 3B_wound images-1/db+ estrogen_day 7.tif]

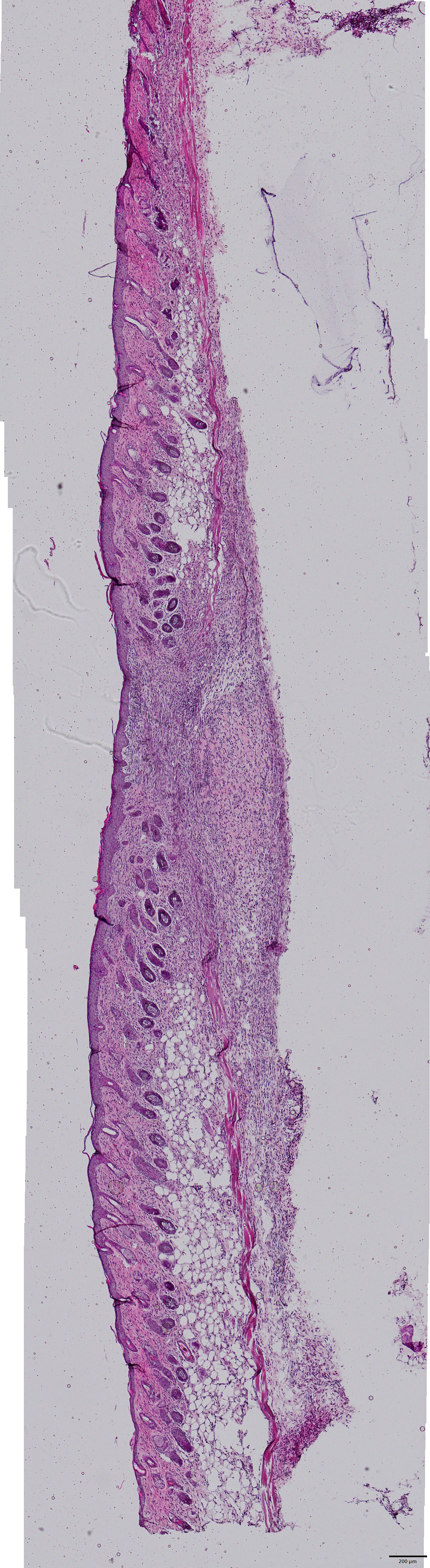

Supplement: S4 File — Fig 3B_wound images-1. (ZIP) [file pone.0339341.s009.zip › fig 3B_wound images-1/db+_day 14.tif]

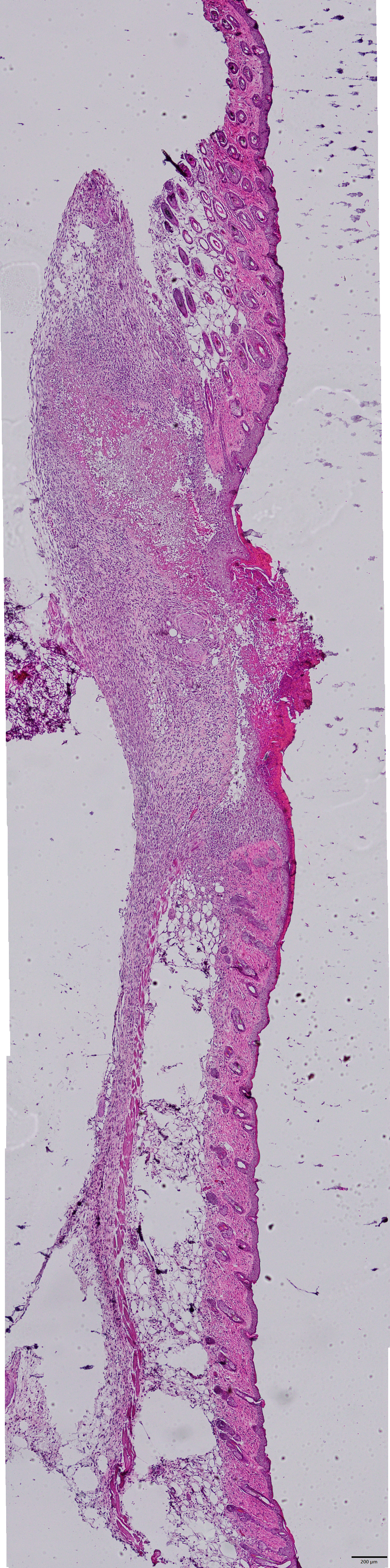

Supplement: S4 File — Fig 3B_wound images-1. (ZIP) [file pone.0339341.s009.zip › fig 3B_wound images-1/db+_day 7.tif]

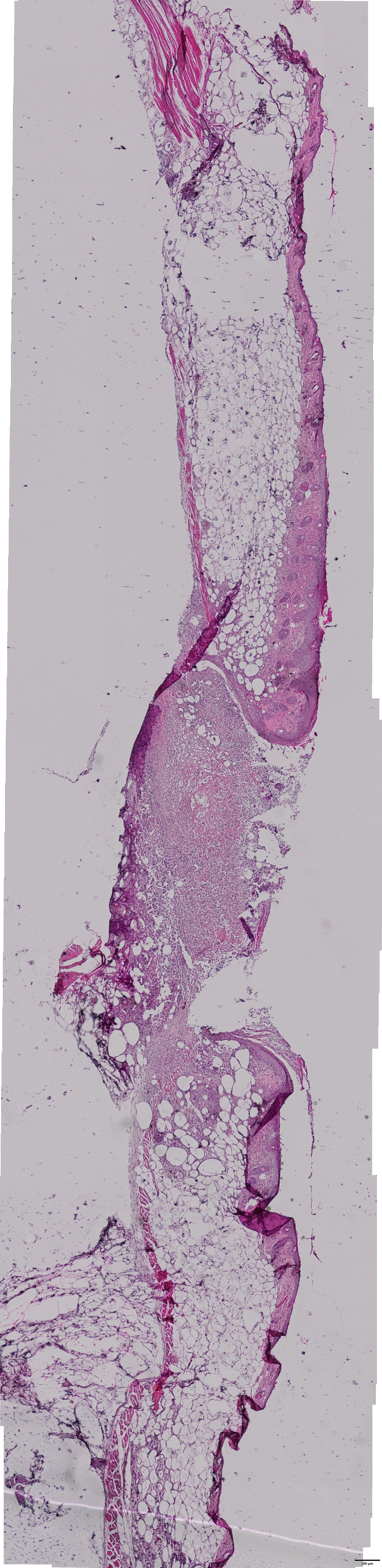

Supplement: S5 File — Fig 3B_wound images-2. (ZIP) [file pone.0339341.s010.zip › fig 3B_wound images-2/dbdb estrogen_day 14.tif]

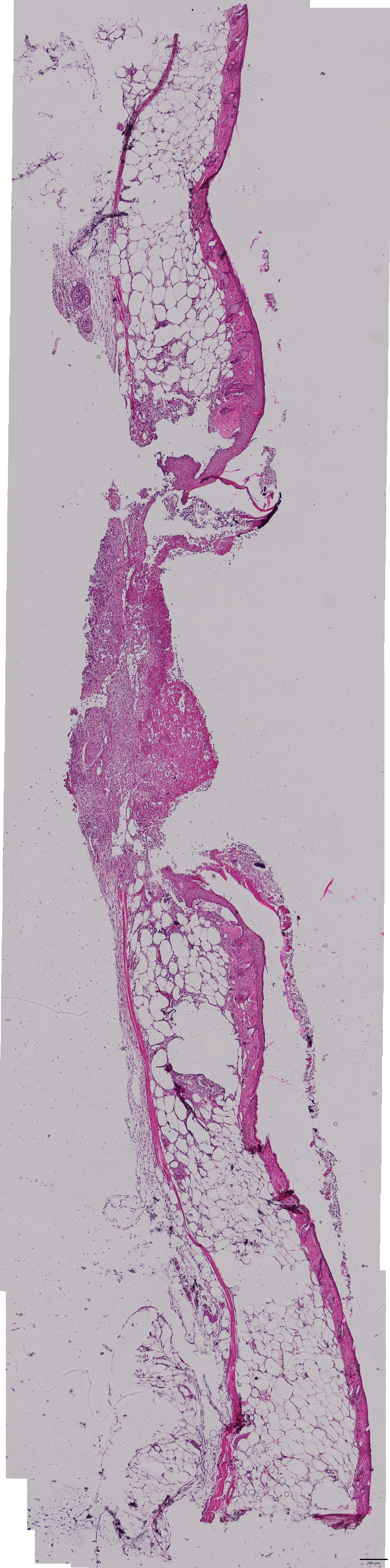

Supplement: S5 File — Fig 3B_wound images-2. (ZIP) [file pone.0339341.s010.zip › fig 3B_wound images-2/dbdb estrogen_day 7.tif]

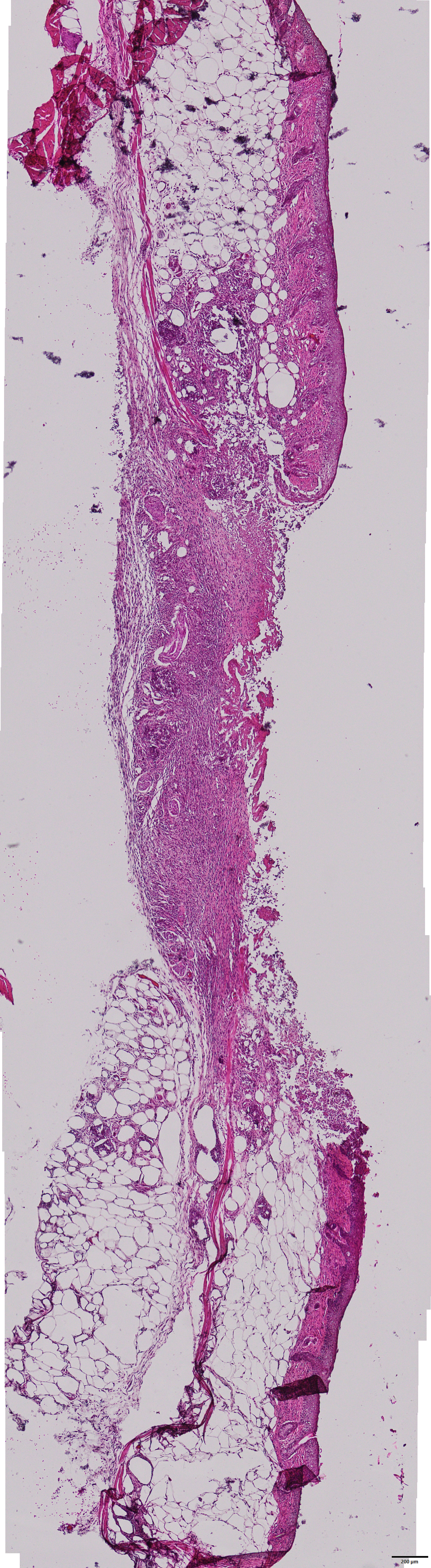

Supplement: S5 File — Fig 3B_wound images-2. (ZIP) [file pone.0339341.s010.zip › fig 3B_wound images-2/dbdb_day 14.tif]

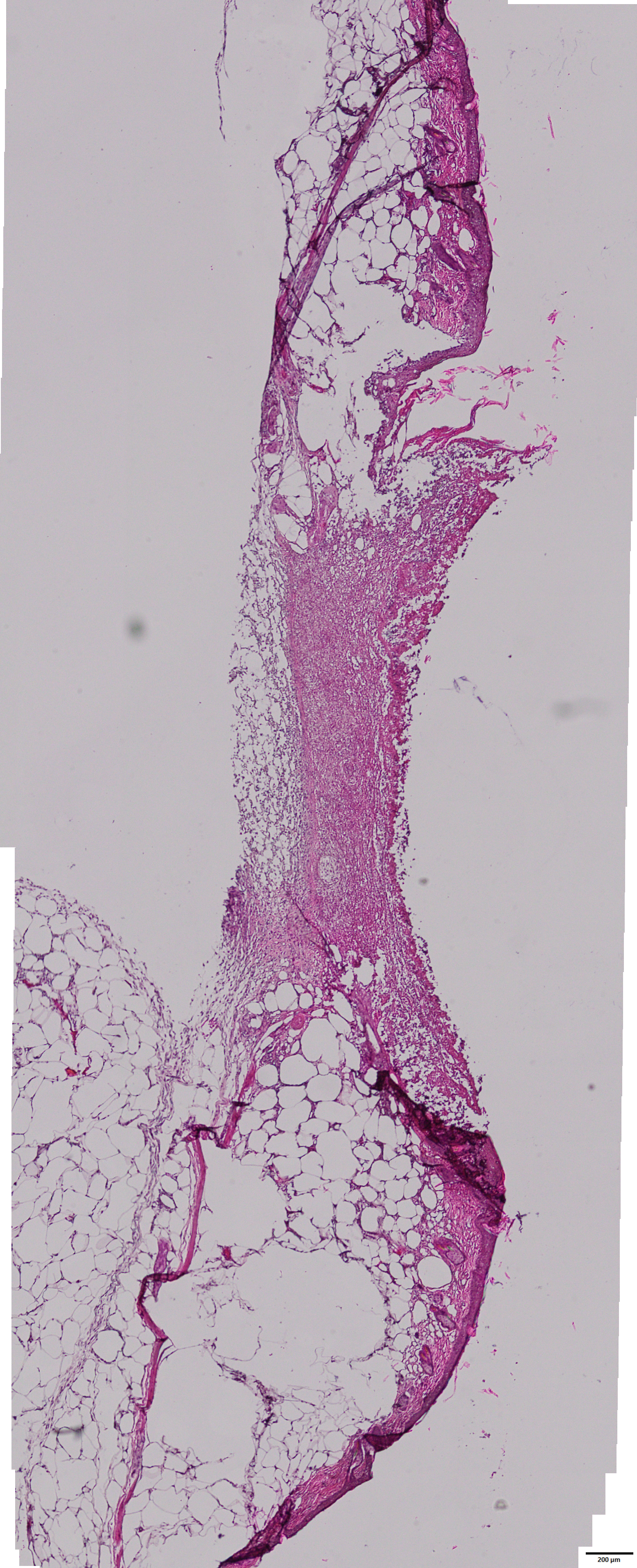

Supplement: S5 File — Fig 3B_wound images-2. (ZIP) [file pone.0339341.s010.zip › fig 3B_wound images-2/dbdb_day 7.tif]

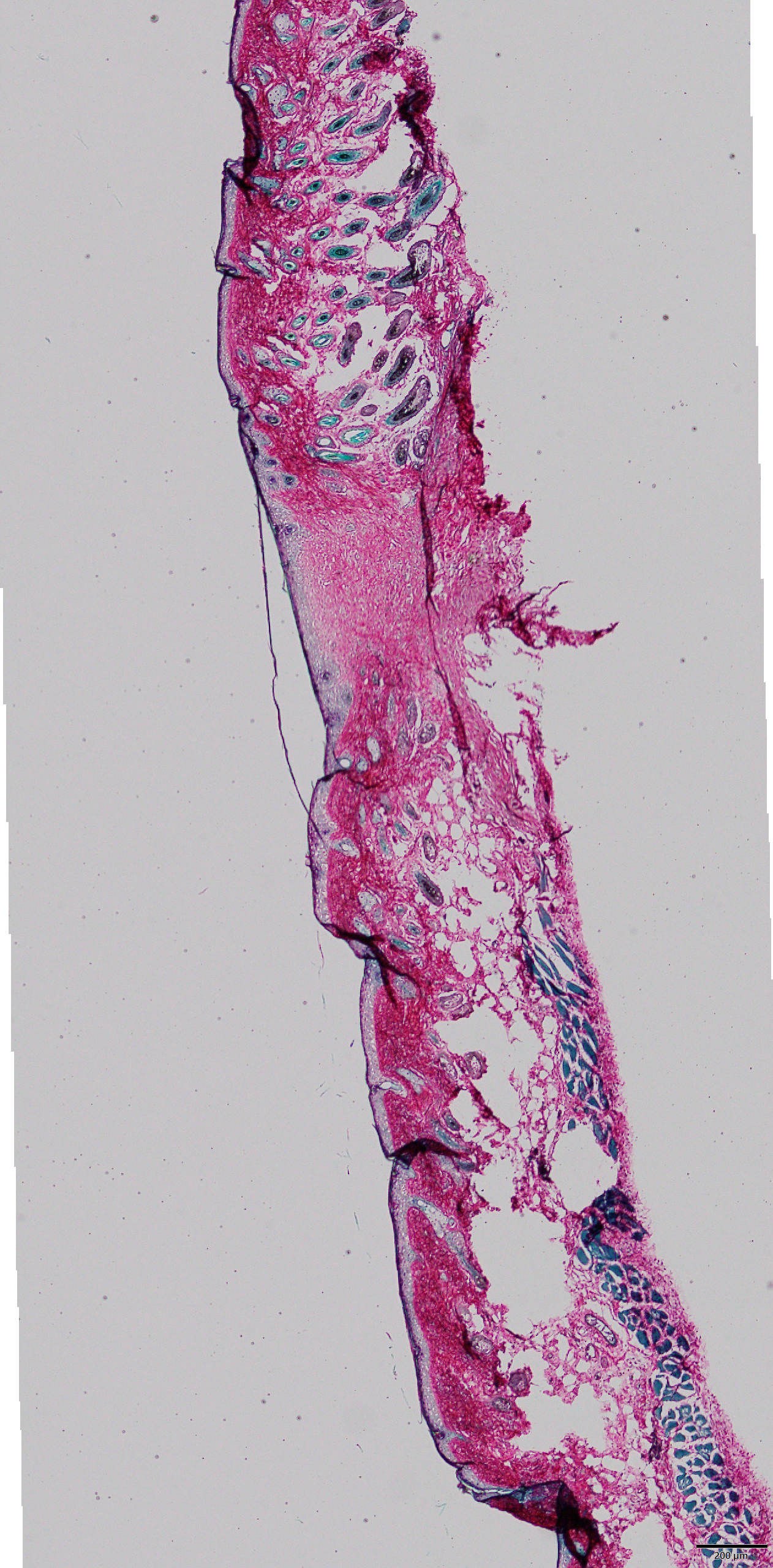

Supplement: S6 File — Fig 3D_wound images-1. (ZIP) [file pone.0339341.s011.zip › fig 3D_wound images-1/db+ estrogen_day 14.tif]

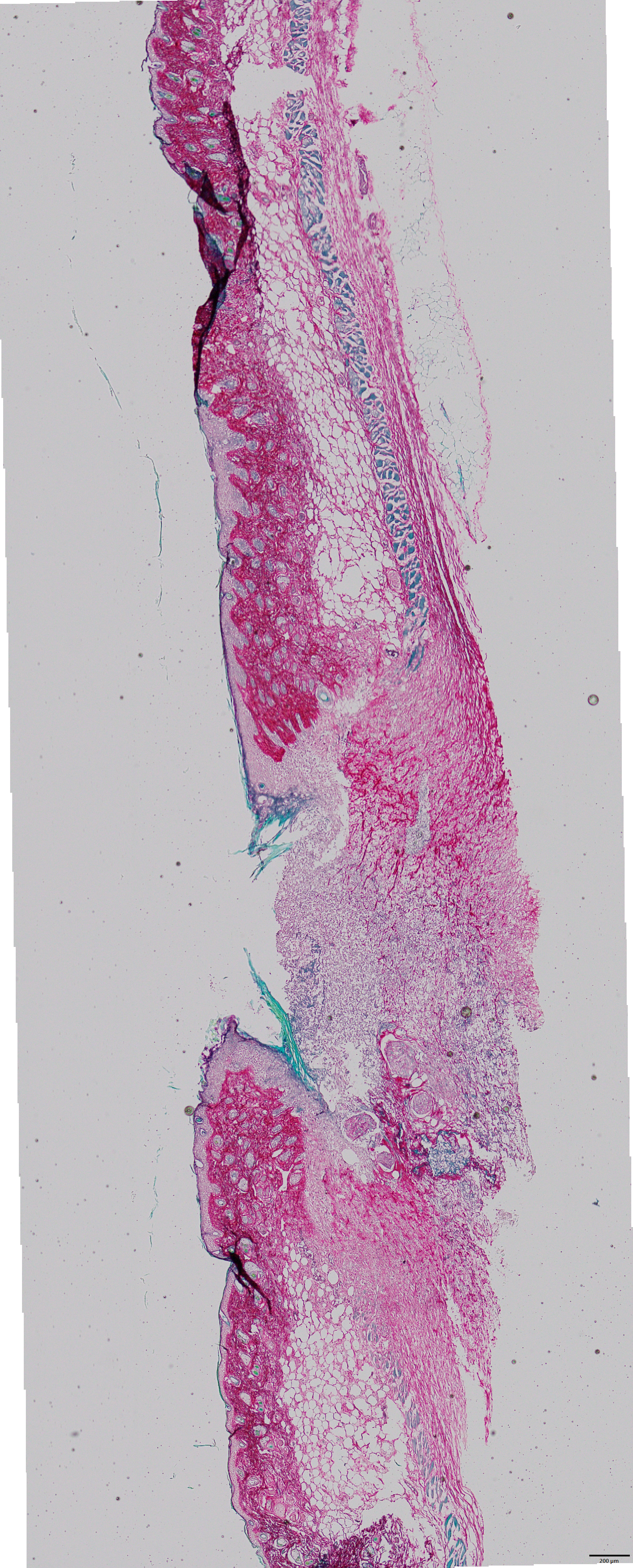

Supplement: S6 File — Fig 3D_wound images-1. (ZIP) [file pone.0339341.s011.zip › fig 3D_wound images-1/db+ estrogen_day 7.tif]

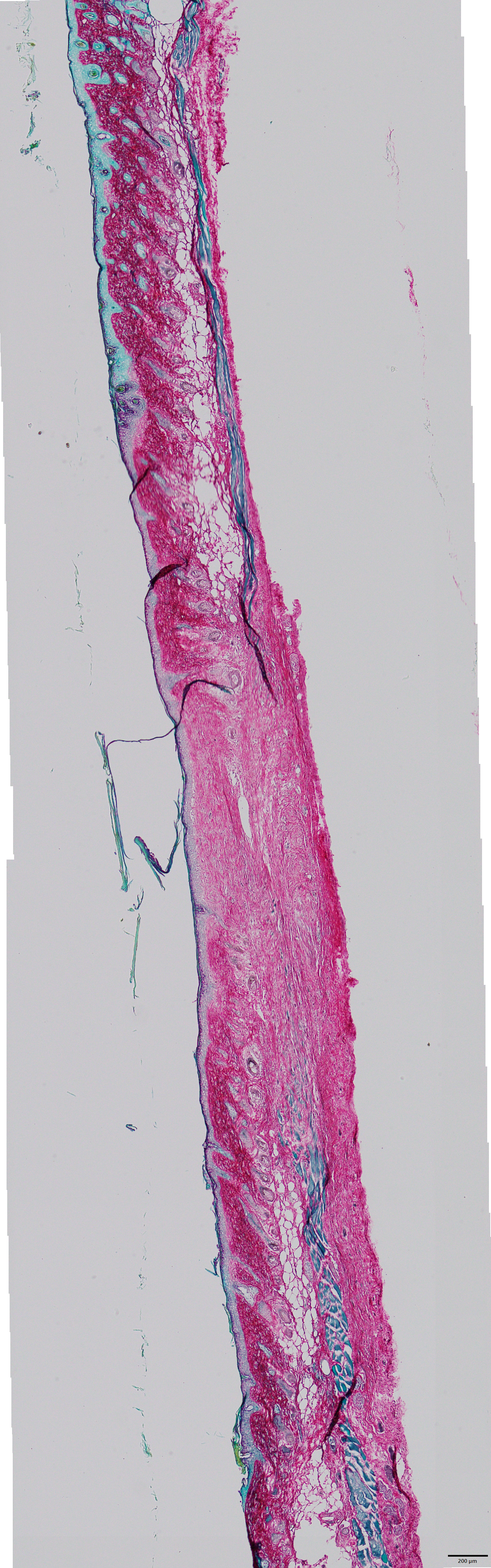

Supplement: S6 File — Fig 3D_wound images-1. (ZIP) [file pone.0339341.s011.zip › fig 3D_wound images-1/db+_day 14.tif]

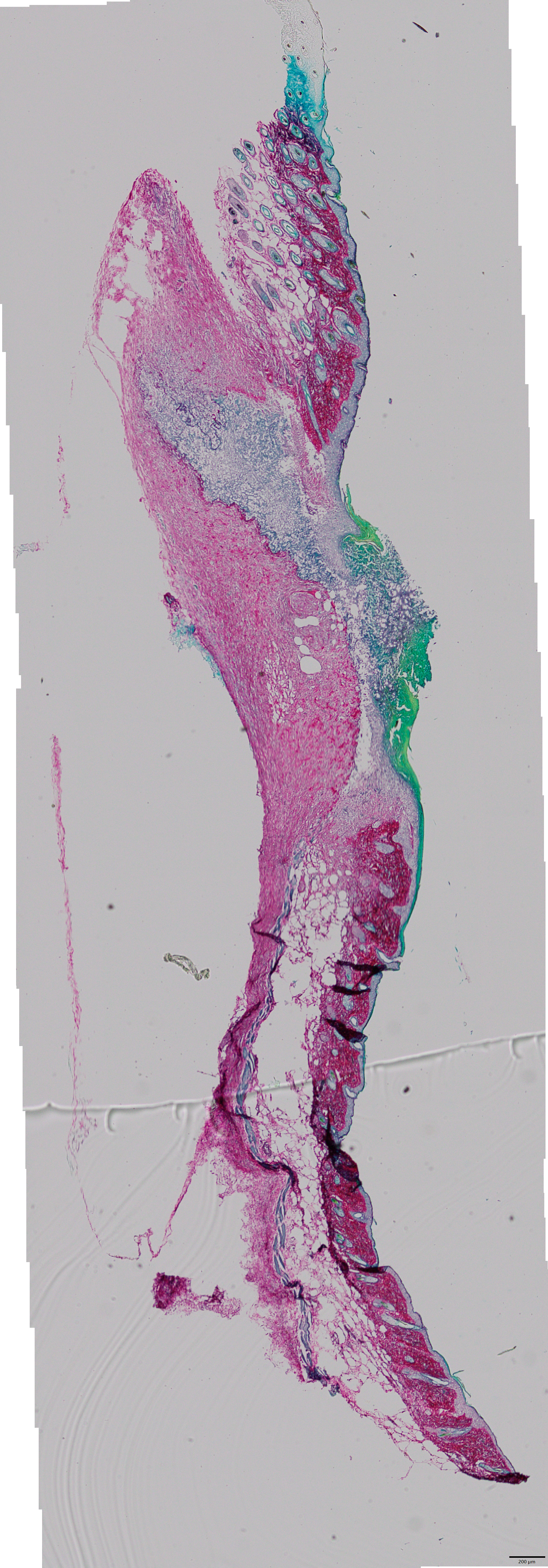

Supplement: S6 File — Fig 3D_wound images-1. (ZIP) [file pone.0339341.s011.zip › fig 3D_wound images-1/db+_day 7.tif]

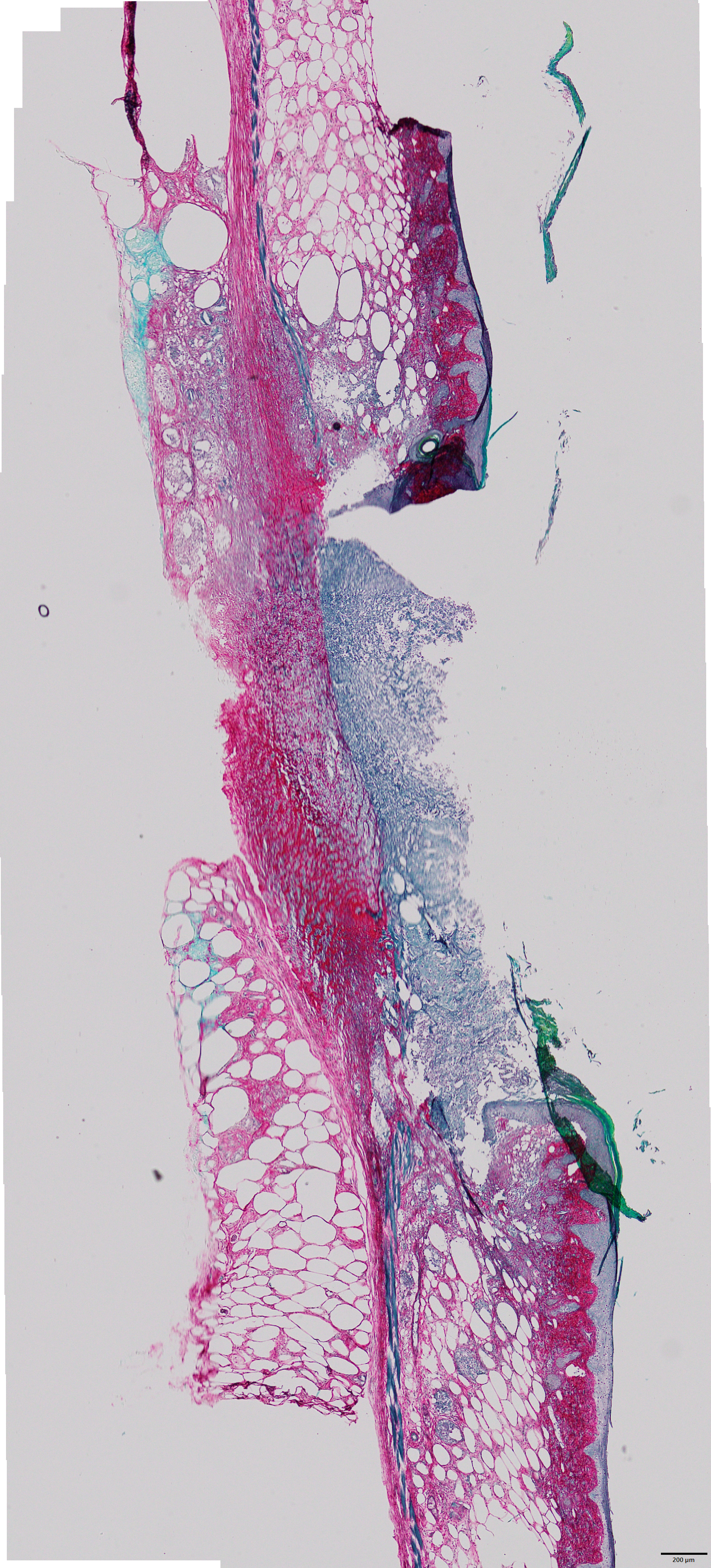

Supplement: S7 File — Fig 3D_wound images-2. (ZIP) [file pone.0339341.s012.zip › fig 3D_wound images-2/dbdb estrogen_day 14.tif]

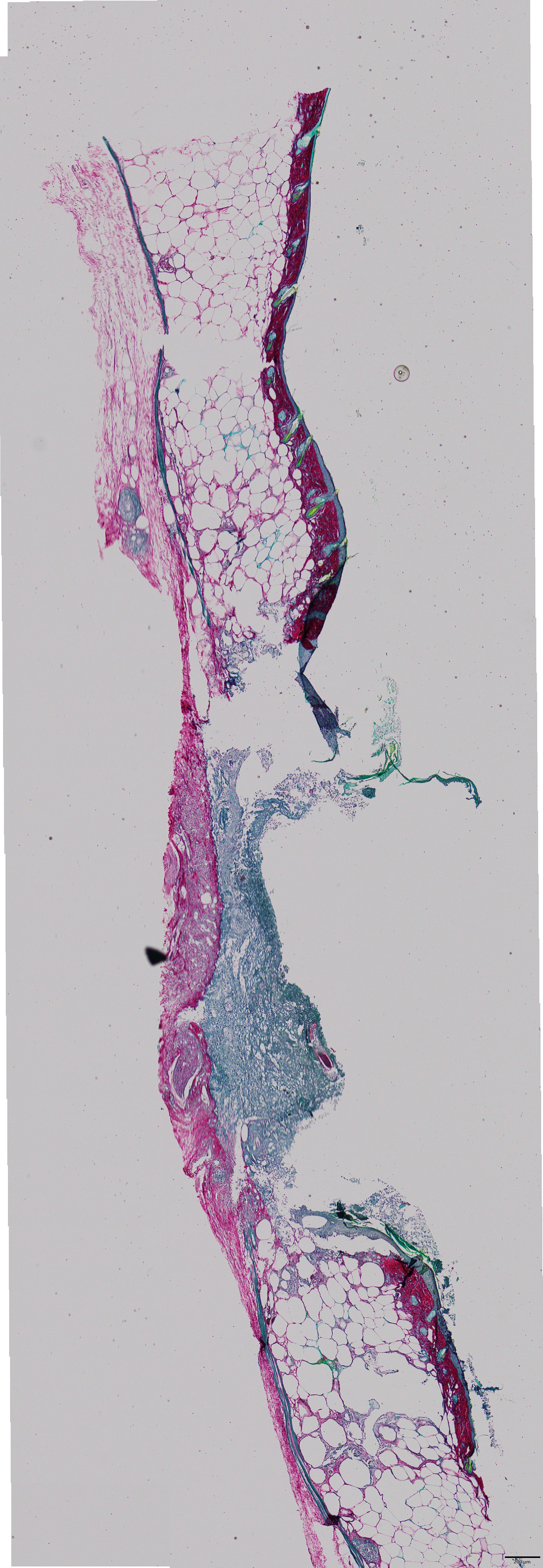

Supplement: S7 File — Fig 3D_wound images-2. (ZIP) [file pone.0339341.s012.zip › fig 3D_wound images-2/dbdb estrogen_day 7.tif]

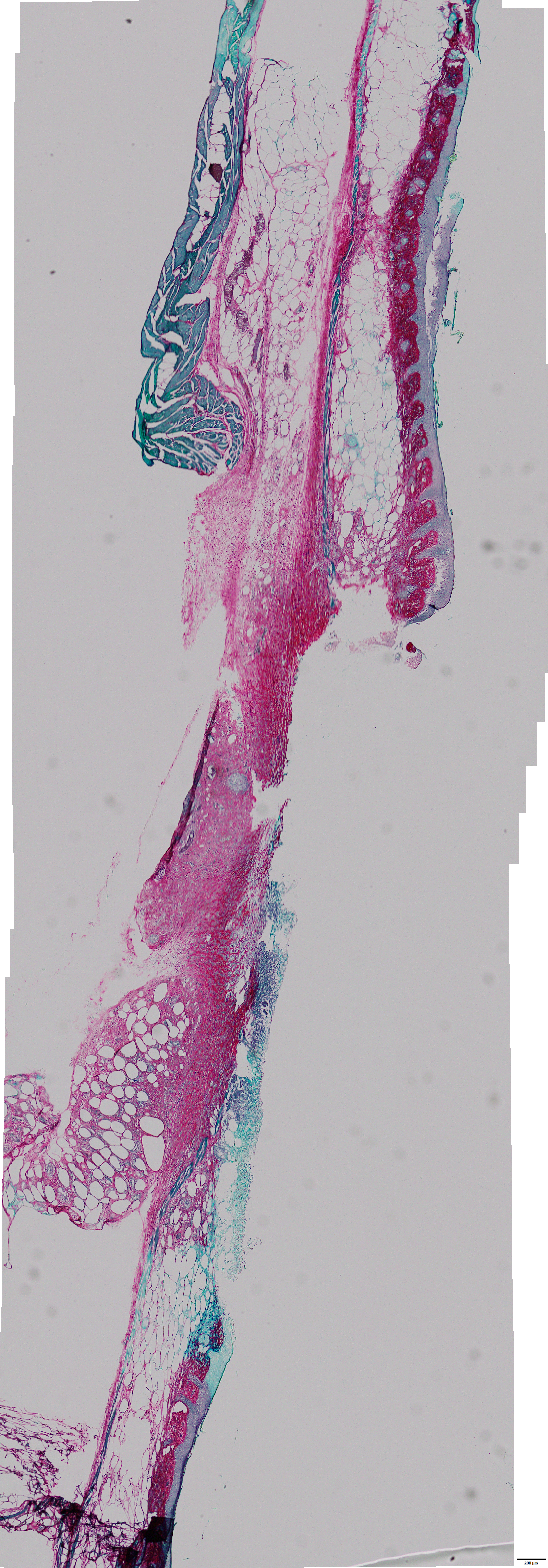

Supplement: S7 File — Fig 3D_wound images-2. (ZIP) [file pone.0339341.s012.zip › fig 3D_wound images-2/dbdb_day 14.tif]

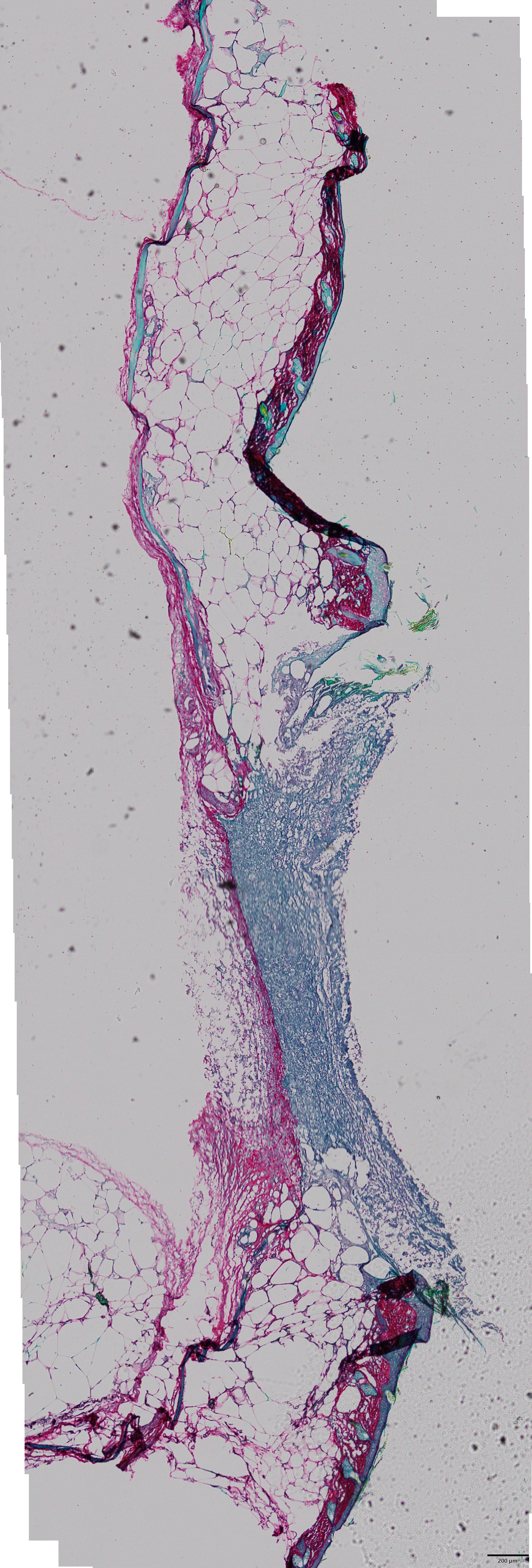

Supplement: S7 File — Fig 3D_wound images-2. (ZIP) [file pone.0339341.s012.zip › fig 3D_wound images-2/dbdb_day 7.tif]

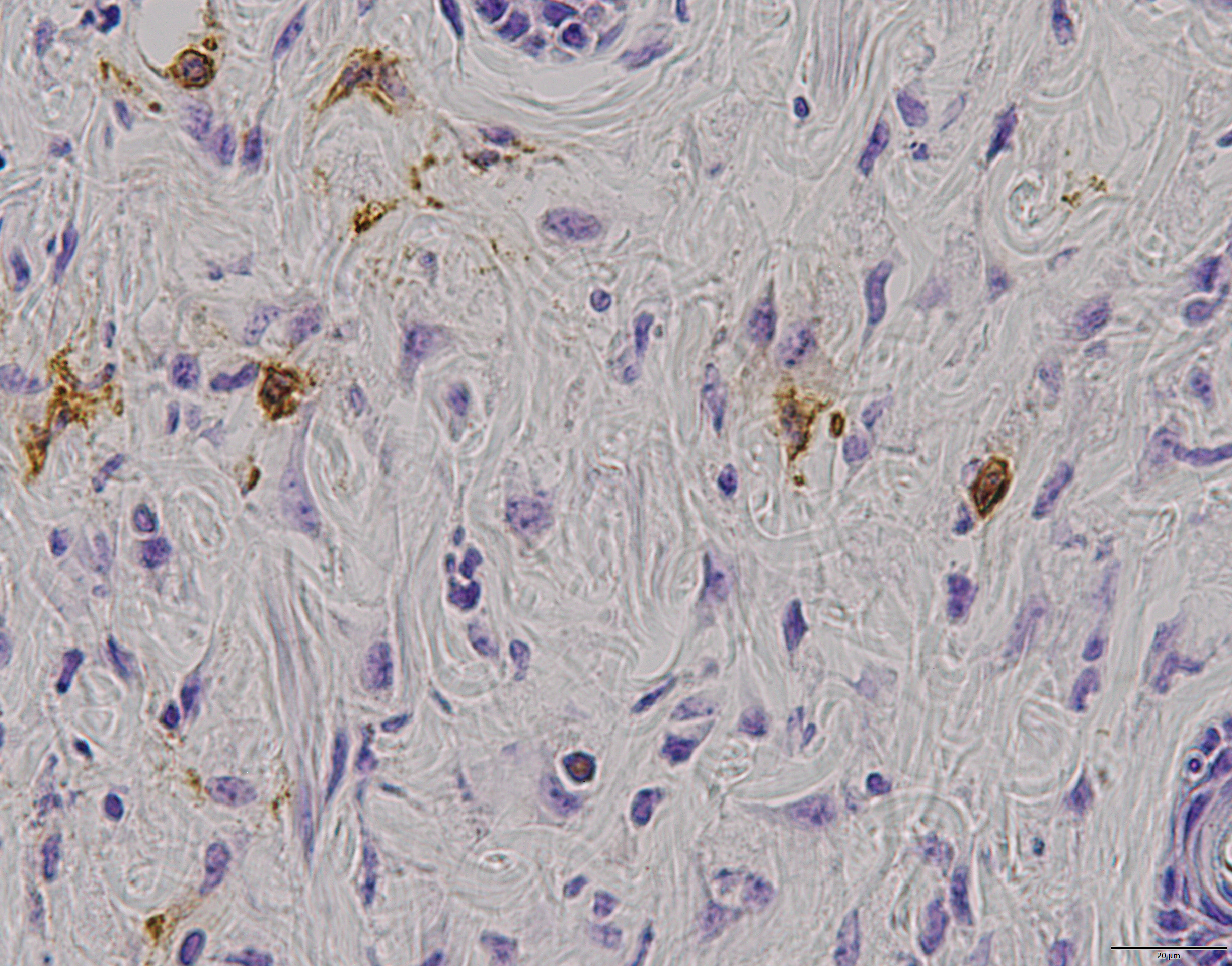

Supplement: S8 File — Fig 4B_wound images. (ZIP) [file pone.0339341.s013.zip › fig 4B_wound images/db+ estrogen_day 14.tif]

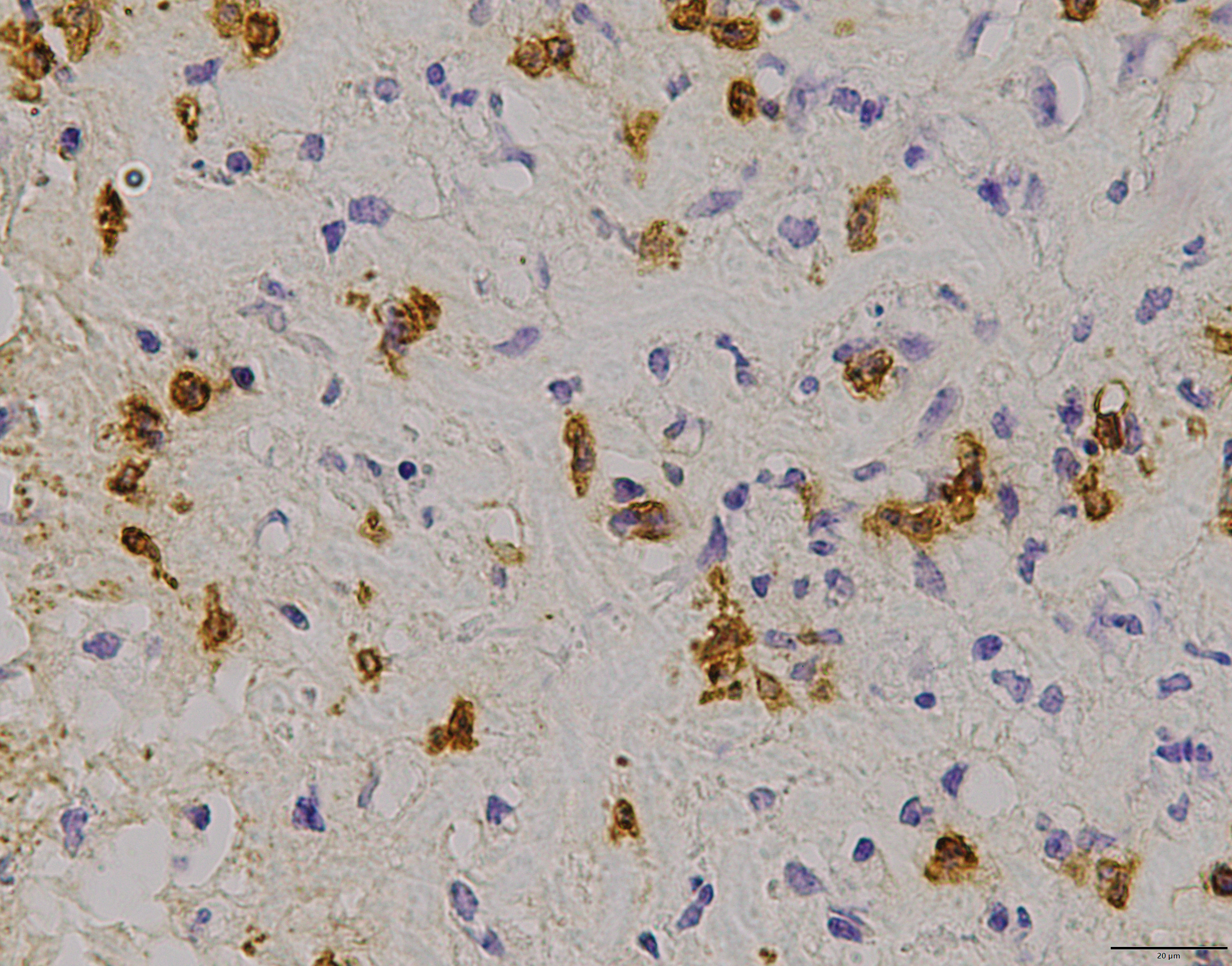

Supplement: S8 File — Fig 4B_wound images. (ZIP) [file pone.0339341.s013.zip › fig 4B_wound images/db+ estrogen_day 7.tif]

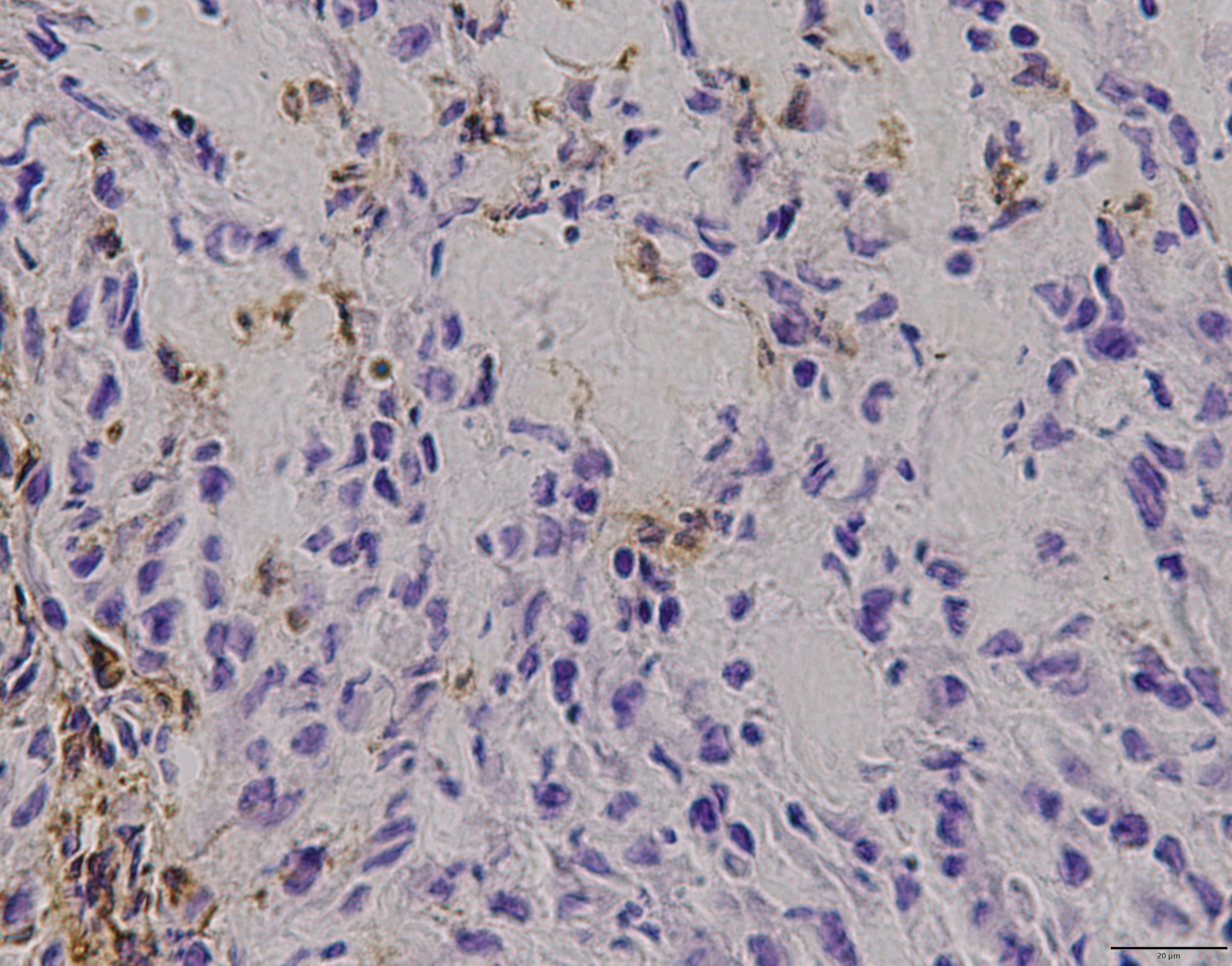

Supplement: S8 File — Fig 4B_wound images. (ZIP) [file pone.0339341.s013.zip › fig 4B_wound images/db+_day 14.tif]

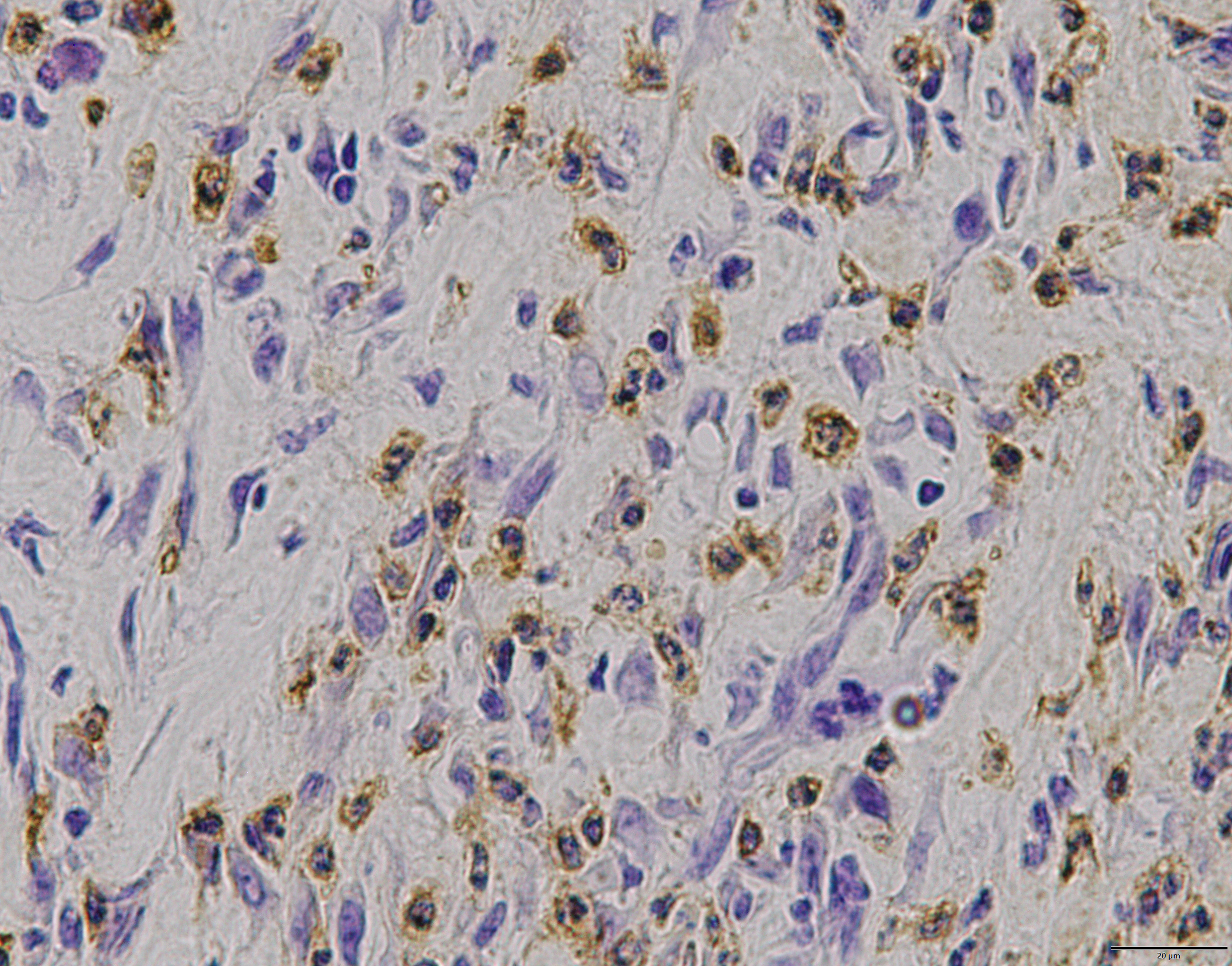

Supplement: S8 File — Fig 4B_wound images. (ZIP) [file pone.0339341.s013.zip › fig 4B_wound images/db+_day 7.tif]

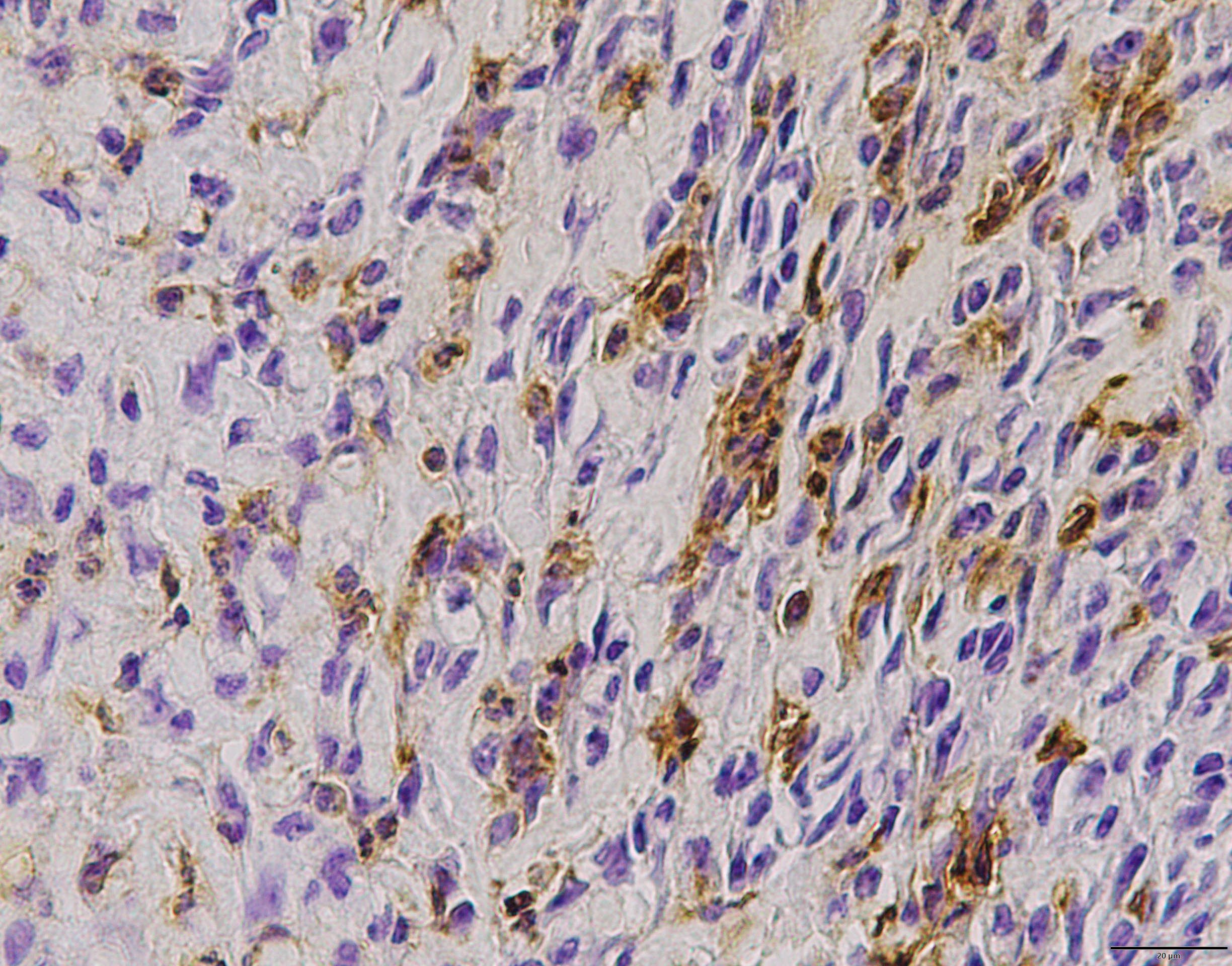

Supplement: S8 File — Fig 4B_wound images. (ZIP) [file pone.0339341.s013.zip › fig 4B_wound images/dbdb estrogen_day 14.tif]

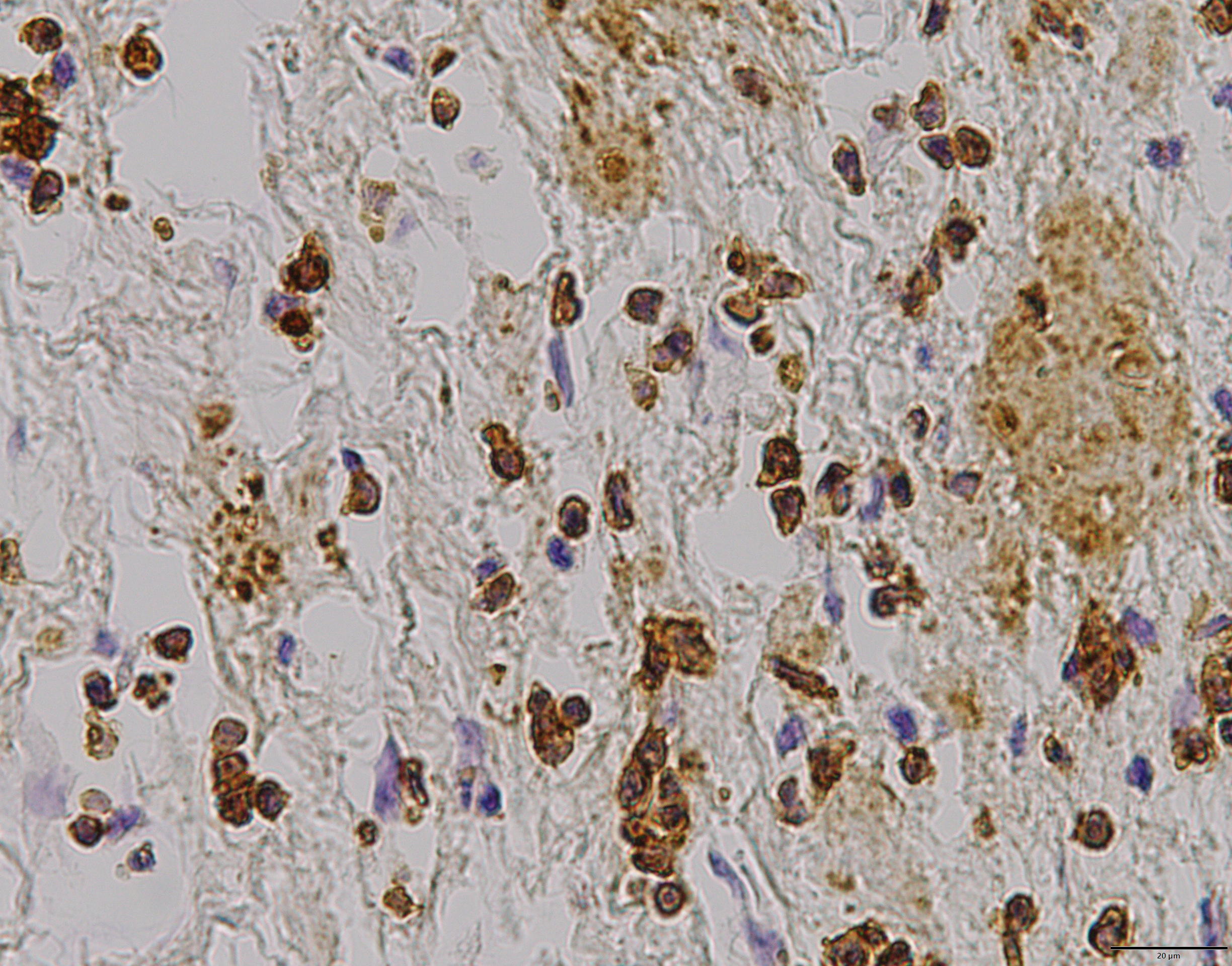

Supplement: S8 File — Fig 4B_wound images. (ZIP) [file pone.0339341.s013.zip › fig 4B_wound images/dbdb estrogen_day 7.tif]

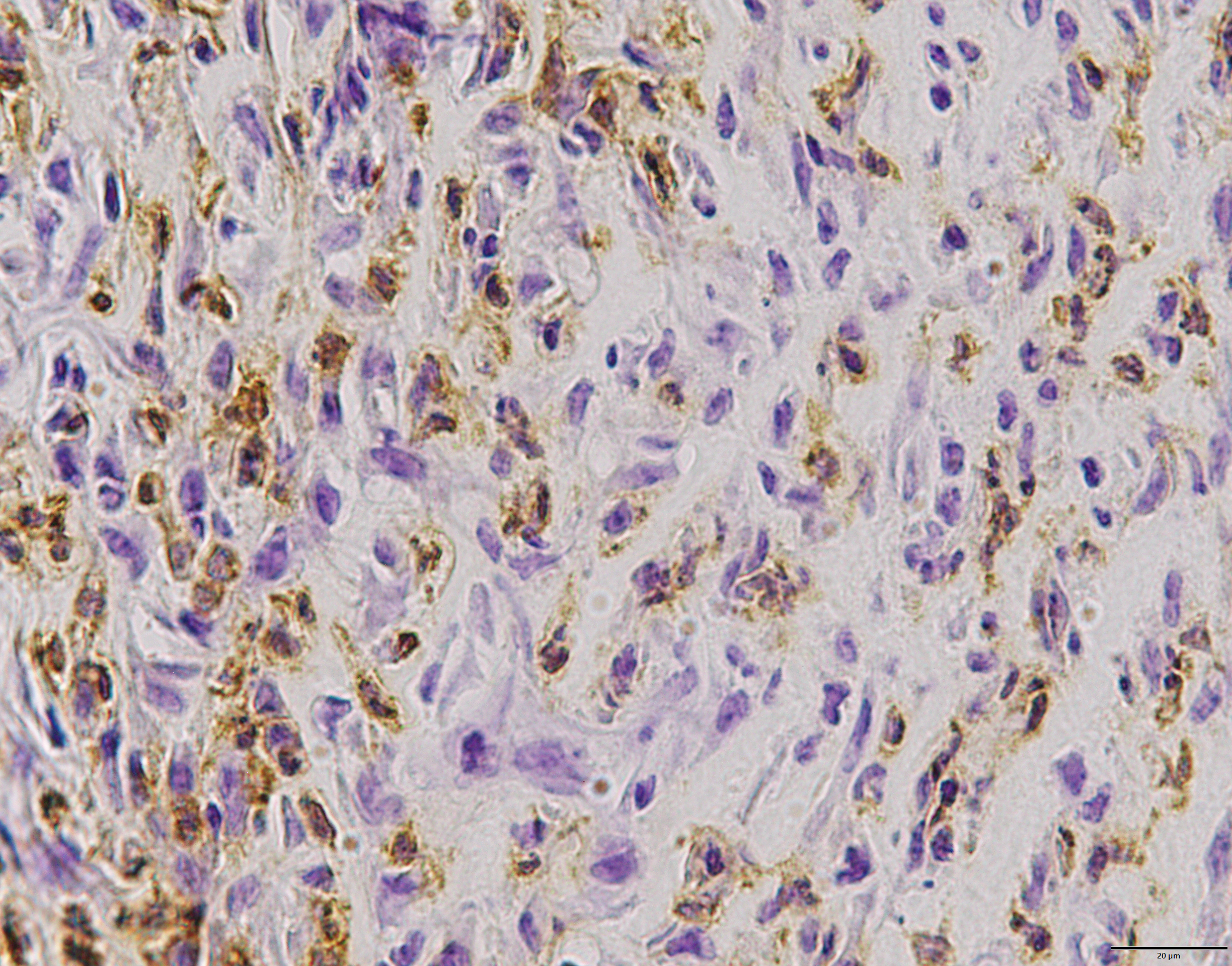

Supplement: S8 File — Fig 4B_wound images. (ZIP) [file pone.0339341.s013.zip › fig 4B_wound images/dbdb_day 14.tif]

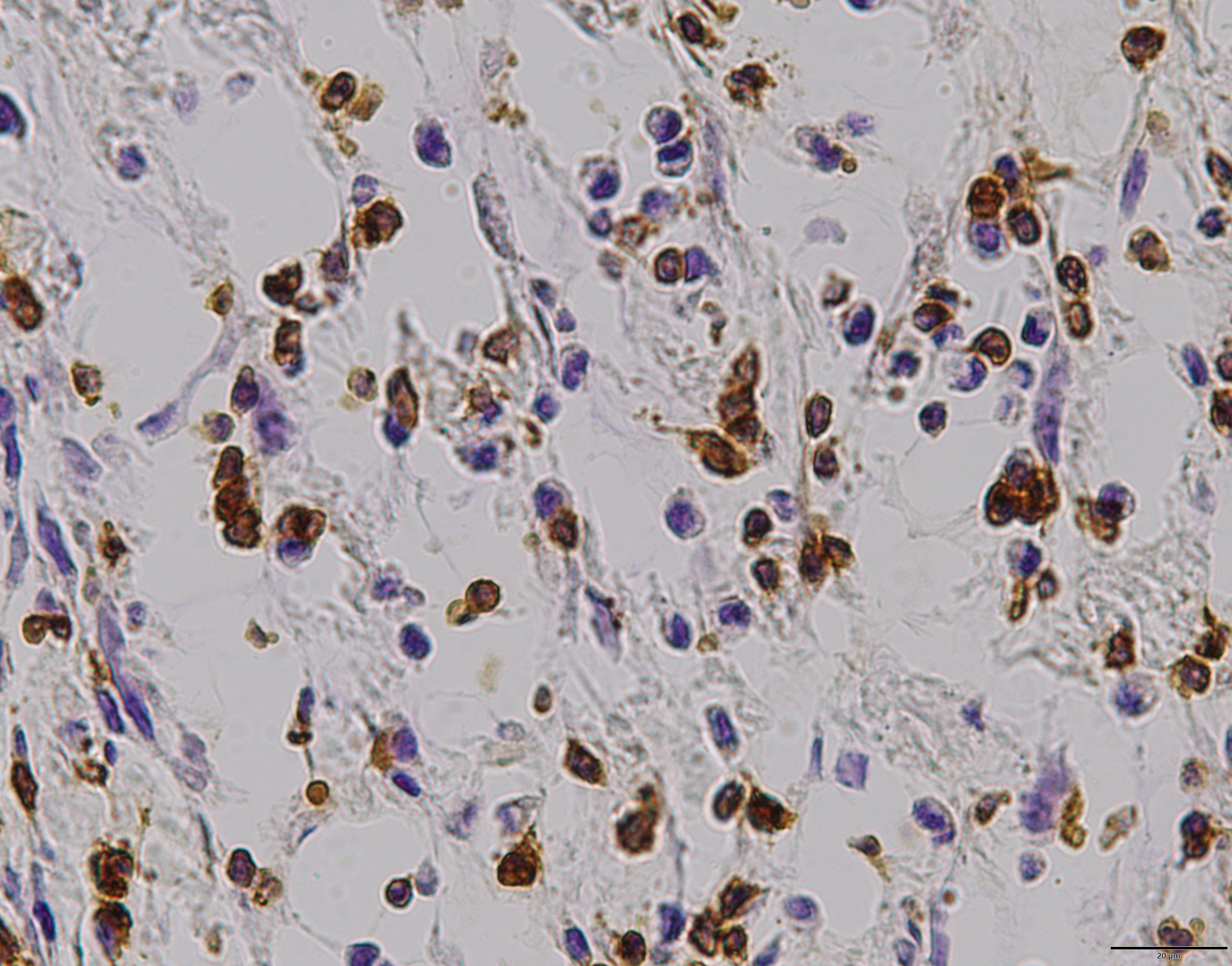

Supplement: S8 File — Fig 4B_wound images. (ZIP) [file pone.0339341.s013.zip › fig 4B_wound images/dbdb_day 7.tif]

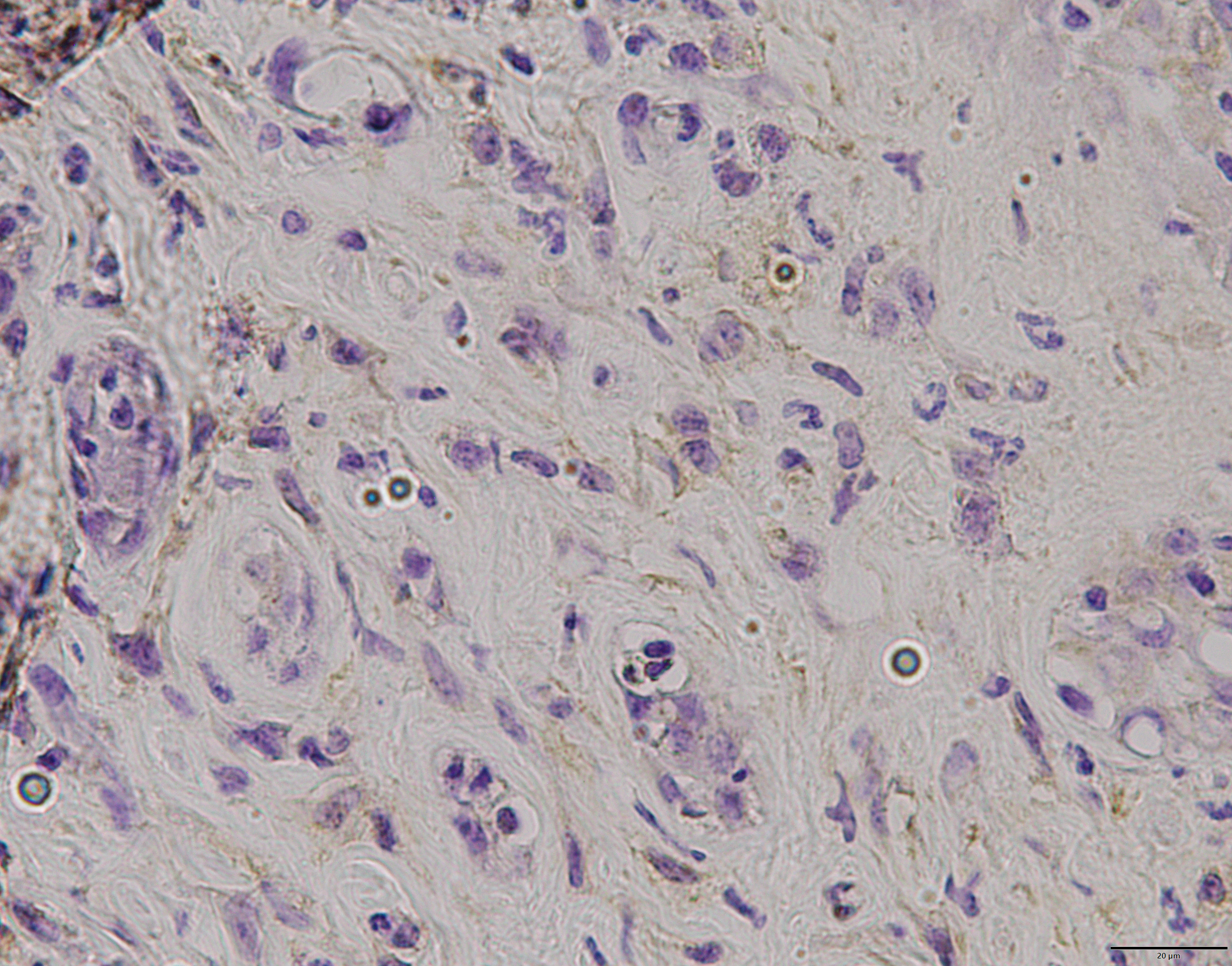

Supplement: S9 File — Fig 4D_wound images. (ZIP) [file pone.0339341.s014.zip › fig 4D_wound images/db+ estrogen_day 14.tif]

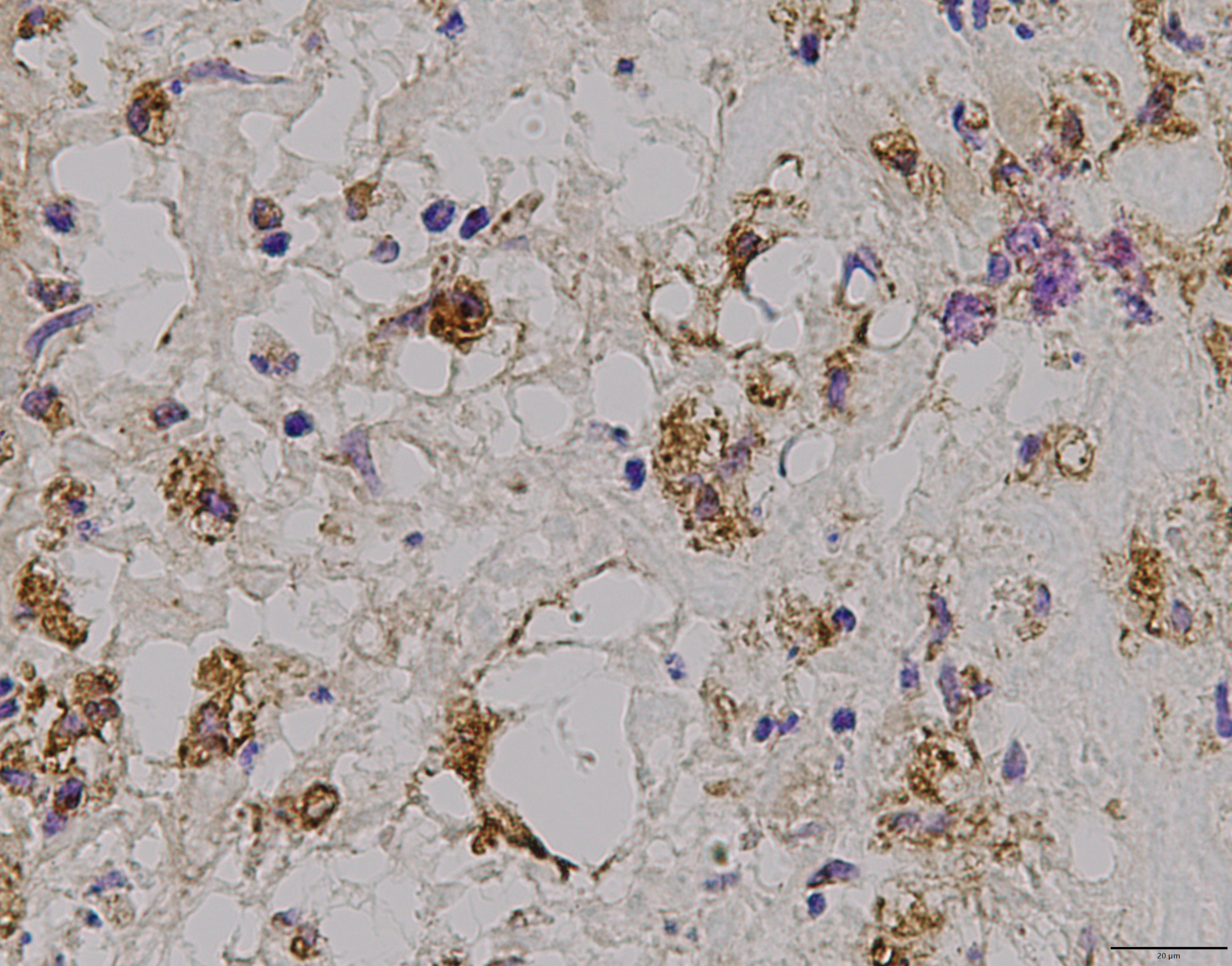

Supplement: S9 File — Fig 4D_wound images. (ZIP) [file pone.0339341.s014.zip › fig 4D_wound images/db+ estrogen_day 7.tif]

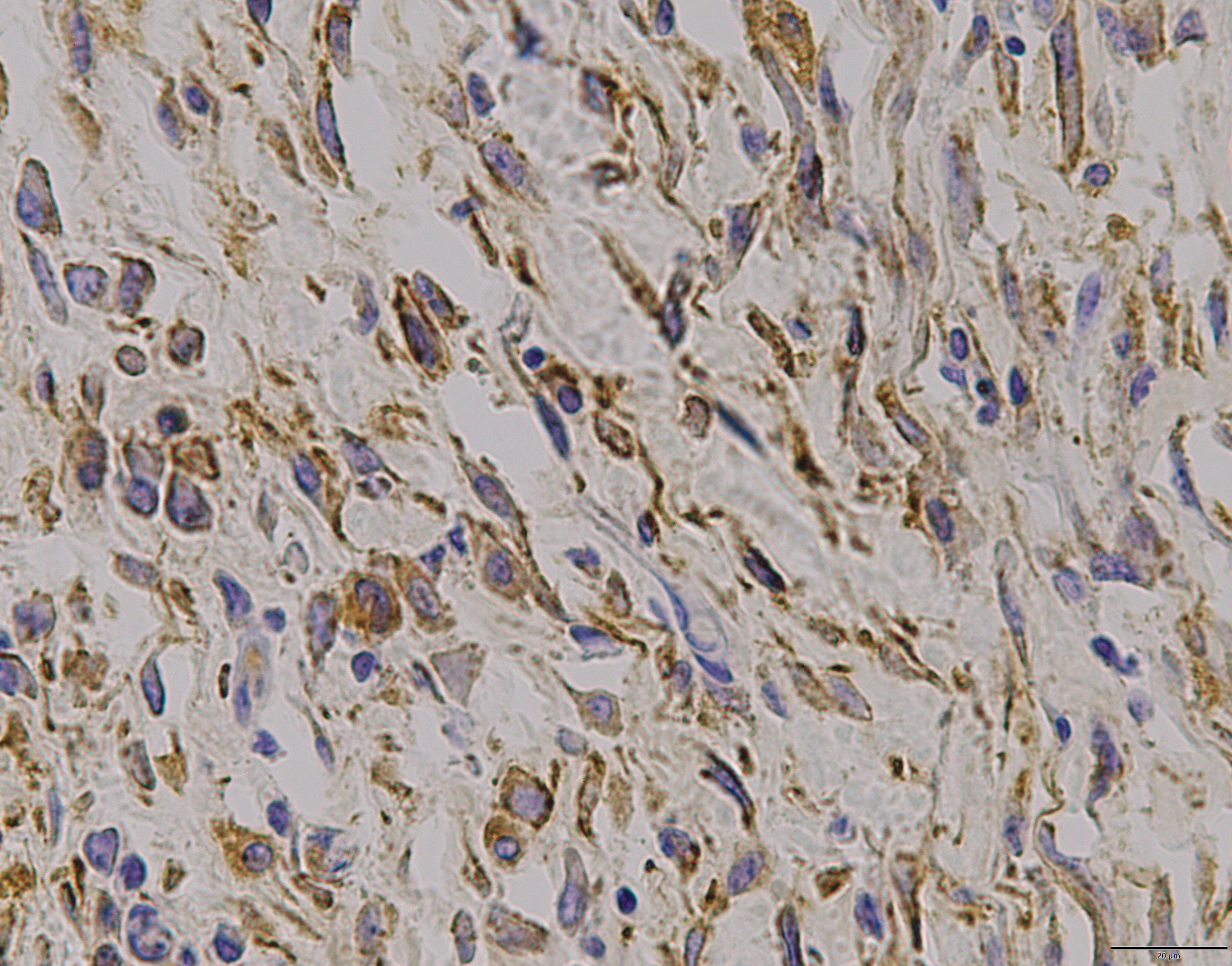

Supplement: S9 File — Fig 4D_wound images. (ZIP) [file pone.0339341.s014.zip › fig 4D_wound images/db+_day 14.tif]

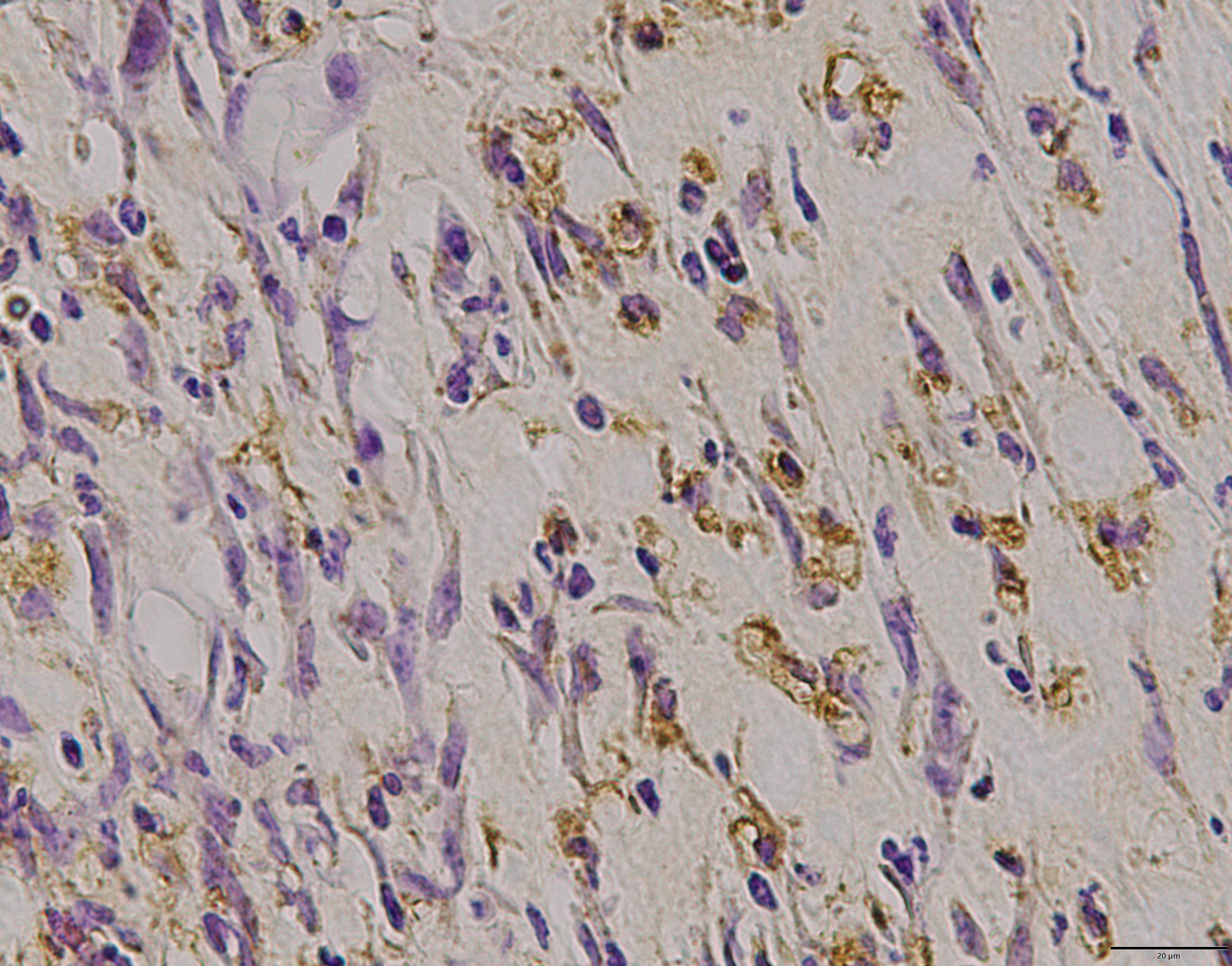

Supplement: S9 File — Fig 4D_wound images. (ZIP) [file pone.0339341.s014.zip › fig 4D_wound images/db+_day 7.tif]

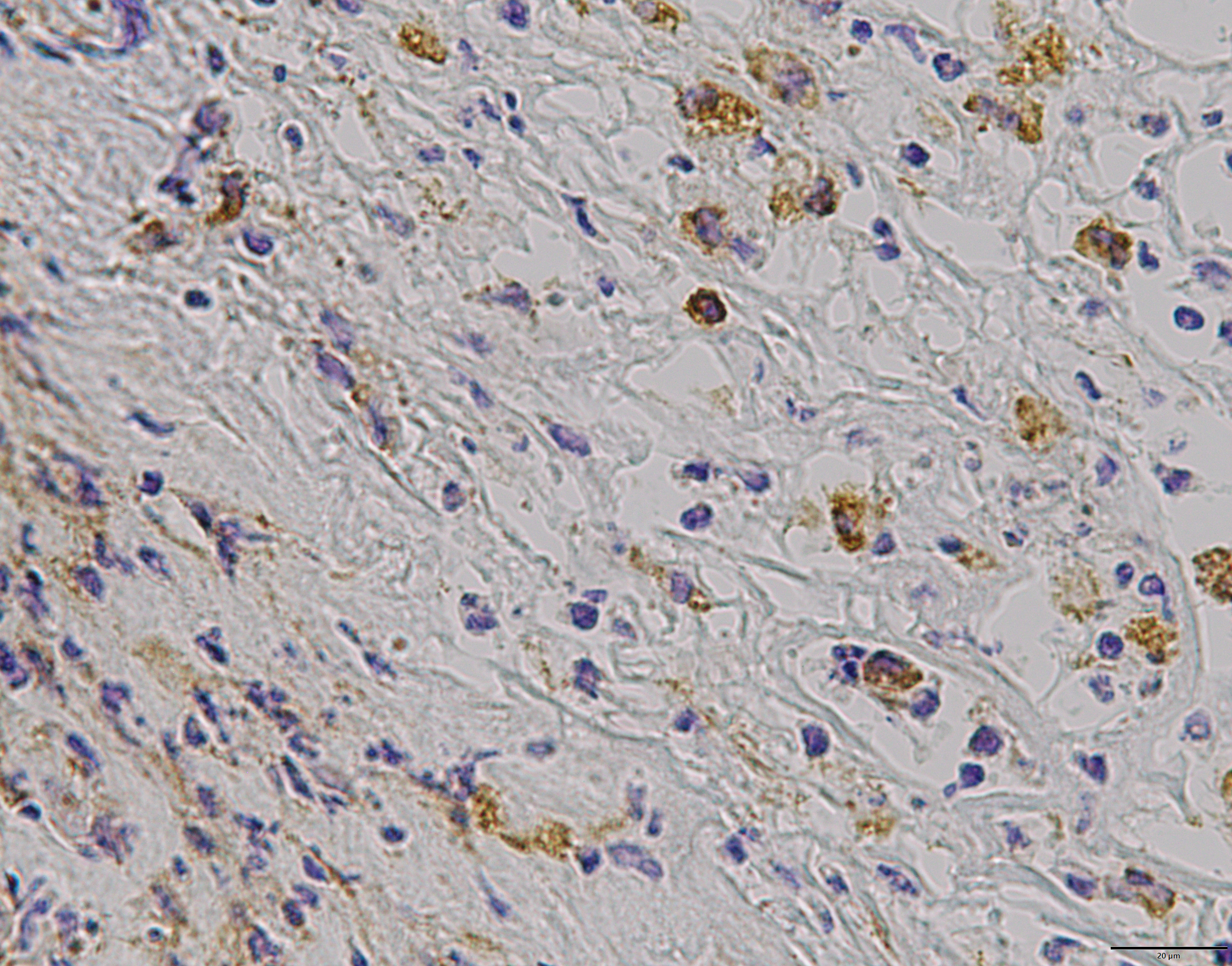

Supplement: S9 File — Fig 4D_wound images. (ZIP) [file pone.0339341.s014.zip › fig 4D_wound images/dbdb estrogen_day 14.tif]

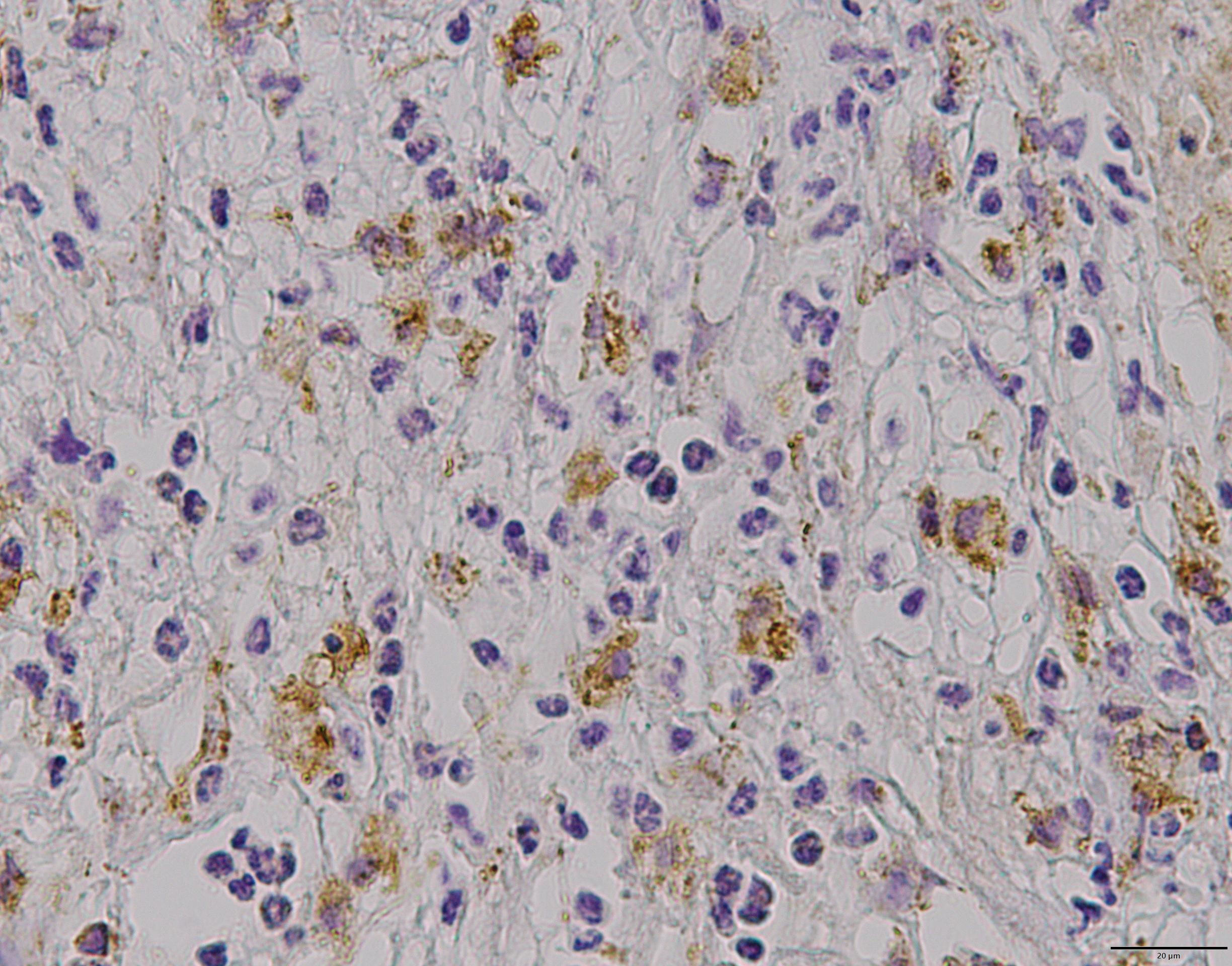

Supplement: S9 File — Fig 4D_wound images. (ZIP) [file pone.0339341.s014.zip › fig 4D_wound images/dbdb estrogen_day 7.tif]

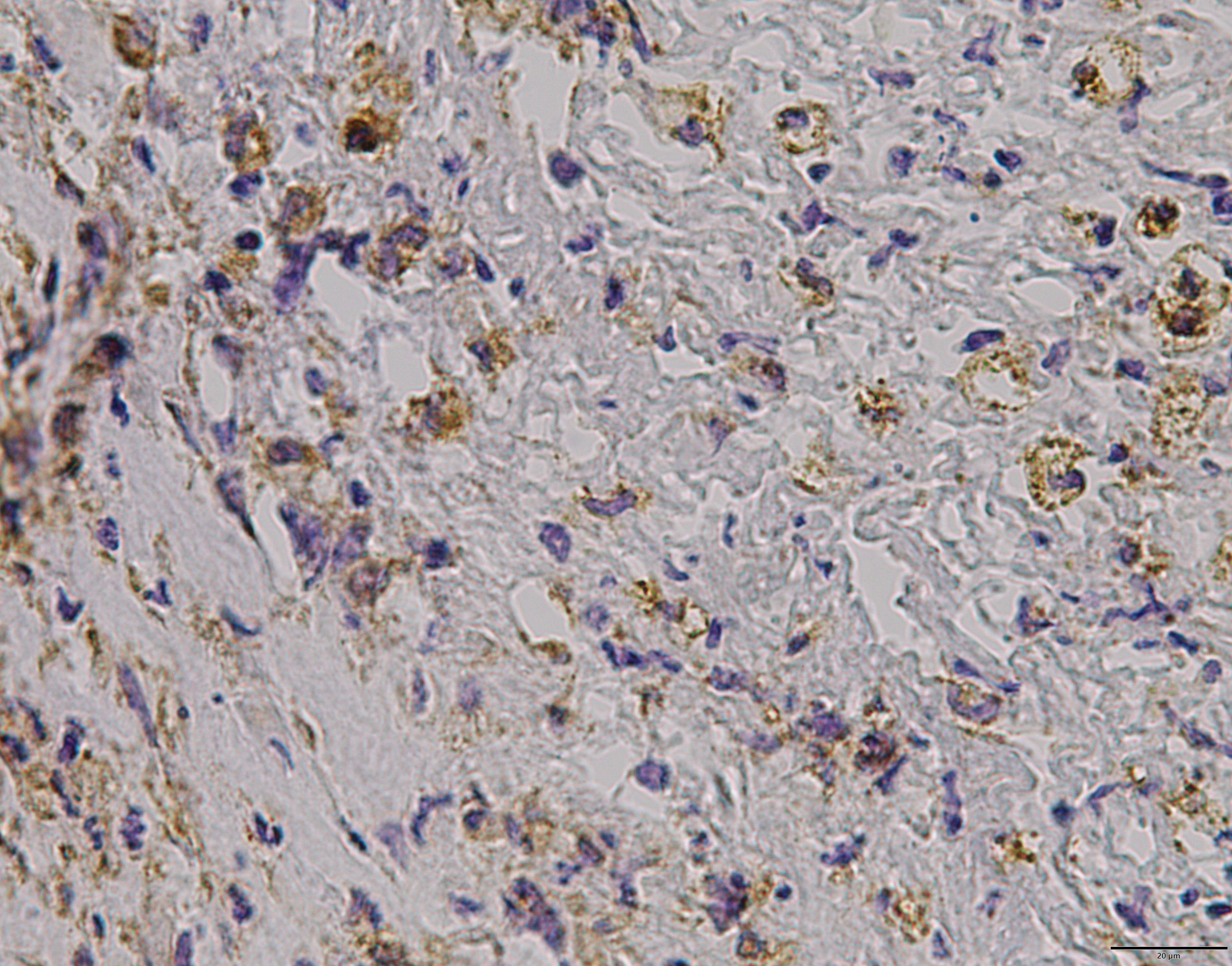

Supplement: S9 File — Fig 4D_wound images. (ZIP) [file pone.0339341.s014.zip › fig 4D_wound images/dbdb_day 14.tif]

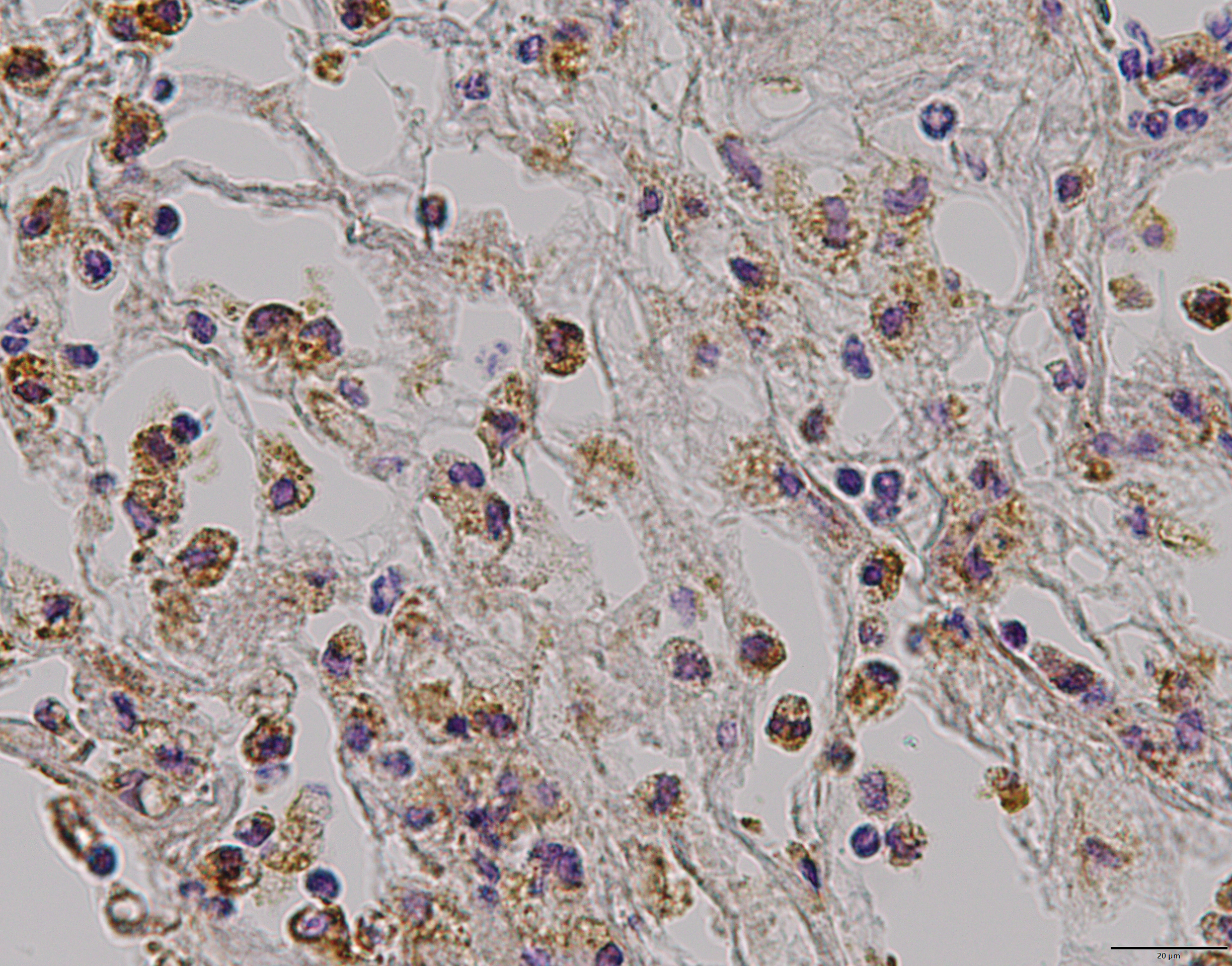

Supplement: S9 File — Fig 4D_wound images. (ZIP) [file pone.0339341.s014.zip › fig 4D_wound images/dbdb_day 7.tif]

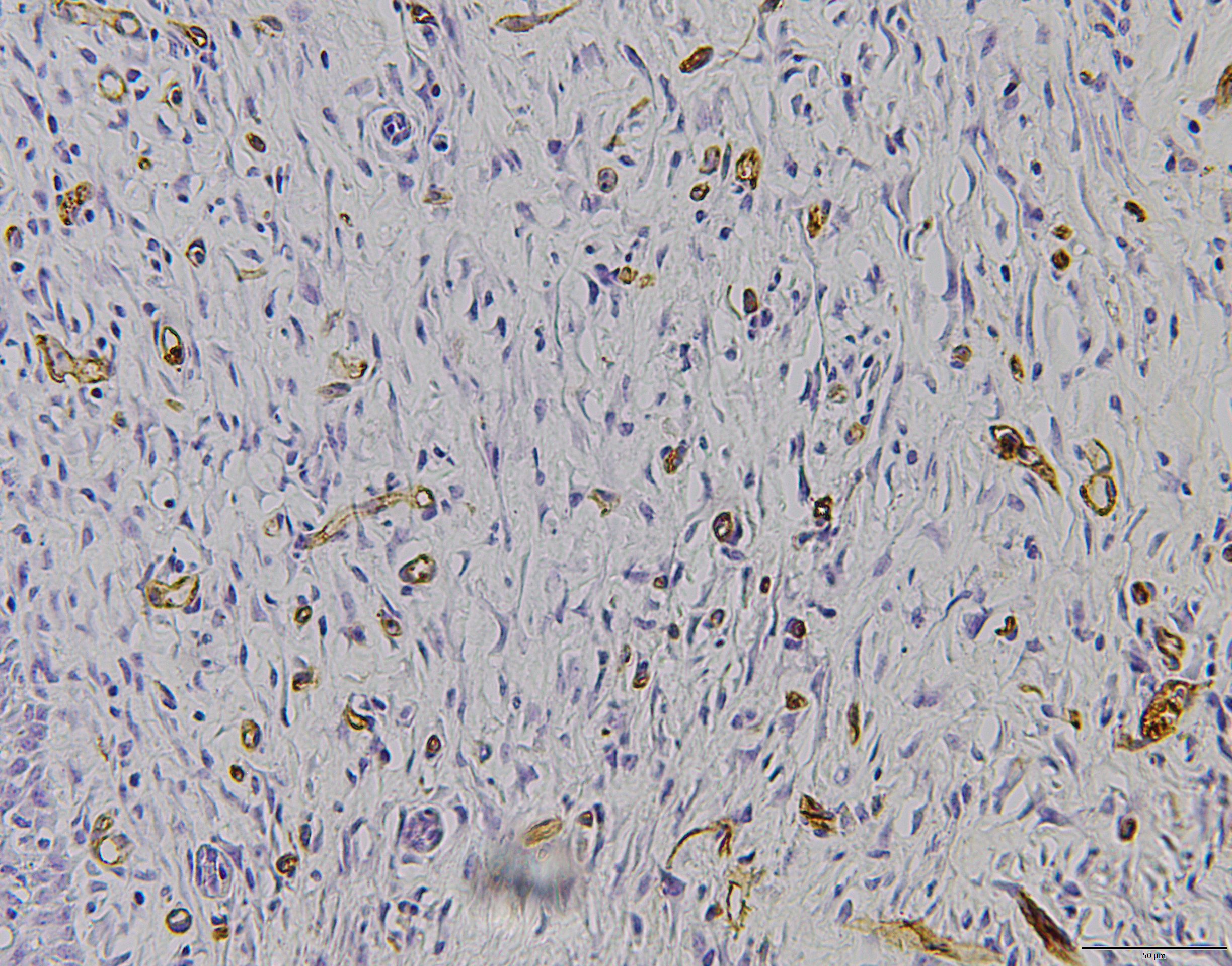

Supplement: S10 File — Fig 5B_wound images. (ZIP) [file pone.0339341.s015.zip › fig 5B_wound images/db+ estrogen_day 14.tif]

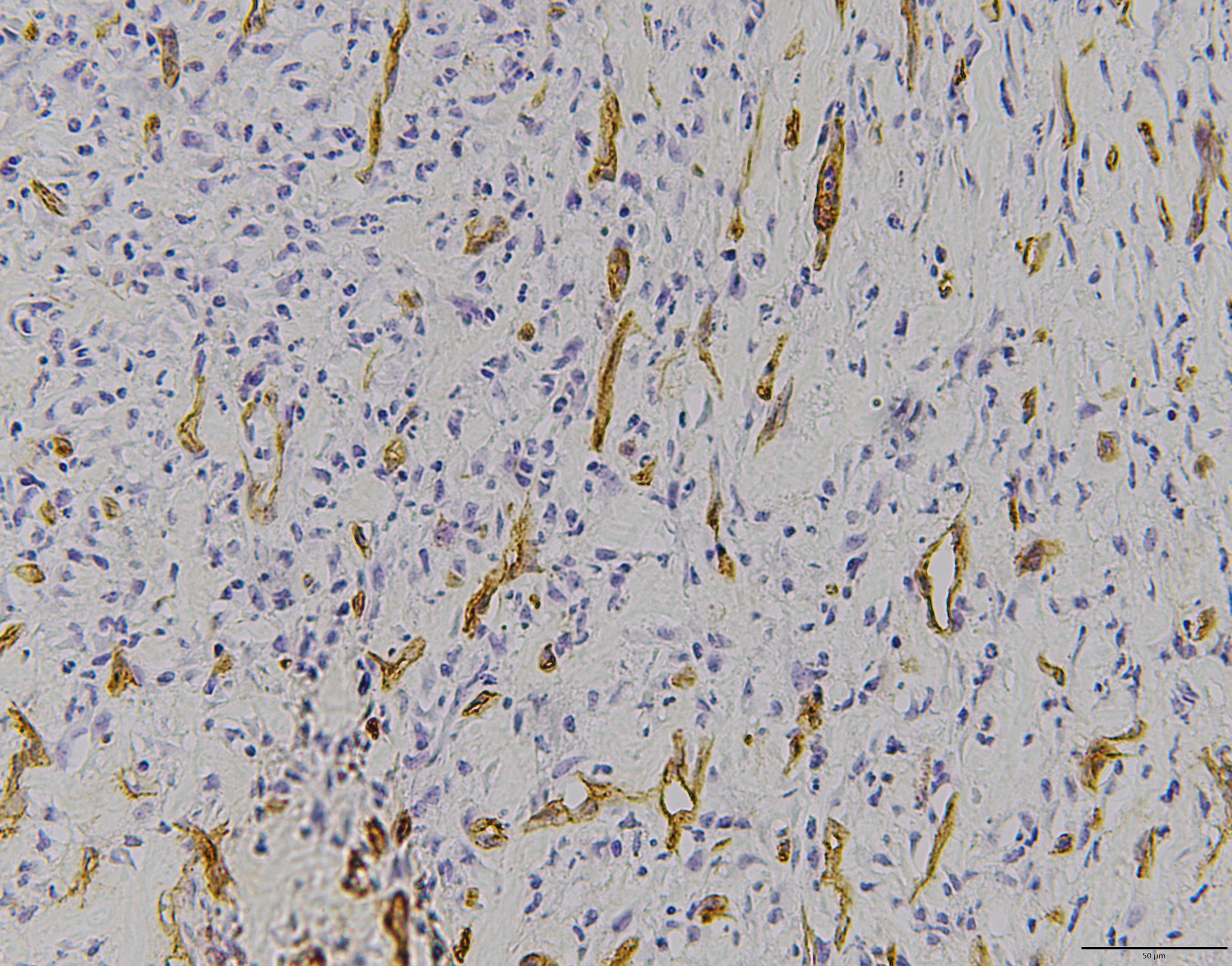

Supplement: S10 File — Fig 5B_wound images. (ZIP) [file pone.0339341.s015.zip › fig 5B_wound images/db+ estrogen_day 7.tif]

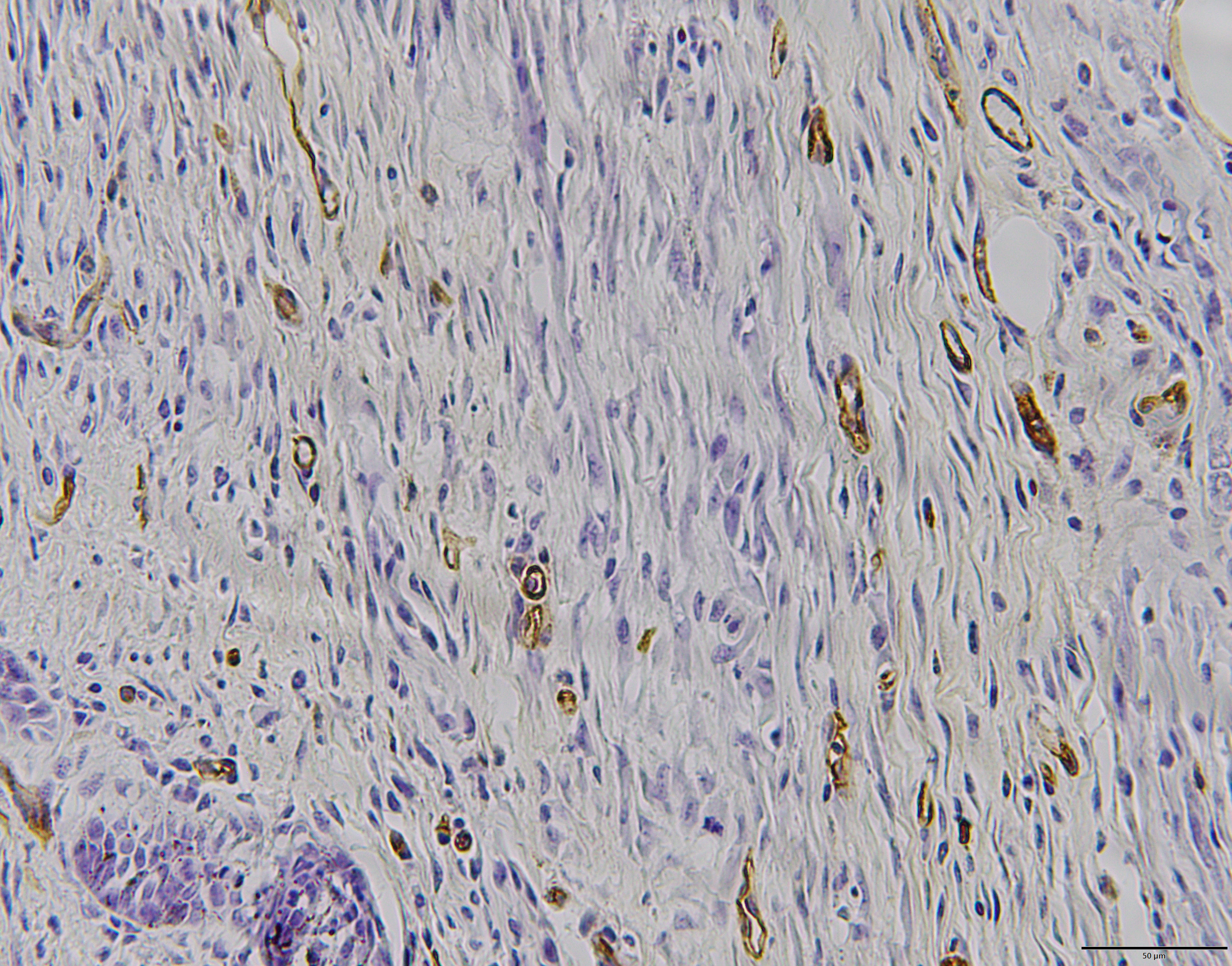

Supplement: S10 File — Fig 5B_wound images. (ZIP) [file pone.0339341.s015.zip › fig 5B_wound images/db+_day 14.tif]

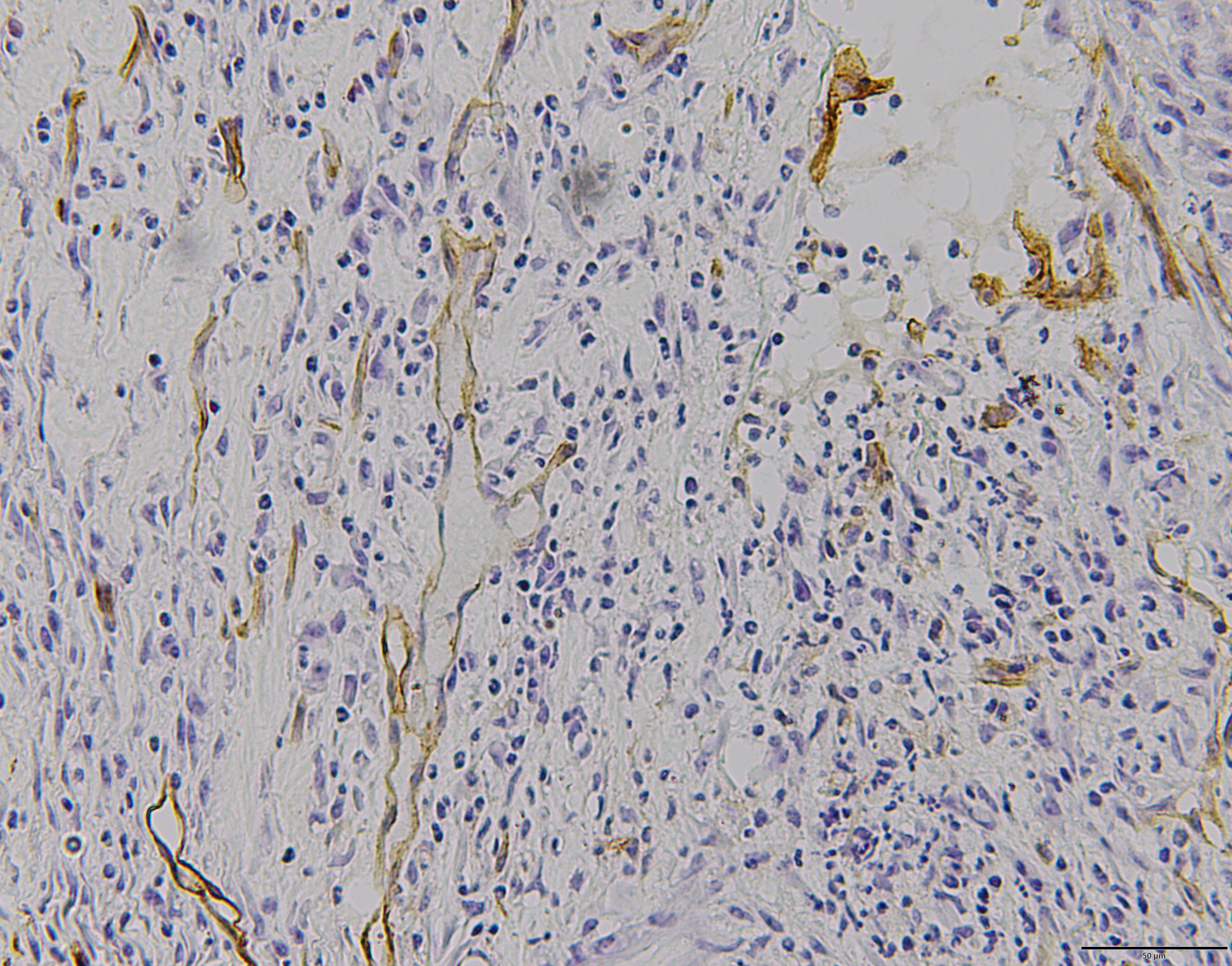

Supplement: S10 File — Fig 5B_wound images. (ZIP) [file pone.0339341.s015.zip › fig 5B_wound images/db+_day 7.tif]

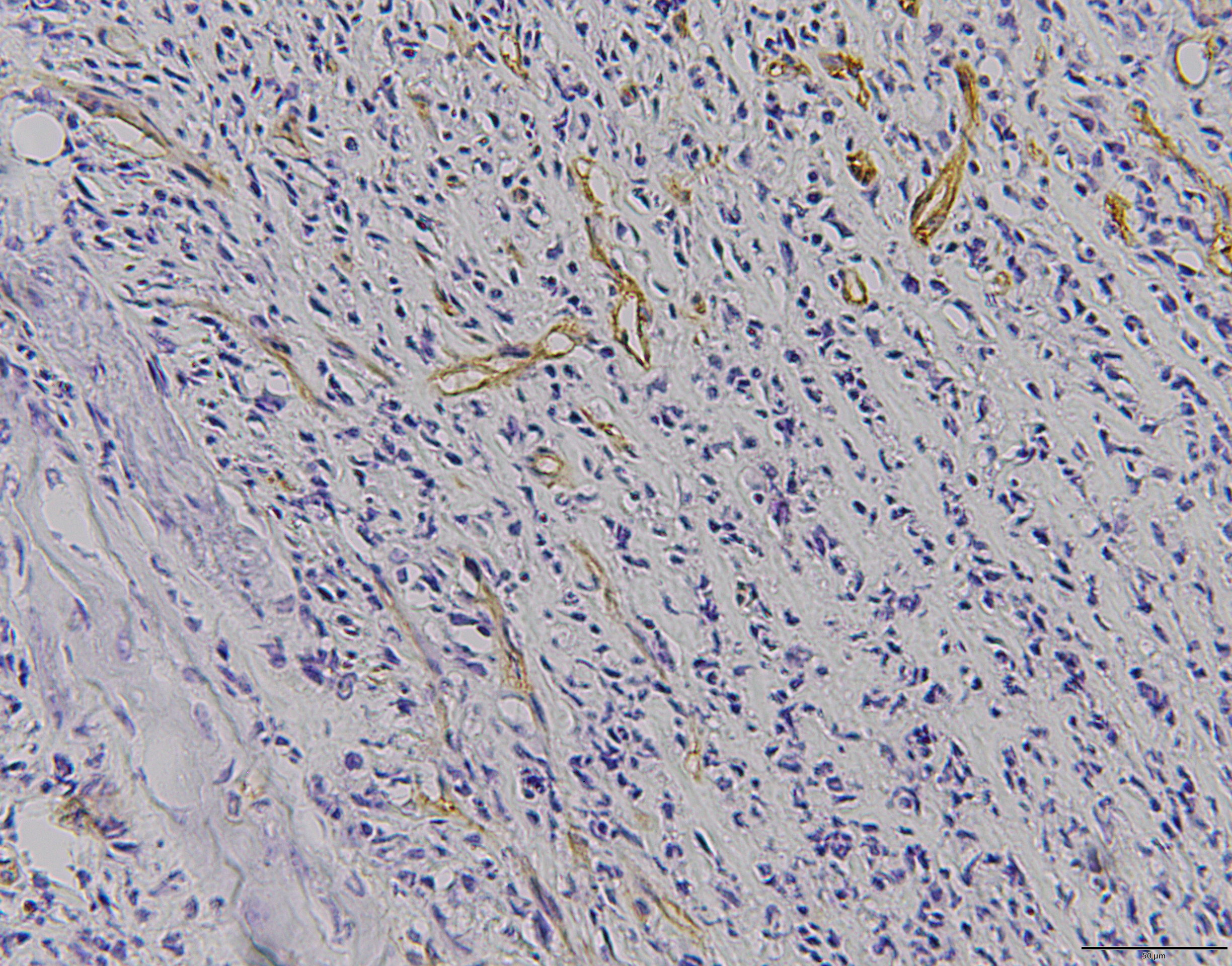

Supplement: S10 File — Fig 5B_wound images. (ZIP) [file pone.0339341.s015.zip › fig 5B_wound images/dbdb estrogen_day 14.tif]

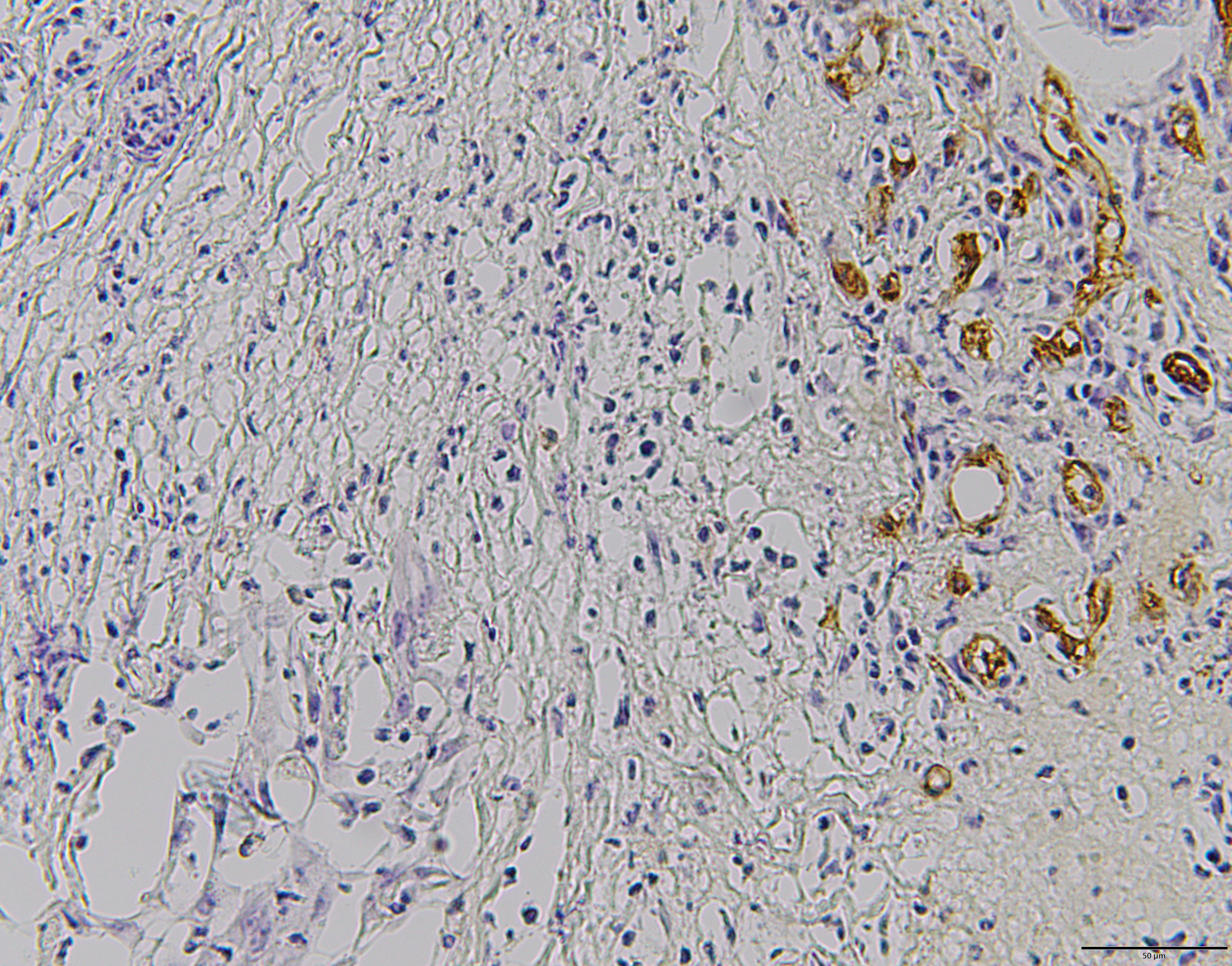

Supplement: S10 File — Fig 5B_wound images. (ZIP) [file pone.0339341.s015.zip › fig 5B_wound images/dbdb estrogen_day 7.tif]

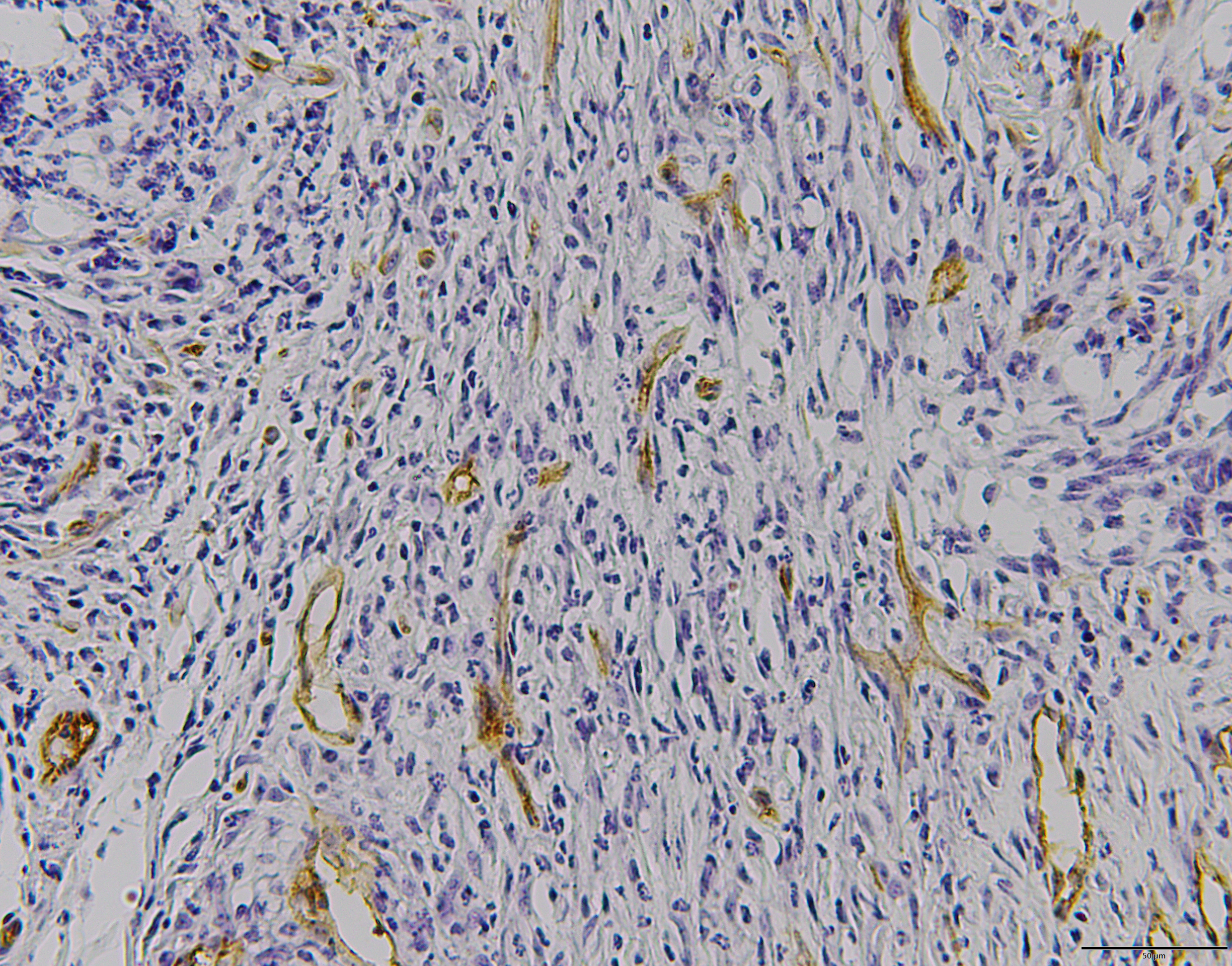

Supplement: S10 File — Fig 5B_wound images. (ZIP) [file pone.0339341.s015.zip › fig 5B_wound images/dbdb_day 14.tif]

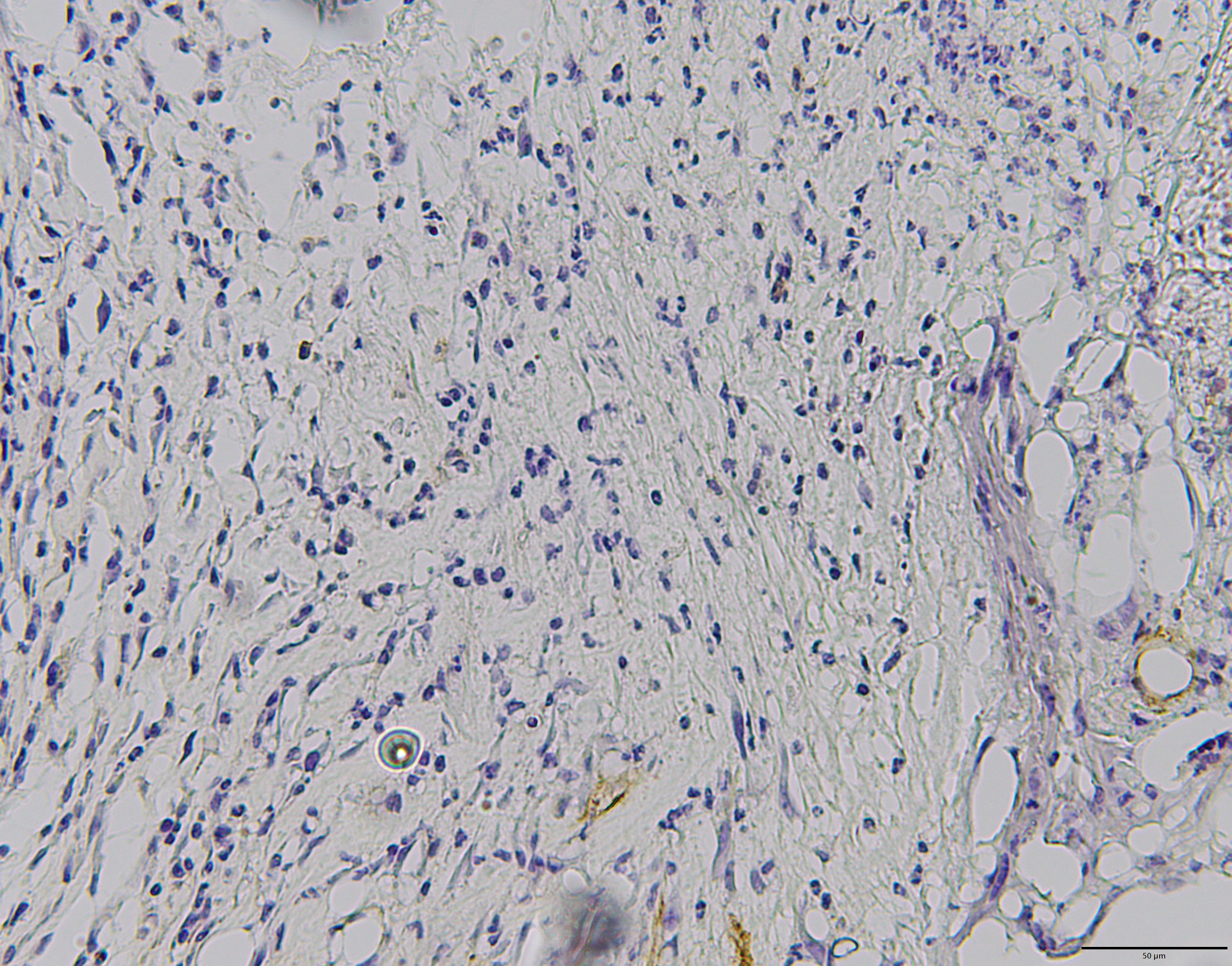

Supplement: S10 File — Fig 5B_wound images. (ZIP) [file pone.0339341.s015.zip › fig 5B_wound images/dbdb_day 7.tif]

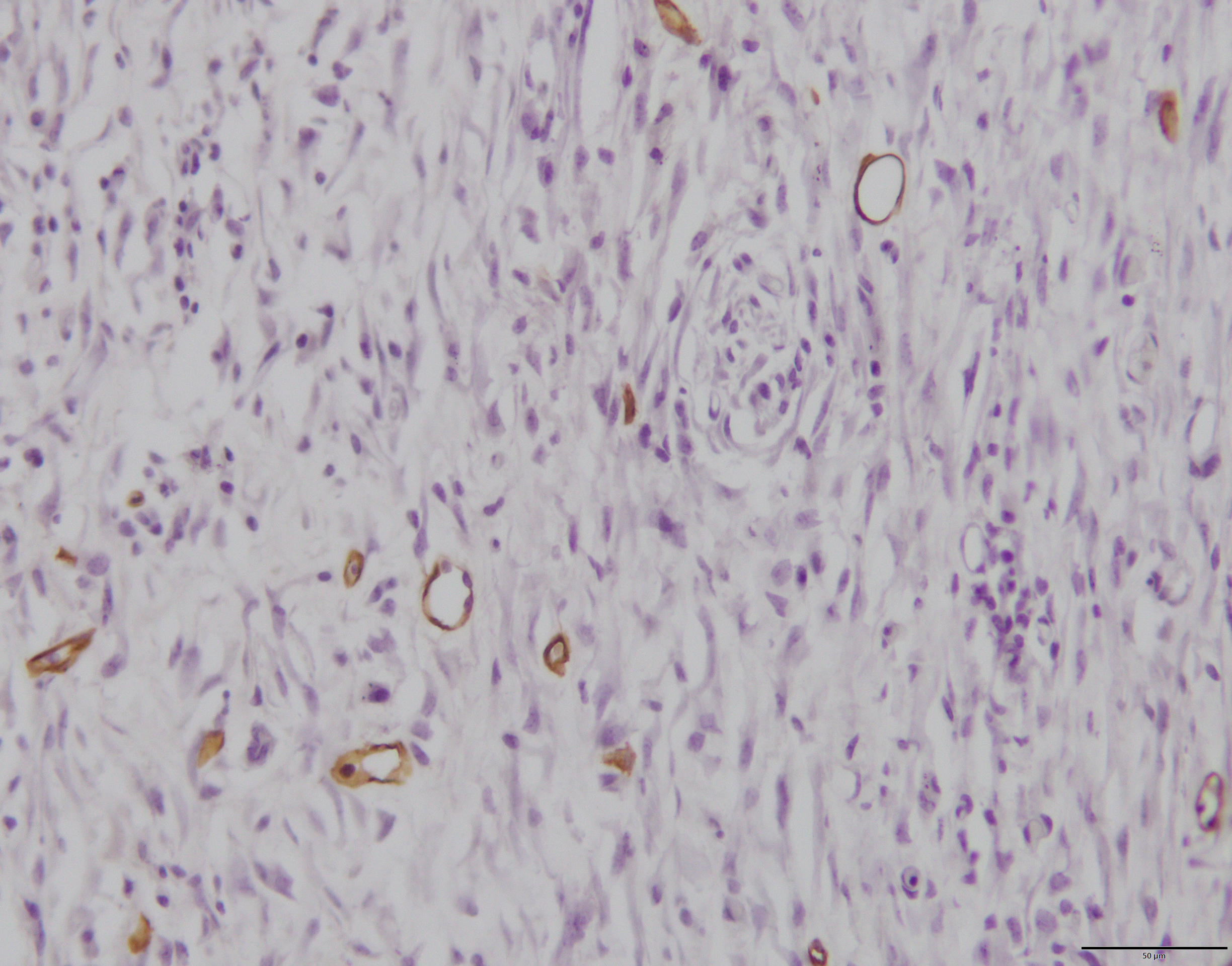

Supplement: S11 File — Fig 5D_wound images. (ZIP) [file pone.0339341.s016.zip › fig 5D_wound images/db+ estrogen_day 14.tif]

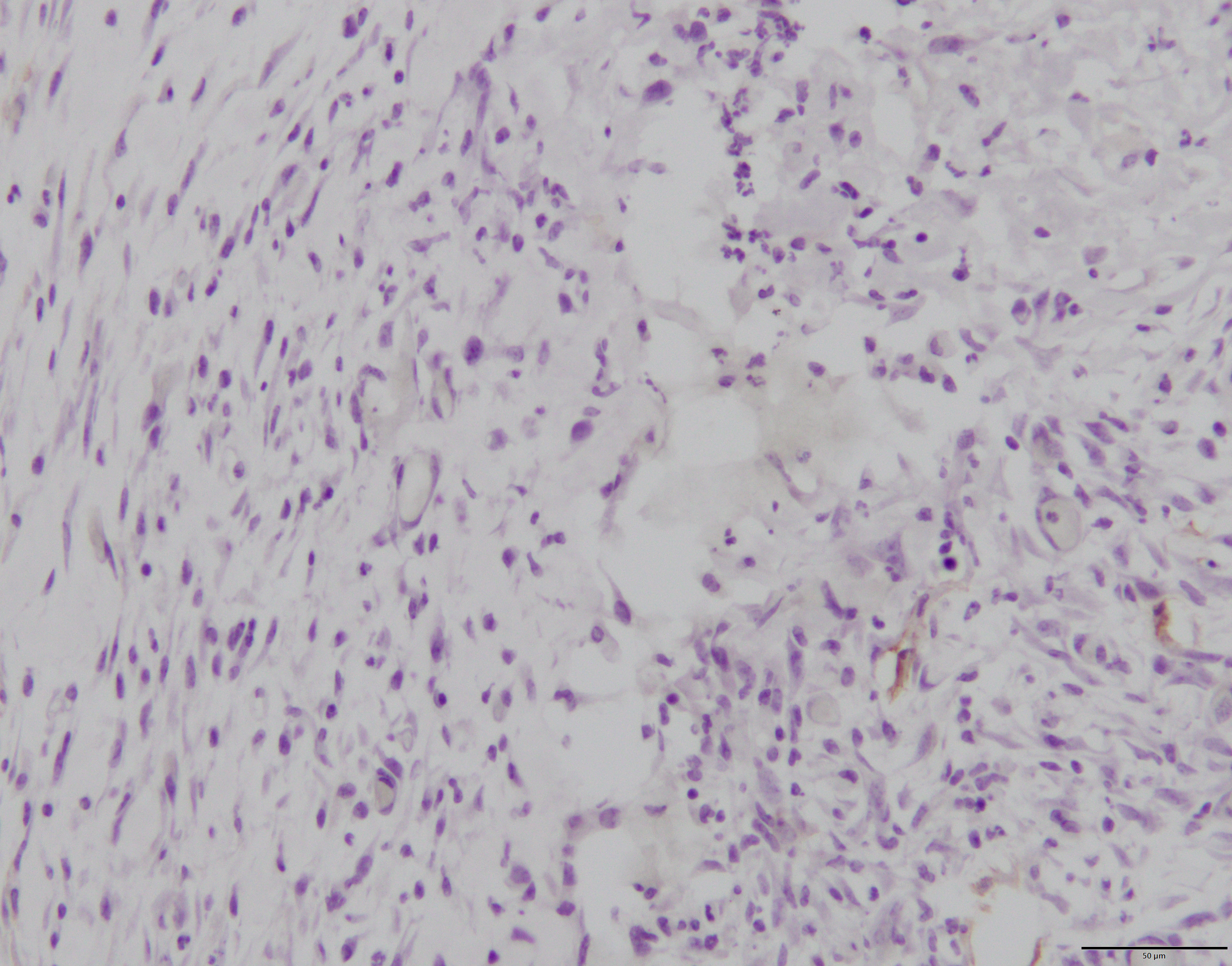

Supplement: S11 File — Fig 5D_wound images. (ZIP) [file pone.0339341.s016.zip › fig 5D_wound images/db+ estrogen_day 7.tif]

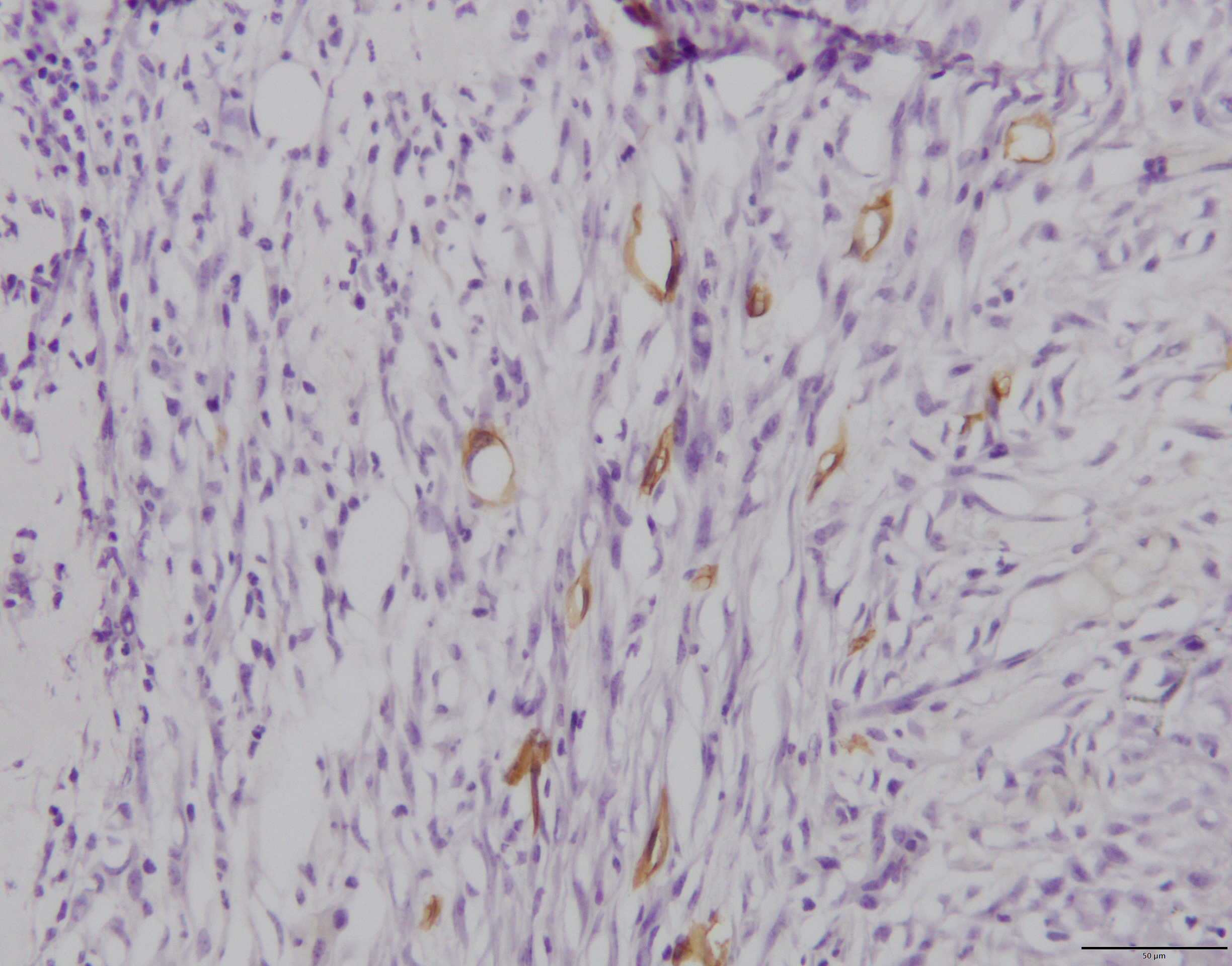

Supplement: S11 File — Fig 5D_wound images. (ZIP) [file pone.0339341.s016.zip › fig 5D_wound images/db+_day 14.tif]

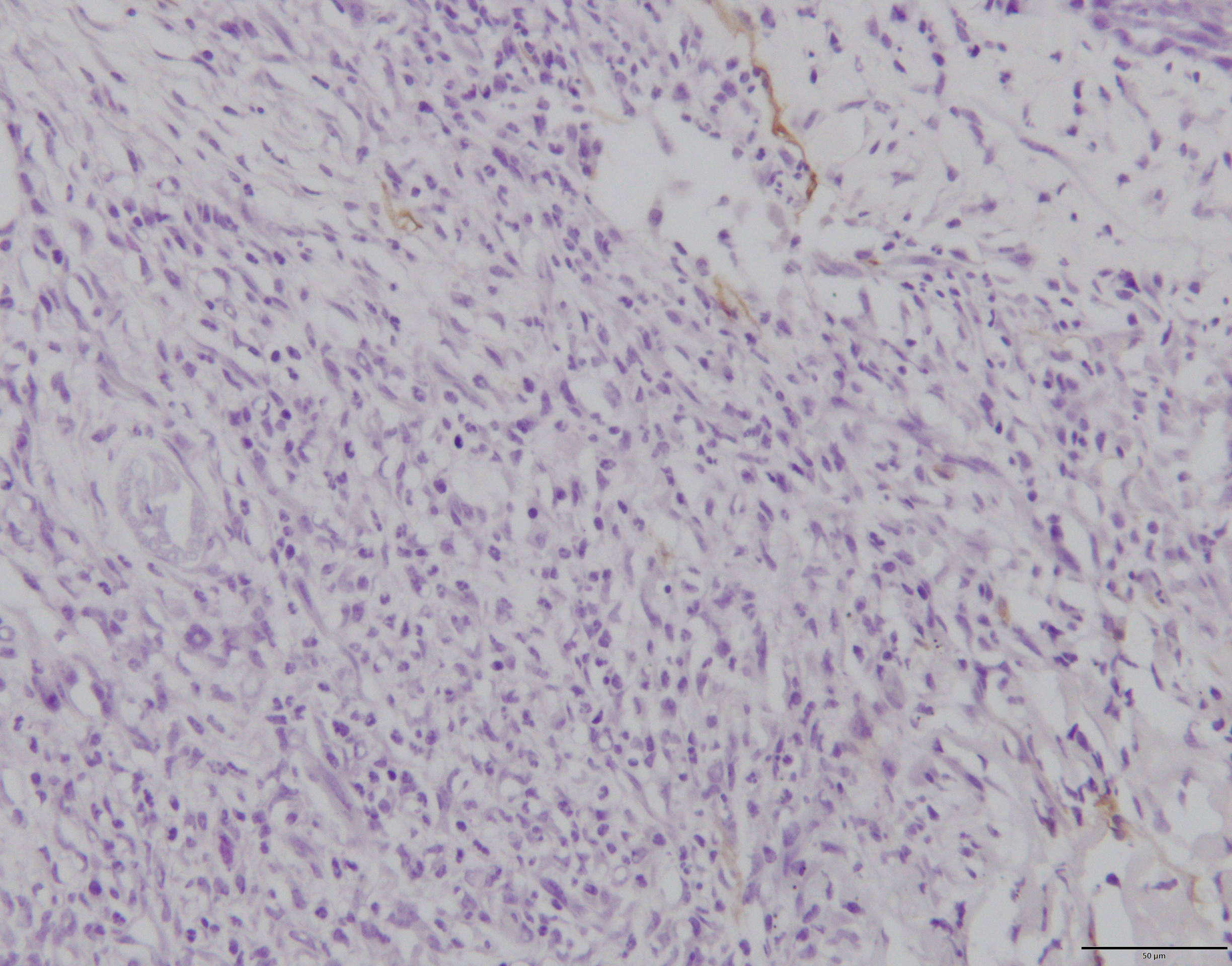

Supplement: S11 File — Fig 5D_wound images. (ZIP) [file pone.0339341.s016.zip › fig 5D_wound images/db+_day 7.tif]

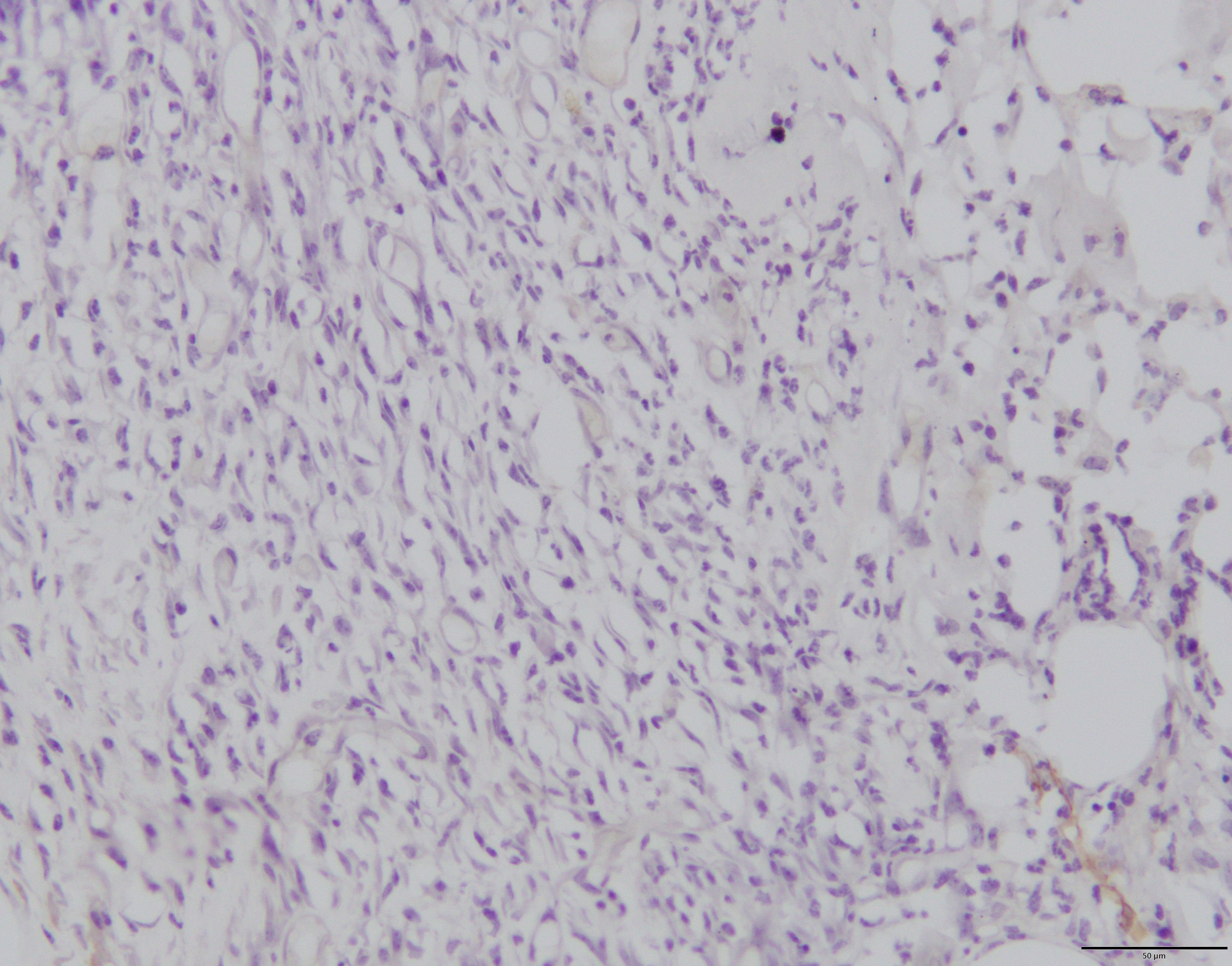

Supplement: S11 File — Fig 5D_wound images. (ZIP) [file pone.0339341.s016.zip › fig 5D_wound images/dbdb estrogen_day 14.tif]

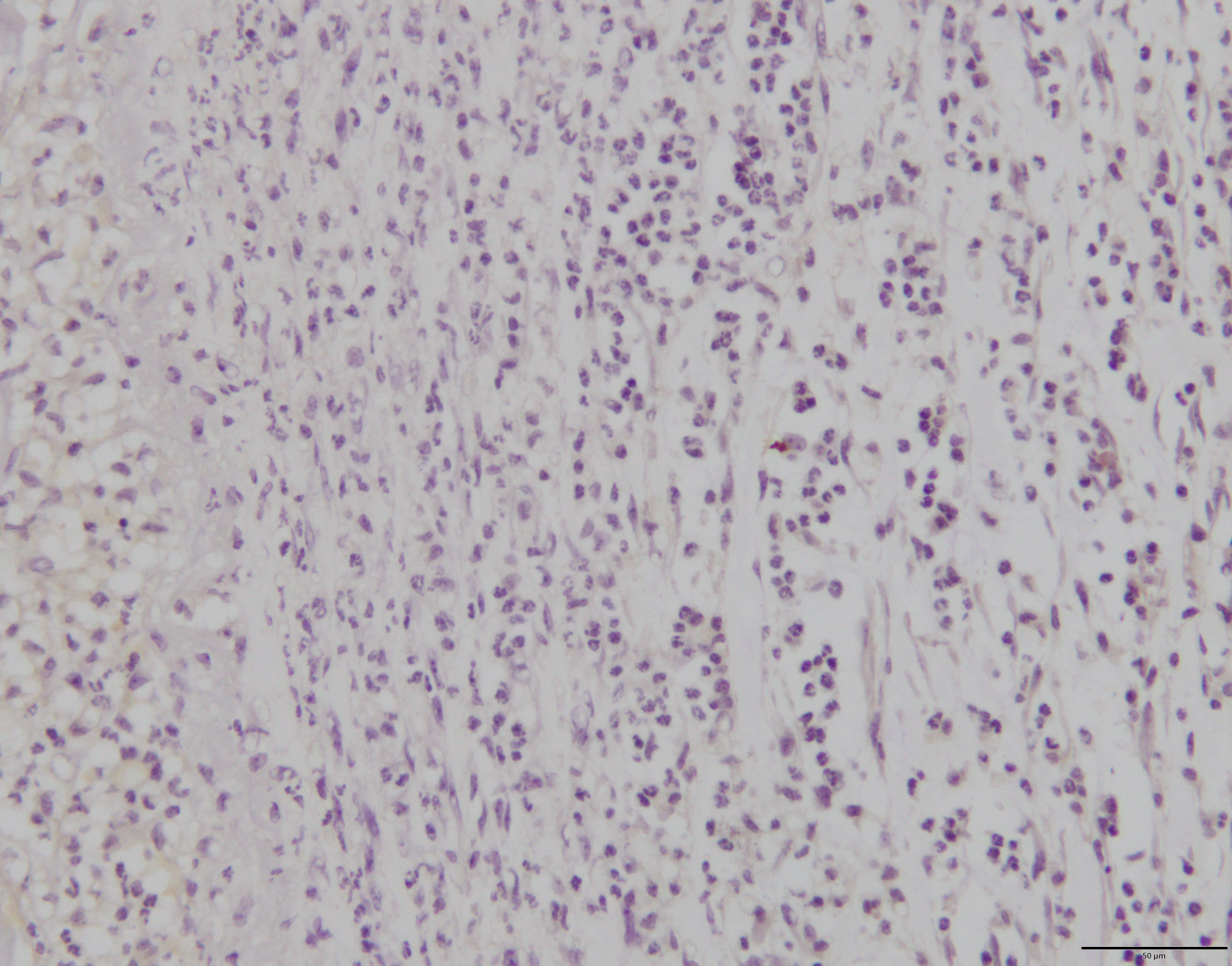

Supplement: S11 File — Fig 5D_wound images. (ZIP) [file pone.0339341.s016.zip › fig 5D_wound images/dbdb estrogen_day 7.tif]

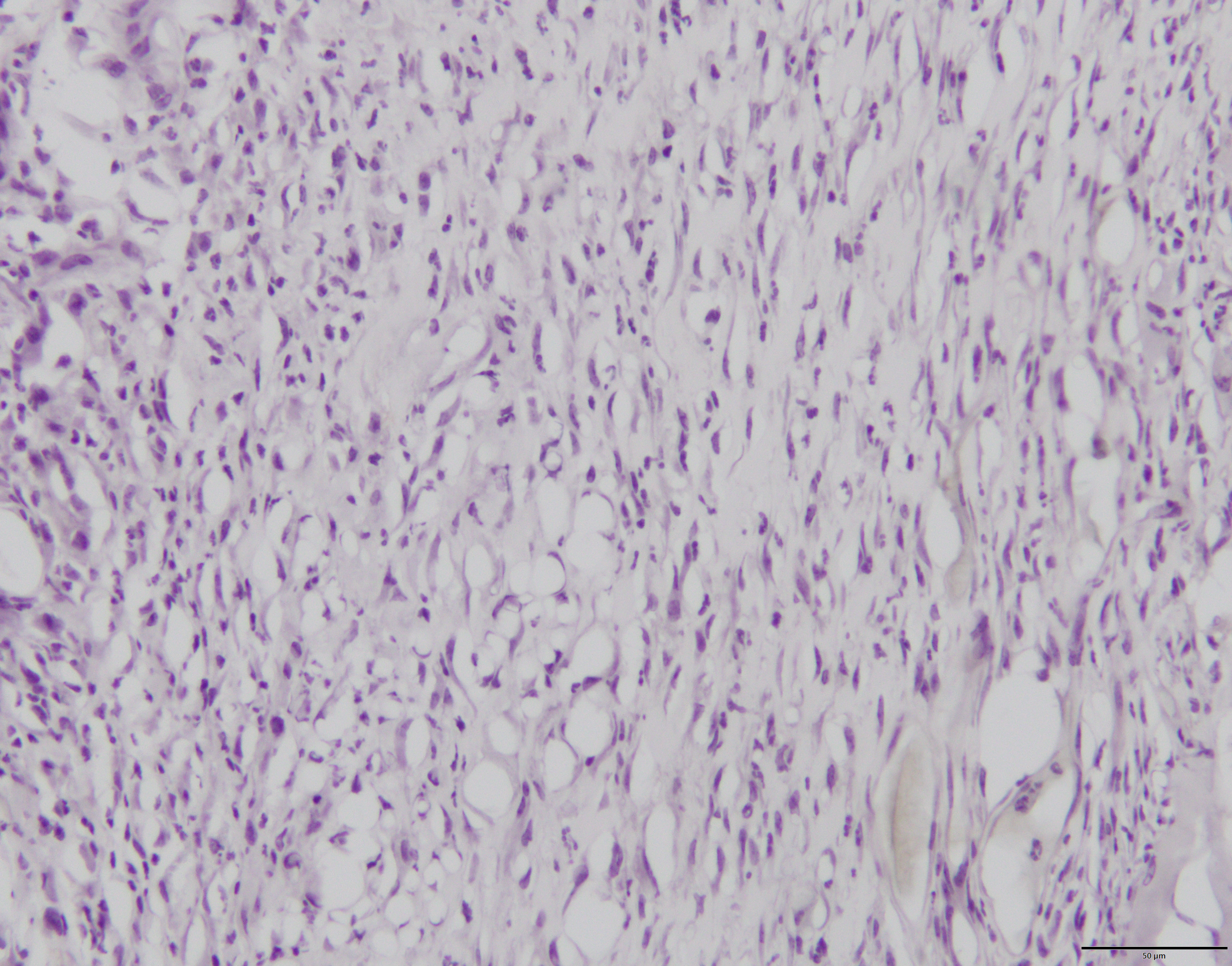

Supplement: S11 File — Fig 5D_wound images. (ZIP) [file pone.0339341.s016.zip › fig 5D_wound images/dbdb_day 14.tif]

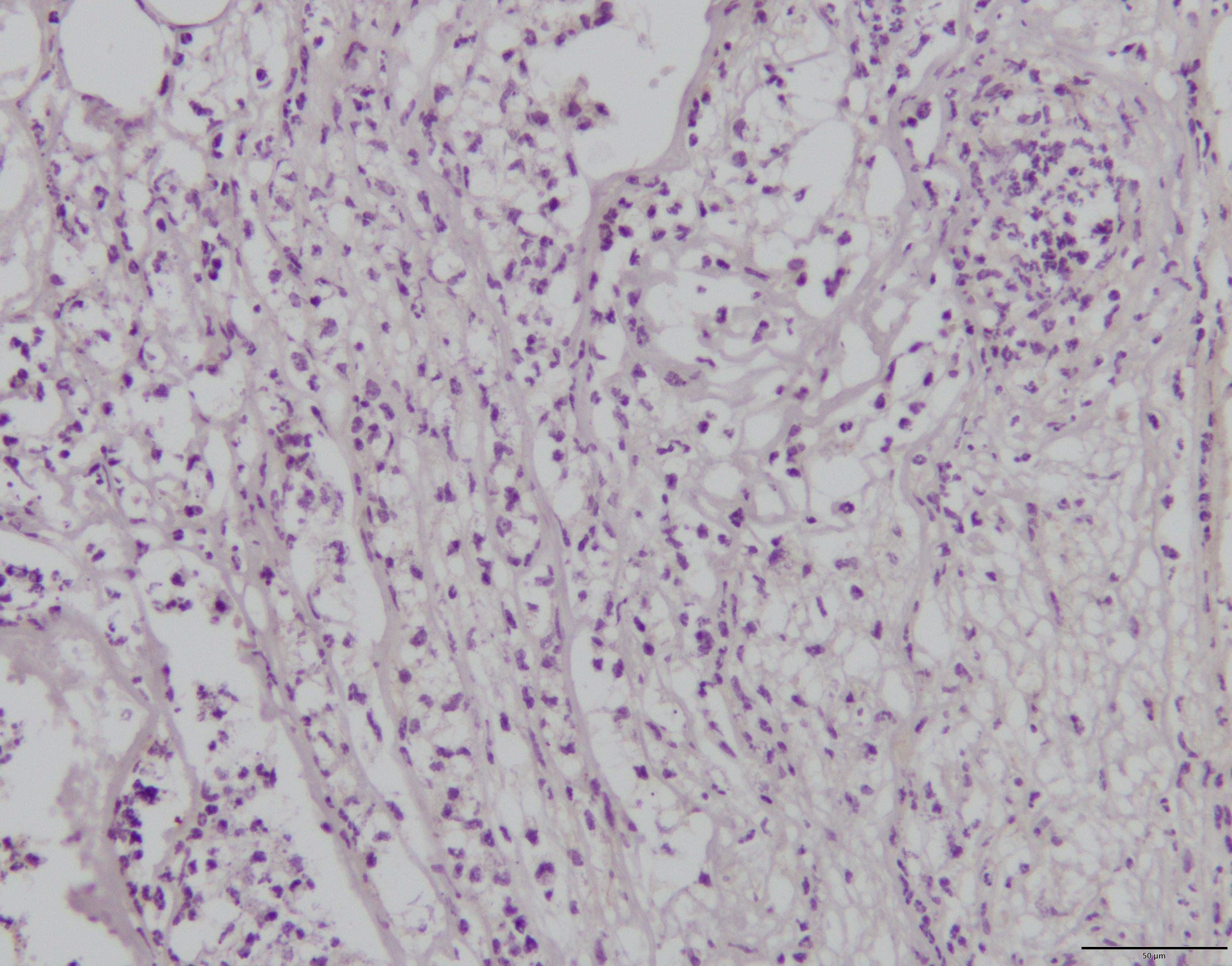

Supplement: S11 File — Fig 5D_wound images. (ZIP) [file pone.0339341.s016.zip › fig 5D_wound images/dbdb_day 7.tif]

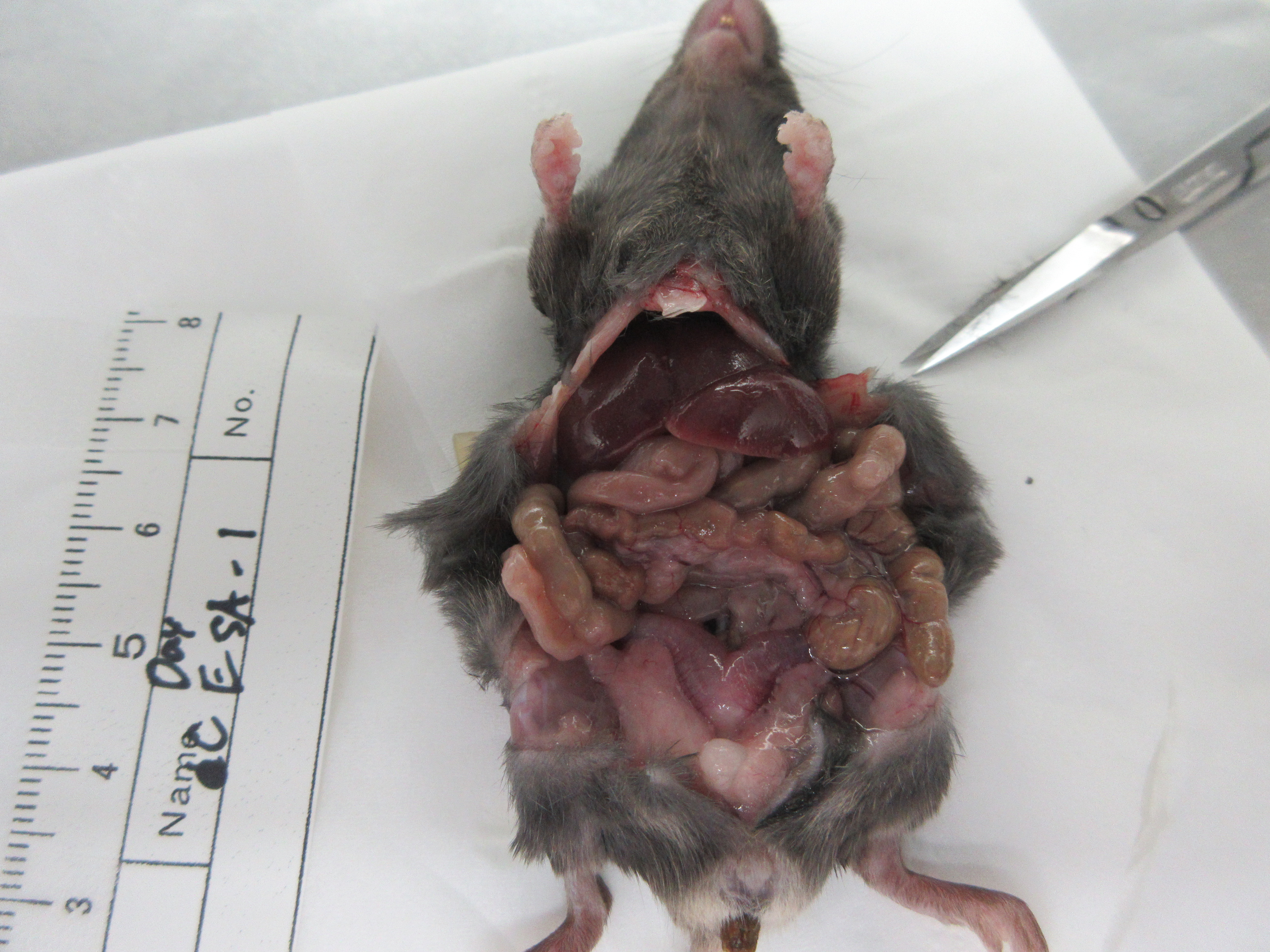

Supplement: S12 File — S4 Fig_uterus. (ZIP) [file pone.0339341.s017.zip › S4 fig_uterus pictures/db+ estrogen.JPG]

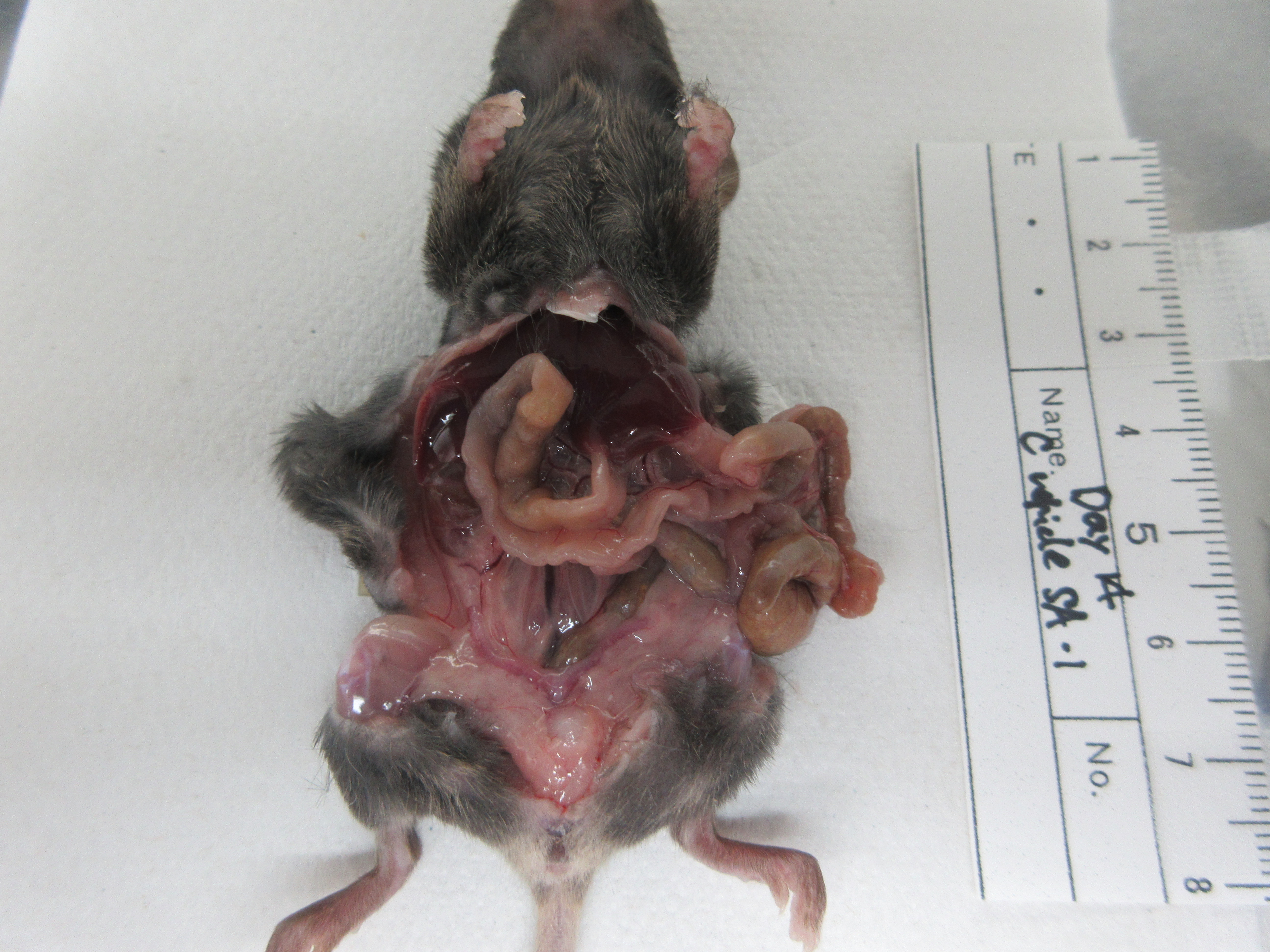

Supplement: S12 File — S4 Fig_uterus. (ZIP) [file pone.0339341.s017.zip › S4 fig_uterus pictures/db+.JPG]

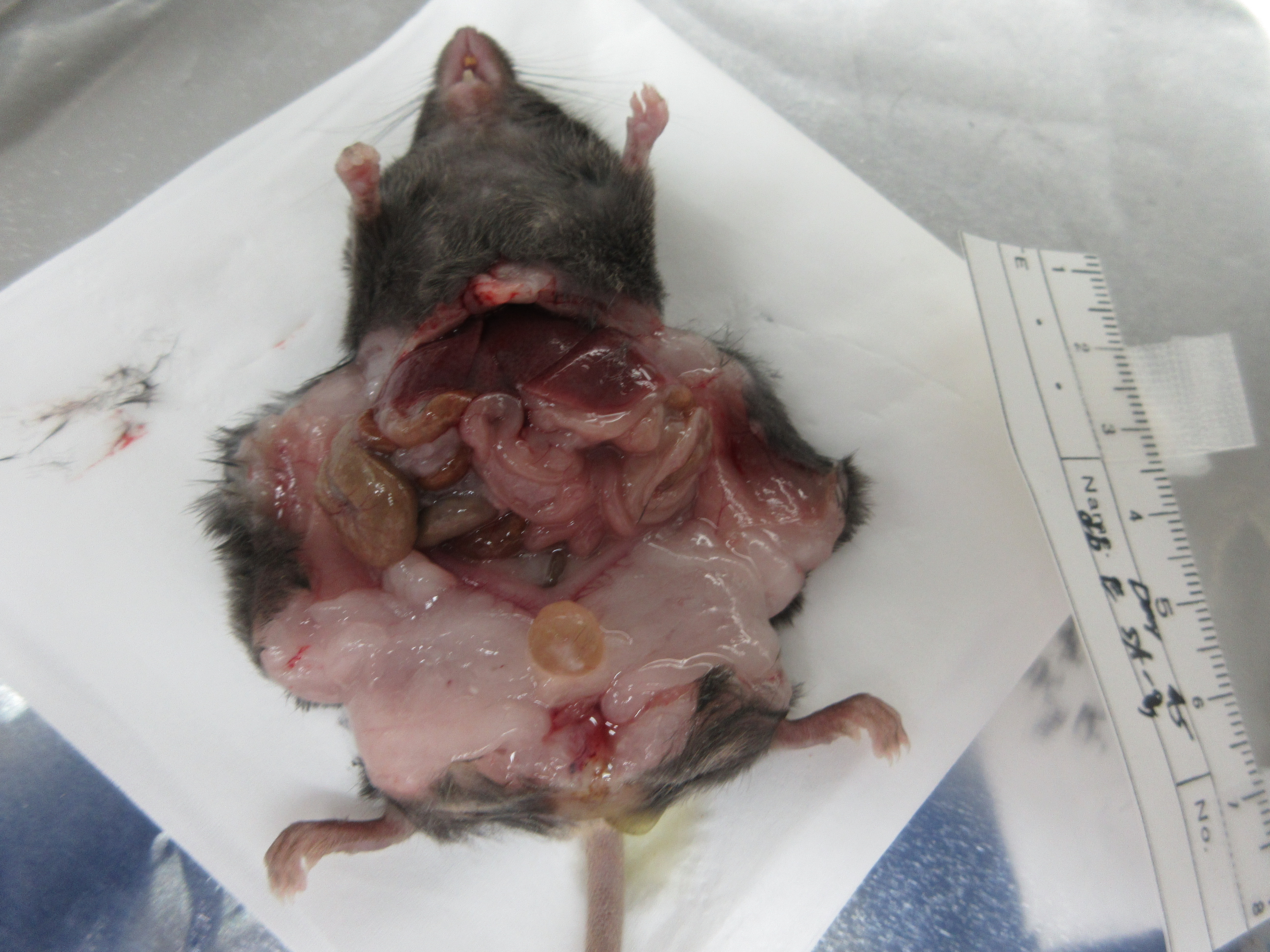

Supplement: S12 File — S4 Fig_uterus. (ZIP) [file pone.0339341.s017.zip › S4 fig_uterus pictures/dbdb estrogen.JPG]

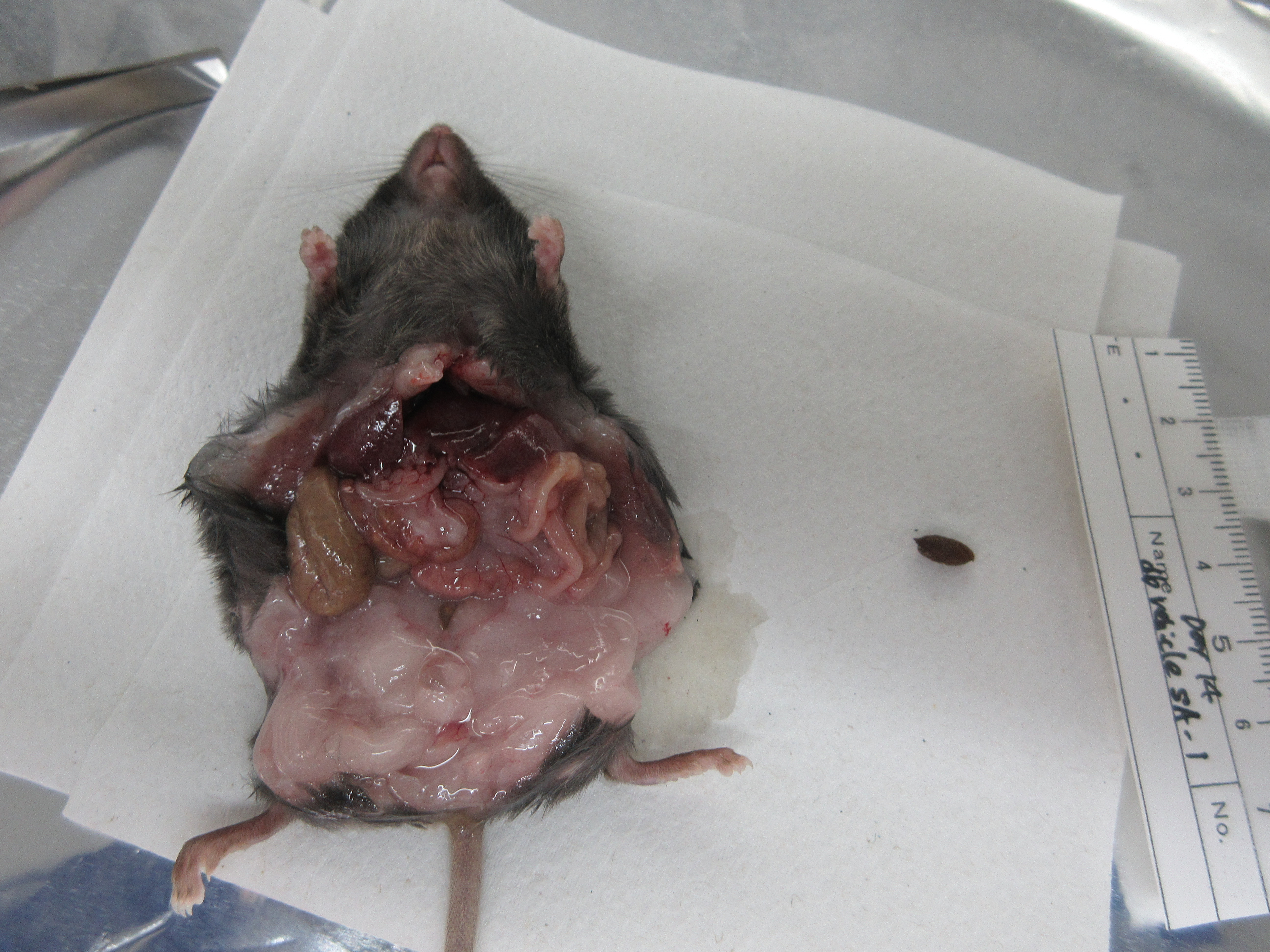

Supplement: S12 File — S4 Fig_uterus. (ZIP) [file pone.0339341.s017.zip › S4 fig_uterus pictures/dbdb.JPG]
